# Supplementary material for: Psychometric evaluation of the Chinese version of the Person-Centred Care Assessment Tool
Source: BMJ Open. 2020 Jul 13;10(7):e031580. doi: 10.1136/bmjopen-2019-031580 (PMC7359066; doi:10.1136/bmjopen-2019-031580)
Supplement: Supplementary data [file bmjopen-2019-031580supp001.pdf]

An Australian Government Initiative

LA TROBE UNIVERSITY

UMEÅ UNIVERSITET

蔡乐 昆明医科大学

Appendix

调查表编号：______________ 医院名称：

调查员： 调查时间： 年 月 日

一. 个人基本情况：

1. 您的出生日期： _______年_______月_______日或年龄_______
2. 性别：①男 ②女
3. 文化程度：①职高 ②中专 ③大专 ④本科 ⑤硕士及以上
4. 民族：①汉族 ②_________族
5. 婚姻状况：①未婚 ②已婚或同居 ③离异 ④丧偶
6. 职务：①护士长 ②总务护士 ③临床护士 ④医生
7. 职称：①护士 ②护师 ③主管护师 ④副主任护师 ⑤主任护师
8. 编制：①合同 ②在编
9. 平均月经济收入：_________元
10. 已从事目前工作_________年，每天平均工作时间为_________小时

二. 以人为本的护理评估工具(P-CAT)

使用说明

该调查问卷主要用来评价人们在一定（环境）机构内，由工作人员提供的以人为本的护理程度。这份问卷主要包括 13 个关于护理的条目。本次调查需要根据您目前所处机构中的亲身经历，判断以下条目与您自身情况相符的程度，并在您认为描述最为贴切的方框内标记“+”号。请务必回答所有条目。如果您不确定，请选择与之最相近的答案。

该测量工具的开发受到澳大利亚政府、拉筹伯大学、J.O 和 J.R.-医疗和科研资助 (ANZ 受托人)、于默奥大学的支持。

©拉筹伯大学
版权声明。在 1968 年《版权法》和 2000 年《版权修正案（数字议程）法案》中有明确规定，未经书面许可，本出版物的任何部分不得进行复制，存储在任何检索系统或以任何方式（包括电子，机械，缩微，影印，录制或其他方式）传播。可在 s.nugent@latrobe.edu.au 或澳大利亚维多利亚州 1231 丰盛路班杜拉校区 ACEBAC 请求版权许可。

	完全不同意<br>1	不同意<br>2	中立<br>3	同意<br>4	完全同意<br>5
1. 我们定期讨论以人为本的护理。	☐	☐	☐	☐	☐
2. 我们有专业的小组讨论护理问题。	☐	☐	☐	☐	☐
3. 我们的正规护理计划中包含了患者的生活史。	☐	☐	☐	☐	☐
4. 医患之间的合作比完成工作更重要。	☐	☐	☐	☐	☐
5. 我们根据患者的需求灵活调整工作。	☐	☐	☐	☐	☐
6. 患者有机会参与到适合个人的日常活动。	☐	☐	☐	☐	☐
7. 我没有时间提供以人为本的护理。	☐	☐	☐	☐	☐
8. 这护理氛围杂乱无章。	☐	☐	☐	☐	☐
9. 我们担心没有舒适的工作环境会对工作质量造成影响。	☐	☐	☐	☐	☐
10. 这个氛围妨碍我提供以人本的护理。	☐	☐	☐	☐	☐
11. 患者的评估居于日常活动。	☐	☐	☐	☐	☐
12. 患者的问题在这里无法解决。	☐	☐	☐	☐	☐
13. 患者可以根据自己的愿望联络外界。	☐	☐	☐	☐	☐

©拉筹伯大学
版权声明。在 1968 年《版权法》和 2000 年《版权修正案（数字议程）法案》中有明确规定，未经书面许可，本出版物的任何部分不得进行复制，存储在任何检索系统或以任何方式（包括电子，机械，缩微，影印，录制或其他方式）传播。可在 s.nugent@latrobe.edu.au 或澳大利亚维多利亚州 1231 丰盛路班杜拉校区 ACEBAC 请求版权许可。
