# Supplementary material for: Psychometric evaluation of the Chinese version of the Person-Centred Care Assessment Tool
Source: BMJ Open. 2020 Jul 13;10(7):e031580. doi: 10.1136/bmjopen-2019-031580 (PMC7359066; doi:10.1136/bmjopen-2019-031580)
Supplement: Author's manuscript [file bmjopen-2019-031580.draft_revisions.pdf]

BMJ Open is committed to open peer review. As part of this commitment we make the peer review history of every article we publish publicly available.

When an article is published we post the peer reviewers' comments and the authors' responses online. We also post the versions of the paper that were used during peer review. These are the versions that the peer review comments apply to.

The versions of the paper that follow are the versions that were submitted during the peer review process. They are not the versions of record or the final published versions. They should not be cited or distributed as the published version of this manuscript.

BMJ Open is an open access journal and the full, final, typeset and author-corrected version of record of the manuscript is available on our site with no access controls, subscription charges or pay-per-view fees (http://bmjopen.bmj.com).

If you have any questions on BMJ Open's open peer review process please email info.bmjopen@bmj.com

BMJ Open

Psychometric evaluation of the Chinese version of the Person-centred Care Assessment Tool

Journal:	*BMJ Open*
Manuscript ID	bmjopen-2019-031580
Article Type:	Research
Date Submitted by the Author:	10-May-2019
Complete List of Authors:	Le, Cai; Kunming Medical University in Kunming,<br>Ma, Ke; The third people's hospital of Kunming, Palliative Care<br>Tang, Pingfen; Kunming Medical University<br>Edvardsson, David; La Trobe University School of Nursing and Midwifery<br>Behm, Lina; Kristianstad University College, Faculty of Health Sciences<br>Zhang, Jie; the third people's hospital of Kunming, palliative care<br>Yang, Jiqun; the third people's hospital of Kunming<br>Fu, Haiyan; the third people's hospital of Kunming<br>Ahlström, Gerd; Lunds Universitet, Health Sciences
Keywords:	Person-centered care, older people, PALLIATIVE CARE, instrument evaluation, factor analysis

SCHOLARONE™
Manuscripts

Psychometric evaluation of the Chinese version of the Person-centred Care Assessment Tool

Running title: Evaluation of the Chinese version of P-CAT

Le Cai, PhD[1], Ke Ma, MD[2], Pingfen Tang, RN[1], David Edvardsson, PhD[3], Lina Behm, PhD[4], Jie Zhang, MD[5] , Jiqun Yang, MD[6], Haiyan Fu, MD[5] and Gerd Ahlström, PhD[7]*

* Corresponding author: Professor Gerd Ahlström, Department of Health Sciences, Faculty of Medicine, Lund University, Box 157, SE-221 00 Sweden. Email: gerd.ahlstrom@med.lu.se

Phone: +46 46 702 747767

[1] PhD, Professor, School of Public Health, Kunming Medical University, Cheng Gong New City, Kunming, China

[2] Affiliated physician, Division of palliative care, the Third People's Hospital of Kunming, China

[3] Professor, Austin Health/Northern Health Clinical Schools of Nursing College of Science, Health and Engineering, La Trobe University, Australia

[4] PhD, Senior Lecturer, Department of nursing educations and integrated health science, Kristianstad University, Sweden

[5] Physician, Division of palliative care, the Third People's Hospital of Kunming, China

[6] Vice Director, Division of palliative care, the Third People's Hospital of Kunming, China

[7] PhD, Professor, Department of Health Sciences, Faculty of Medicine, Lund University, Sweden. ORCID no 0000-0001-6230-7583

Wordcount: 3286

For peer review only - http://bmjopen.bmj.com/site/about/guidelines.xhtml

For peer review only

Abstract

Objective: This study aims to test a Chinese cross-cultural adaptation of the English version of the P-CAT and evaluate its psychometric properties.

Design: P-CAT was translated/back-translated using established procedures. Psychometric evaluation of the Chinese version of the Person-centred Care Assessment Tool.

Setting: Two hospitals covering urban and suburban areas of Kunming, in the Yunnan province of China.

Participants: 152 hospital staff completed the survey.

Main outcome measure(s): Construct validity and reliability, including internal consistency and test-retest reliability, were assessed among a sample of hospital staff.

Results: The factor analysis resulted in a two-component solution that consisted of two subscales. The corrected item-total correlations for all of the items ranged from 0.14 to 0.44, with six items that did not meet the cut-off level for item-total correlation (>0.3). The Chinese P-CAT demonstrated strong reliability, with a Cronbach's alpha of 0.91-0.94 for the scales, and a test-retest reliability coefficient of 0.88 for the overall scale scores. The ICC was 0.92 (95 % CI: 0.90- 0.95).

Conclusion: P-CAT appears to be a promising measure for evaluating staff perceptions of the person-centredness in Chinese hospital environments. The results show that P-CAT can be a useful tool for management of improving quality of healthcare in terms of person-centred care in the Chinese health care context.

Keywords: Person-centered care, older people, palliative care, instrument evaluation, factor analysis

For peer review only - http://bmjopen.bmj.com/site/about/guidelines.xhtml

Article summary

Strengths and limitations of this study

- The first study to validate a translated and culturally adapted but otherwise unmodified version of P-CAT in a Chinese context.
- High response rate (92.1%) in this study.
- Convenience sampling method may limit the ability to generalize the results.
- The Chinese P-CAT has been tested only in this geriatric hospital setting.

For peer review only

For peer review only - http://bmjopen.bmj.com/site/about/guidelines.xhtml

Introduction

Population ageing is occurring globally, and multi-morbidity is highly prevalent, particularly in older people who are generally frequent health care users [1]. The Chinese population is one of the fastest-ageing populations of the world, and the number of people aged 60 years and older is expected to rise from 193 million (13.9 %) to 454 million (32.8 %) between 2013 and 2050 [2]. This rapid increase of older people in China can also be seen in the increasing numbers of older persons admitted to hospitals. The National Health Services survey of China shows that the hospitalization rate of people aged 65 years and older increased significantly from 6.1 % to 19.9 % during the period of 2003 to 2013 [3], and chronic diseases occur more frequently in older people and are more difficult to cure when multiple diseases coexist [4]. Thus, the health care system in China is facing great challenges due to the aging population who require high-quality care from a wider range of multiple care professionals.

Person-centred care (PCC) is now regarded as a valuable method to use when improving long-term care for older people [5] because the quality of care is often described as person-centred. Recently, there is growing interest in applying PCC as a strategy to improve the quality of care for older people, accompanied by developing various instruments to evaluate PCC. One instrument for the assessment of PCC is based on the judgment of health care providers and measures the caring culture valued by professionals; in fact, the person-centredness of a care-setting is linked with how satisfied the staff are with their work and their relationships with patients [6 7].

To date, a number of instruments have been developed to assess the PCC in residential care facilities for older people, and these instruments are mainly based on the opinions of staff working in these facilities, which include the English Person-centred Climate Questionnaire-Staff version (PCQ-S) [8], the Person-centred Care Assessment Tool (P-CAT) [9], the Staff Assessment Person-Directed Care (PDC) [10], the Individualized Care (IC) [11], and the Staff

For peer review only - http://bmjopen.bmj.com/site/about/guidelines.xhtml

Person-Centred Practices in Assisted Living (Staff PC-PAL) [12]. Of these, the tool most commonly used internationally is the P-CAT. The P-CAT questionnaire was designed by Edvardsson and colleagues [9] and is a caregiver-based self-report assessment scale used to measure the extent to which the personnel agrees that a number of pre-defined characteristics/processes of person-centred care are in place within their staff team, unit or nursing home. The P-CAT has a process-focus as evidenced in the following items: altering work routines, including resident life stories, assessment of personal needs, ongoing discussions of residents, etc. It has been validated and used in English [8], Swedish [13 14], Korean [15], Spanish [16], Norwegian [17] and Chinese [18]. By contrast, the PCQ aims to measure the extent to which staff, patients and/or family members agree that the entire atmosphere/climate of a unit/ward is perceived as being welcoming, safe, homely, peaceful, and clean, i.e., the extent to which that place is perceived as person-centred [19].

In China, PCC remains a poorly understood approach that is not generally applied in Chinese geriatric services, and one of the possible barriers is the absence of a dependable and valid instrument to measure and develop person-centred care. A Chinese review of existing instruments for person-centred care in a previous study [18] found that the P-CAT was the most favourable tool to adopt for usage in the Chinese context. A Chinese version of the P-CAT for staff was developed in that study [18], modified and completely changed as they removed two items and added four items, which they had developed themselves to the three scales, making the results incomparable with the original English version. This Chinese version was only validated on staff working in residential care for patients with dementia because P-CAT was originally intended for use as a research instrument to measure the presence of person-centred care in this context [9]. To promote the improvement of the quality of care through the implementation of person-centred care in a broader Chinese health care context, further testing and cross-validation of the P-CAT with new samples from Chinese geriatric hospital settings

For peer review only - http://bmjopen.bmj.com/site/about/guidelines.xhtml

are necessary to ensure that this measure is applicable in different settings for older persons. Therefore, the aim of this study was to make a cultural adaptation of the English version of the P-CAT for Chinese nurses as well as evaluate the psychometric properties of the newly translated Chinese version applied in geriatric palliative care.

Methods

This study tested and evaluated the Chinese version of the P-CAT in Mandarin, the most common language in China, on nurses in a hospital context.

The original English instrument

P-CAT measures the extent to which long-term aged care staff rate their settings to be person-centered. The English P-CAT questionnaire has been validated in an Australian sample of nursing home staff and was shown to have satisfactory psychometric properties [9]. The self-reported questionnaire exists in both a staff version and a version for older persons. In this study, the version for staff was used. The staff version comprises 13 items covering three subscales (the Extent of personalizing care, the Amount of organizational support, and the Degree of environmental accessibility). The Extent of personalizing care was measured through items 1-7, Amount of organizational support was measured through items 8-11, and Degree of environmental accessibility was measured through items 12-13. A 5-point Likert-type scale ranging from 1 (strongly disagree) to 5 (strongly agree) was used for scoring. Items 8–12 were negatively worded and the responses were reverse-scored before analysing the data and calculating a total score. Aggregated scores were calculated by means of simple sum scores at the subscale and total scale levels. The total score ranged between 13 to 65 and a higher score indicated a higher degree of Person-centredness.

For peer review only - http://bmjopen.bmj.com/site/about/guidelines.xhtml
For peer review only

Translation and cross-cultural adaptation of the P-CAT

The process of translation and cross-cultural adaptation followed International test commission guidelines [20 21]. Three native Chinese speakers conducted independent forward translations from English to Chinese; two had a Public Health degree and the last translator was a senior nurse who had worked in a department of palliative care for over ten years. First, the translators agreed upon a consensus version after discussing the different versions. Then it was back-translated into English by two different translators who were bilingual and who had no knowledge of the procedures of the forward translation. After that, an expert committee was held to resolve discrepancies, which was composed of all the translators, two geriatric physicians, one senior nurse, and two university professors. The version was modified by comparing the original version with the back-translated version. During the consensus, the expert committee culturally adapted a few phrases to fit the Chinese setting, resulting in a pre-final version. The final version of the Chinese P-CAT was generated after pre-testing through face-validity where ten staff members from a municipal hospital in Kunming, which consisted of ten female nurses, with a mean age of 31.2 years (SD ±8.3). The pre-testing resulted in no changes. The ten staff members who participated in the face-validity of the pre-final version did not participate in the later study.

Sample, participants and data collection

The sample was selected to cover hospital nurses working both in urban and suburban areas of Kunming, the capital of the Yunnan province in southwest China. Two municipal hospitals in Kunming were chosen through a convenience sampling method: one hospital from the Wu Hua district (urban area of Kunming) and one from the Guan Du district (a suburban area of Kunming). The following criteria for inclusion were used: municipal level hospital with a department of palliative care. All nurses (n=165) on duty (all shifts on the day of the data

For peer review only - http://bmjopen.bmj.com/site/about/guidelines.xhtml

collection) at the palliative care departments of the two hospitals were deemed to be eligible for participation and were asked to participate in the study. The sample size of the study was determined by the criteria suggested by Reise et al. [22], in which five to ten persons for each item of the scale and sample sizes of 100 are often adequate.

The questionnaires were distributed by two graduate students to all participants, and the completed questionnaires were then anonymously collected on site. In addition to the P-CAT scale, demographic characteristics of the participants were also collected by a questionnaire that assessed age, gender, level of education, ethnicity and position. Finally, 152 nurses gave their informed consent and completed the Chinese P-CAT questionnaire. This gives an overall response rate of 92.1 %. The 152 participants were asked to participate in both the test and retest assessments. The retest was conducted between one and two weeks after the initial test.

For peer review only

Psychometric evaluation

Only one item in one questionnaire had a missing value and was replaced with the mean value of the item for the entire group [23]. An exploratory factor analysis (EFA) with extraction of principal components by both varimax and oblique orthogonal rotation was used to evaluate construct validity. No difference was found between these two methods in the analysis and thus only the results of the analysis with varimax orthogonal rotation are presented. Bartlett's test of sphericity and the Keiser-Meyer-Olkin (KMO) measure were determined to check the appropriateness of the factor analysis. In order to assess whether the correlation between items was adequate based on a criterion of $p < 0.0001$, Bartlett's test of sphericity was used, and a criterion of KMO ≥ 0.70 was used to indicate sample adequacy. When the Kaiser criterion of eigenvalues was ≥ 1, factors were extracted. A cut-off of 0.50 was applied to determine item loadings on each individual component [24].

The internal consistency and test-retest reliability were evaluated. The internal consistency

For peer review only - http://bmjopen.bmj.com/site/about/guidelines.xhtml

for the total and subscale scores was estimated using Cronbach's alpha coefficient, and Cronbach's alpha ≥0.50 was used as acceptable internal consistency reliability [25]. The item-total correlations were also computed, and the cut-off score for acceptable item-total correlations was set to between 0.3 and 0.8 to ensure moderate correlation and avoid item redundancy. Test-retest reliability was examined with the intra-class correlation (ICC) and Pearson's correlation coefficient (r), where an ICC >0.80 was taken to indicate satisfactory reliability [26]. The paired t-test (2-tailed) was used to verify whether the mean scores from the test and retest differed.

The results of the PCA indicate that three factors with eigenvalues above 1 produced a three-component rotated solution that explained 60.2 % of the total variance in the data. After direct varimax orthogonal rotating, the structure matrix showed that the first component comprised of seven items, the second component comprised four items, and the third component comprised two items. With regard to the internal consistency reliability, the Cronbach's alpha coefficient of the 13-item Chinese P-CAT was 0.65 for the total scale, 0.67 for the first subscale, 0.85 for the second subscale and 0.42 for the third subscale. Since subscale three failed to meet the reliability cut-off of 0.5, the analysis was re-run by forcing the EFA into 2 factors.

Statistical significance was determined based on two-tailed P values of <0.05. All data analyses were performed using SPSS 17.0 software.

Results

Demographic characteristics of the study group

As described in Table 1, the sample consisted of only female nurses, with a mean age of 30.7 years (SD ±8.7, range 19-53 years). Of these, 59.9 % of participants were registered nurses, followed by enrolled nurses (39.4 %). In total, 25.7 % of the participants had a Bachelor's

For peer review only - http://bmjopen.bmj.com/site/about/guidelines.xhtml

degree or higher. More than one-fourth of the participants reported a minority ethnicity.

Table 1 Demographic characteristics of the study sample (n=152)

**Characteristics**	**n (%)**
Gender	
Female	152 (100.0)
Male	0 (0.0)
Age (years)	
18-30	96 (63.2)
31-39	22 (14.5)
⩾40	34 (22.4)
Level of education	
High school	14 (9.2)
Secondary school	39 (25.7)
Junior college	60 (39.5)
Bachelor	39 (25.7)
Ethnicity	
Han	111 (73.0)
Minorities	41 (27.0)
Position	
Registered nurse	91 (59.9)
Enrolled nurse	60 (39.4)
Senior nurse	1 (0.7)

For peer review only

Construct validity

Bartlett's test of sphericity was significant (754.99, $p < 0.0001$) and the KMO measure were satisfactory (0.78), this indicates that correlations between items were sufficient to conduct a principal component analysis (PCA). As shown in Table 2, the results of the PCA indicate that two factors with eigenvalues above 1 produced a two-component rotated solution

For peer review only - http://bmjopen.bmj.com/site/about/guidelines.xhtml

that explained 50.4 % of the total variance in the data. After direct varimax orthogonal rotating, the structural matrix showed that the first component comprised nine items (loadings between 0.16 and 0.79) and the second component comprised four items (loadings between 0.71 and 0.89). The two sub-scales were labeled as (1) Extent of personalizing care, and (2) Amount of organizational and environmental support.

Table 2 Rotated component matrix for PCA of the 2-factor Chinese P-CAT

**Item number**	**Item content**	**Factor loadings**	
		**Subscale 1: Extent of personalizing care**	**Subscale 2: Amount of organizational and environmental support**
1	We often discuss how to provide person-centred care.	**0.73**	0.008
2	We have formal team meetings to discuss residents' care.	**0.79**	0.045
3	The life history of the residents is formally used in the care plans we use.	**0.76**	0.028
4	The quality of the interaction between staff and residents is more important than getting the tasks done.	**0.72**	-0.087
5	We are free to alter work routines based on residents' preferences.	**0.67**	0.117
6	Residents are offered the opportunity to be involved in individualized	**0.54**	-0.109

For peer review only

	everyday activities.		
7	Assessment of residents' needs is undertaken on a daily basis.	**0.17**	0.26
8	I simply do not have the time to provide person-centred care.	-0.208	**0.78**
9	The environment feels chaotic.	-0.096	**0.89**
10	We have to get the work done before we can worry about a homelike environment.	**0.64**	-0.364
11	The organization prevents me from providing person-centred care.	-0.094	**0.89**
12	It is hard for residents in the facility to find their way around.	-0.036	**0.71**
13	Residents are able to access outside space as they wish.	**0.35**	-0.087
Total variance explained (%)	50.4 (total 3 subscales)	30.18	20.24
Cronbach's alpha	0.92 (total 13 items)	0.94	0.91

Note: Bold number means included in the scale

Reliability

With regard to the internal consistency reliability, the Cronbach's alpha coefficient of the 13-item Chinese P-CAT was 0.92 for the total scale, 0.94 for the subscale Extent of personalized care, and 0.91 for the Amount of organizational and environmental support subscale (Table 2), indicating that the overall internal consistency reliability was strong. The

For peer review only - http://bmjopen.bmj.com/site/about/guidelines.xhtml

For peer review only

corrected item-total correlations for all items ranged from 0.14 to 0.44 (Table 3), with six items (items 6, 7, 9, 11, 12 and 13) failing to meet the commonly suggested cut-off level for item-total correlation (>0.3).

Table 3 Item performance and reliability test of the Chinese P-CAT

**Item number**	**Item content**	**Mean±SD**	**Corrected item- total correction**	**Cronbach's alpha if item deleted**
1	We often discuss how to provide person-centred care.	4.19±0.70	0.36	0.62
2	We have formal team meetings to discuss residents' care.	4.32±0.66	0.44	0.61
3	The life history of the residents is formally used in the care plans we use.	4.24±0.73	0.41	0.62
4	The quality of the interaction between staff and residents is more important than getting the tasks done.	4.50±0.65	0.32	0.63
5	We are free to alter work routines based on residents' preferences.	4.20±0.77	0.42	0.61
6	Residents are offered the opportunity to be involved in individualized everyday activities.	3.86±0.82	0.23	0.64
7	Assessment of residents' needs is undertaken on a daily basis.	2.30±1.10	0.19	0.65
8	I simply do not have the time to provide person-centred care.	3.89±0.82	0.35	0.62
9	The environment feels chaotic.	4.11±1.00	0.15	0.65

For peer review only - http://bmjopen.bmj.com/site/about/guidelines.xhtml

10	We have to get the work done before we can worry about a homelike environment.	1.74±0.62	0.37	0.62
11	The organization prevents me from providing person-centred care.	4.14±0.92	0.17	0.65
12	It is hard for residents in the facility to find their way around.	2.22±0.88	0.26	0.64
13	Residents are able to access outside space as they wish.	3.89±0.82	0.14	0.65

In Table 4, the results of Pearson's correlation coefficient analysis indicated that the Chinese P-CAT had a high correlation between the test and retest on all scale levels: on the sub-scales Extent of personalizing care (r=0.90, P<0.01) and Amount of organizational and environmental support (r=0.88, P<0.01) and on the overall scale (r=0.88, P<0.01). The paired t-test shows no statistically significant differences between the mean scores of the P-CAT in the test and retest ratings. The ICC of the total score between the test and retest was 0.92, providing further support that the scale had satisfactory test-retest reliability.

Table 4 Test-retest reliability of the Chinese P-CAT

**Scale dimension**	**1st test (Mean±SD)**	**2nd test (Mean±SD)**	**P**	**Pearson correlation coefficient (r)**	**ICC (95 % CI)**
Extent of personalizing care	33.35±3.59	33.13±3.33	0.09	0.90	0.94 (0.92, 0.96)
Amount of organizational and environmental support	14.41±1.72	14.34±1.74	0.34	0.88	0.91 (0.87, 0.93)
Overall scale	47.76±3.70	47.46±3.65	0.07	0.88	0.92 (0.90, 0.95)

Discussion

In this study, the English P-CAT was culturally adapted into a Chinese version. During the translation of the English P-CAT into Chinese, a few minor cultural discrepancies were

For peer review only - http://bmjopen.bmj.com/site/about/guidelines.xhtml
For peer review only

encountered, and two items of the P-CAT have therefore been modified accordingly. In this case, to use words closer to the Chinese cultural context and more applicable to a hospital environment, the phrase 'the resident' was changed to 'the patient' and 'free' was replaced by 'flexible'. After cross-cultural adaptation, the pre-testing of the Chinese version of the P-CAT revealed that the items were clearly understood.

This Chinese version of the P-CAT showed a different subscale structure compared to the original English scale, i.e., a structure with three subscales (Extent of personalizing care, Amount of organizational support and Degree of environmental accessibility) consisting of 13 items, whereas it showed the same subscale structure as the Norwegian scale, i.e., which had two subscales (Extent of personalizing care and Amount of organizational and environmental support). The 13 items were mostly in concordance with the Norwegian version whereas only item 10 was connected to the first subscale [17]. The 2-factor structure has also been confirmed in the Swedish version of the P-CAT based on a large cross-sectional sample of 1465 staff members from 195 residential elderly care units [14] and in a more recent study based on 142 staff working in residential elderly care units and 182 staff working at health care centres or home care centres and social services [13]. Moreover, in this study the Chinese P-CAT showed strong internal consistency reliability with a Cronbach's alpha of 0.92 for the total scale, indicating that the Chinese P-CAT matched well with the characteristics and structure of the Norwegian version. However, the different subscale structures of this Chinese version of the P-CAT compared to the original English scale may reflect a difference in sample characteristics of staff and the cultural context. The test-retest reliability assessed by ICC was excellent for overall Chinese P-CAT score (0.92) and both subscales (0.94 and 0.91), and the excellent test-retest reliability of the Chinese P-CAT was further evidenced through a Pearson's correlation coefficient (r) of 0.90 for the total scale, 0.88 for the Extent of personalizing care subscale and 0.88 for the Amount of organizational and environmental support subscale. The results of

For peer review only - http://bmjopen.bmj.com/site/about/guidelines.xhtml

Pearson's correlation coefficient (r) for the total scale in the Chinese version were higher than the English (0.66), Swedish (0.75), Norwegian (0.83) and previous Chinese version (0.68), which indicates that this Chinese P-CAT may have a stronger test-retest reliability compared to other language versions of the P-CAT.

In this Mandarin version of the Chinese P-CAT, six items (6, 7, 9, 11, 12 and 13) did not meet the commonly suggested cut-off level for item-total correlation (>0.3). In the previous Chinese version of the P-CAT [18], items 7 and 9 were deleted due to the low value of factor loading (<0.3), and four newly developed items were added to the scale. Item 7 was also lower in this study than the recommended factor loading; however, we decided to include this item in the subscale 1, in agreement with the original English version, although this item had a slightly higher loading in factor 2. Because these two Chinese versions of the P-CAT were evaluated in different health care settings (hospital environments and residential care facilities), there is no firm consensus regarding the psychometric properties of the Chinese P-CAT, and the findings suggest that the Chinese version requires further investigation and analysis in other samples and settings.

In contrast to the low response rate of 21 % in the development study of the original English version P-CAT [9], our study showed a high response rate of 92.1 %. However, some limitations should be noted in our study. First, the present study was conducted only in a hospital context, which may limit the ability to generalize the results to staff working in other health care contexts. Second, criterion-related validity was not assessed as a gold standard scale for the measurement of person-centred care in hospitals; however, this does not exist because of the lack of previous studies in hospital contexts. Third, the content validity of the P-CAT was only considered along with the procedures of cross-cultural adaptation in this study.

Conclusion

For peer review only - http://bmjopen.bmj.com/site/about/guidelines.xhtml

The results indicated that the 13-item Chinese P-CAT is a cross-culturally adapted version of the English P-CA that shows strong internal consistency and test-retest reliability and appears to be a promising measure for evaluating staff perceptions of person-centredness in a Chinese hospital setting. The results of published psychometric evaluations indicate the use of two scales in the P-CAT as was determined in this study. However, the authors suggest that the measurement properties of the Chinese version should be further evaluated in other samples and in different health care settings. An additional issue for future studies is to evaluate this instrument as a summative measurement with cut-off values for different levels of person-centred care. This may benefit health care staff in assessing their quality of care. The results show that P-CAT can be a useful tool for management of improving quality of healthcare in terms of person-centred care in the Chinese health care context.

Acknowledgements

We would like to thank Ms Huang Jingjing and Ms Wu Chao, two postgraduates of the School of Public Health, Kunming Medical University, who helped us with the data collection and Dr. Magnus Persson, Lund University for proofreading the manuscript.

Contributors

LC was responsible for the study design, data analysis, and drafting the paper. GA , PT and KM contributed to the study design and provided comments on the paper during the writing process. DE and LB provided comments on the paper during the writing process. HF, JZ and JY were responsible for the data collection

Competing interests

The authors declare that there are no competing interests.

Funding

For peer review only - http://bmjopen.bmj.com/site/about/guidelines.xhtml

The study was supported by grants from the National Natural Science Fund of China (Grant number: 81611130077) and the Swedish Research Council (Grant number: 2015-06243).

Ethical approval and consent to participate

This study has been approved by the Ethics Committee of Kunming Medical University. Oral and written informed consent was obtained from all persons participating in the study.

Data sharing statement

The datasets used and/or analysed during the current study is available from the corresponding author on reasonable request.

References

1. Pefoyo AJK, Bronskill SE, Gruneir A, et al. The increasing burden and complexity of multimorbidity. *Bmc Public Health* 2015;15 doi: UNSP 415
10.1186/s12889-015-1733-2
2. World population prospects: the 2012 revision. In: Department of Economic and Social Affairs (DoEaSA) PD, ed. New York: United Nations, 2013.
3. Report of the fifth National Health Services survey in China. In: Information CfHSa, ed. Beijing: China Statistics Press, 2014.
4. Liao CC, Li CR, Lee SH, et al. Social support and mortality among the aged people with major diseases or ADL disabilities in Taiwan: A national study. *Arch Gerontol Geriat* 2015;60(2):317-21. doi: 10.1016/j.archger.2014.11.007
5. Nolan MR, Davies S, Brown J, et al. Beyond 'person-centred' care: a new vision for gerontological nursing. *J Clin Nurs* 2004;13(3a):45-53. doi: DOI 10.1111/j.1365-2702.2004.00926.x
6. Edvardsson D, Fetherstonhaugh D, McAuliffe L, et al. Job satisfaction amongst aged care staff: exploring the influence of person-centered care provision. *Int Psychogeriatr* 2011;23(8):1205-12. doi: 10.1017/S1041610211000159
7. McCormack B, Dewing J, Breslin L, et al. Developing person-centred practice: nursing outcomes arising from changes to the care environment in residential settings for older people. *Int J Older People Nurs* 2010;5(2):93-107. doi: 10.1111/j.1748-3743.2010.00216.x [published Online First: 2010/10/12]
8. Edvardsson D, Sandman PO, Rasmussen B. Construction and psychometric evaluation of the Swedish language Person-centred Climate Questionnaire - staff version. *J Nurs Manag* 2009;17(7):790-5. doi: 10.1111/j.1365-2834.2009.01005.x [published Online First: 2009/10/02]
9. Edvardsson D, Fetherstonhaugh D, Nay R, et al. Development and initial testing of the Person-centered Care Assessment Tool (P-CAT). *Int Psychogeriatr* 2010;22(1):101-08. doi: 10.1017/S1041610209990688
10. White DL, Newton-Curtis L, Lyons KS. Development and initial testing of a measure of person-directed care. *Gerontologist* 2008;48 Spec No 1:114-23. [published Online First: 2008/09/20]
11. Chappell NL, Reid CR, Gish JA. Staff-based measures of individualized care for persons with dementia in long-term care facilities. *Dementia* 2007;6(4):527-47. doi: 10.1177/1471301207084372
12. Zimmerman S, Allen J, Cohen LW, et al. A Measure of Person-Centered Practices in Assisted Living: The PC-PAL. *Journal of the American Medical Directors Association* 2015;16(2):132-37. doi: 10.1016/j.jamda.2014.07.016

For peer review only - http://bmjopen.bmj.com/site/about/guidelines.xhtml

13. Selan D, Jakobsson U, Condelius A. The Swedish P-CAT: modification and exploration of psychometric properties of two different versions. *Scand J Caring Sci* 2017;31(3):527-35. doi: 10.1111/scs.12366
14. Sjogren K, Lindkvist M, Sandman PO, et al. Psychometric evaluation of the Swedish version of the Person-Centered Care Assessment Tool (P-CAT). *Int Psychogeriatr* 2012;24(3):406-15. doi: 10.1017/S104161021100202x
15. Tak YR, Woo HY, You SY, et al. [Validity and Reliability of the Person-centered Care Assessment Tool in Long-term Care Facilities in Korea]. *J Korean Acad Nurs* 2015;45(3):412-9. doi: 10.4040/jkan.2015.45.3.412 [published Online First: 2015/07/15]
16. Martinez T, Suarez-Alvarez J, Yanguas J, et al. Spanish validation of the Person-centered Care Assessment Tool (P-CAT). *Aging Ment Health* 2016;20(5):550-58. doi: 10.1080/13607863.2015.1023768
17. Rokstad AMM, Engedal K, Edvardsson D, et al. Psychometric evaluation of the Norwegian version of the Person-centred Care Assessment Tool. *Int J Nurs Pract* 2012;18(1):99-105. doi: 10.1111/j.1440-172X.2011.01998.x
18. Zhong XB, Lou VWQ. Person-centered care in Chinese residential care facilities: a preliminary measure. *Aging Ment Health* 2013;17(8):952-58. doi: 10.1080/13607863.2013.790925
19. Edvardsson D, Koch S, Nay R. Psychometric evaluation of the English language Person-centred Climate Questionnaire - staff version. *J Nurs Manage* 2010;18(1):54-60. doi: 10.1111/j.1365-2834.2009.01038.x
20. Guillemin F. Cross-Cultural Adaptation and Validation of Health-Status Measures. *Scand J Rheumatol* 1995;24(2):61-63. doi: Doi 10.3109/03009749509099285
21. Beaton DE, Bombardier C, Guillemin F, et al. Guidelines for the process of cross-cultural adaptation of self-report measures. *Spine* 2000;25(24):3186-91. doi: Doi 10.1097/00007632-200012150-00014
22. Reise SP, Waller NG, Comrey AL. Factor analysis and scale revision. *Psychol Assessment* 2000;12(3):287-97. doi: 10.1037//1040-3590.12.3.287
23. Streiner DL, Norman GR. Health measurement scales : a practical guide to their development and use: Oxford : Oxford University Press, 2008
4. ed. 2008.
24. Pett MA, Lackey NR, Sullivan JJ. Making sense of factor analysis : the use of factor analysis for instrument development in health care research: London : SAGE, cop. 2003 2003.
25. Bland JM, Altman DG. Cronbach's alpha. *Brit Med J* 1997;314(7080):572-72. doi: DOI 10.1136/bmj.314.7080.572
26. Landis JR, Koch GG. The measurement of observer agreement for categorical data. *Biometrics* 1977;33(1):159-74. [published Online First: 1977/03/01]

For peer review only

For peer review only - http://bmjopen.bmj.com/site/about/guidelines.xhtml

Psychometric evaluation of the Chinese version of the Person-centred Care Assessment Tool

Journal:	*BMJ Open*
Manuscript ID	bmjopen-2019-031580.R1
Article Type:	Original research
Date Submitted by the Author:	05-Dec-2019
Complete List of Authors:	Le, Cai; Kunming Medical University in Kunming,<br>Ma, Ke; The third people's hospital of Kunming, Palliative Care<br>Tang, Pingfen; Kunming Medical University<br>Edvardsson, David; La Trobe University School of Nursing and Midwifery<br>Behm, Lina; Kristianstad University College, Faculty of Health Sciences<br>Zhang, Jie; the third people's hospital of Kunming, palliative care<br>Yang, Jiqun; the third people's hospital of Kunming<br>Fu, Haiyan; the third people's hospital of Kunming<br>Ahlström, Gerd; Lunds Universitet, Health Sciences
**Primary Subject Heading**:	Geriatric medicine
Secondary Subject Heading:	Palliative care, Nursing, Evidence based practice
Keywords:	Person-centered care, older people, PALLIATIVE CARE, instrument evaluation, factor analysis

SCHOLARONE™ Manuscripts

I, the Submitting Author has the right to grant and does grant on behalf of all authors of the Work (as defined in the below author licence), an exclusive licence and/or a non-exclusive licence for contributions from authors who are: i) UK Crown employees; ii) where BMJ has agreed a CC-BY licence shall apply, and/or iii) in accordance with the terms applicable for US Federal Government officers or employees acting as part of their official duties; on a worldwide, perpetual, irrevocable, royalty-free basis to BMJ Publishing Group Ltd ("BMJ") its licensees and where the relevant Journal is co-owned by BMJ to the co-owners of the Journal, to publish the Work in this journal and any other BMJ products and to exploit all rights, as set out in our licence.

The Submitting Author accepts and understands that any supply made under these terms is made by BMJ to the Submitting Author unless you are acting as an employee on behalf of your employer or a postgraduate student of an affiliated institution which is paying any applicable article publishing charge ("APC") for Open Access articles. Where the Submitting Author wishes to make the Work available on an Open Access basis (and intends to pay the relevant APC), the terms of reuse of such Open Access shall be governed by a Creative Commons licence – details of these licences and which Creative Commons licence will apply to this Work are set out in our licence referred to above.

Other than as permitted in any relevant BMJ Author's Self Archiving Policies, I confirm this Work has not been accepted for publication elsewhere, is not being considered for publication elsewhere and does not duplicate material already published. I confirm all authors consent to publication of this Work and authorise the granting of this licence.

For peer review only

Psychometric evaluation of the Chinese version of the Person-centred Care Assessment Tool

Running title: Evaluation of the Chinese version of P-CAT

Le Cai, PhD[1], Ke Ma, MD[2], Pingfen Tang, RN[1], David Edvardsson, PhD[3], Lina Behm, PhD[4], Jie Zhang, MD[5] , Jiqun Yang, MD[6], Haiyan Fu, MD[5] and Gerd Ahlström, PhD[7]*

* Corresponding author: Professor Gerd Ahlström, Department of Health Sciences, Faculty of Medicine, Lund University, Box 157, SE-221 00 Sweden. Email: gerd.ahlstrom@med.lu.se Phone: +46 46 702 747767

[1] PhD, Professor, School of Public Health, Kunming Medical University, Cheng Gong New City, Kunming, China

[2] Affiliated physician, Division of palliative care, the Third People's Hospital of Kunming, China

[3] Professor, Austin Health/Northern Health Clinical Schools of Nursing College of Science, Health and Engineering, La Trobe University, Australia

[4] PhD, Senior Lecturer, Department of nursing educations and integrated health science, Kristianstad University, Sweden

[5] Physician, Division of palliative care, the Third People's Hospital of Kunming, China

[6] Vice-Director, Division of palliative care, the Third People's Hospital of Kunming, China

[7] PhD, Professor, Department of Health Sciences, Faculty of Medicine, Lund University, Sweden. ORCID no 0000-0001-6230-7583

Wordcount: 3837 words

For peer review only - http://bmjopen.bmj.com/site/about/guidelines.xhtml

Abstract

Objective: This study aims to test a Chinese cross-cultural adaptation of the English version of the P-CAT and evaluate its psychometric properties.

Design: P-CAT was translated/back-translated using established procedures. Psychometric evaluation of the Chinese version of the Person-centred Care Assessment Tool.

Setting: Two hospitals covering urban and suburban areas of Kunming, in the Yunnan province of China.

Participants: 152 hospital staff completed the survey.

Main outcome measure(s): Construct validity and reliability, including internal consistency and test-retest reliability, were assessed among a sample of hospital staff.

Results: The factor analysis resulted in a two-component solution that consisted of two sub-scales. The corrected item-total correlations for all of the items ranged from 0.14 to 0.44, with six items that did not meet the cut-off level for item-total correlation (>0.3). The Chinese P-CAT demonstrated strong reliability, with a Cronbach's alpha of 0.91-0.94 for the scales, and a test-retest reliability coefficient of 0.88 for the overall scale scores. The ICC was 0.92 (95 % CI: 0.90- 0.95).

Conclusion: P-CAT appears to be a promising measure for evaluating staff perceptions of the person-centredness in Chinese hospital environments. The results show that P-CAT can be a useful tool for improving the quality of healthcare in terms of person-centred care in the Chinese context.

Keywords: Person-centered care, older people, palliative care, instrument evaluation, factor analysis

For peer review only

Article summary

Strengths and limitations of this study

- The first study to validate a translated and culturally adapted but otherwise unmodified English version of P-CAT in a Chinese context.
- High response rate (92.1%).
- Convenience sampling method may limit the ability to generalize the results.
- The Chinese P-CAT has been tested only in this geriatric hospital setting.

For peer review only

For peer review only - http://bmjopen.bmj.com/site/about/guidelines.xhtml

Introduction

Population ageing is occurring globally, and multi-morbidity is highly prevalent, particularly in older people, who are generally frequent healthcare users.[1] The Chinese population is one of the fastest-ageing populations in the world, and the number of people aged 60 years and older is expected to rise from 193 million (13.9%) in 2013 to 454 million (32.8%) in 2050.[2] This rapid increase in the number of older people in China is reflected in the increasing number of older people admitted to hospitals. The National Health Services survey of China shows that the hospitalization rate of people aged 65 years and older increased significantly from 6.1% to 19.9% during the period 2003–2013,[3] and chronic diseases occur more frequently in older people and are more difficult to cure when multiple diseases coexist.[4] Thus, the healthcare system in China is facing great challenges due to the aging population who require high-quality care from a wide range of multiple care professionals.

Person-centred care (PCC) is now regarded as a valuable approach to use when it comes to improving long-term care for older people,[5] and the quality of care is often described in terms of its being person-centred.[6-8] The growing interest in applying PCC is accompanied by an interest in developing instruments, commonly questionnaires answered by staff as a proxy for the ill person.[7,9] The core values of PCC are the social relationship between the staff and the ill person and the close family, characterized by respect for the person's will, mutuality, openness, honesty, and sympathetic presence.[8] Even though there is a range of terms in the literature for describing PCC, all imply a similar philosophy of looking at the person's needs instead of a disease.[7] A review shows that person-centred interventions are multifactorial and that the complexity of the interventions can explain the range of existing outcome variables.[10]

To date, a number of instruments have been developed to assess the PCC in residential care facilities for older people, and these instruments are mainly based on the opinions of staff working in such facilities. The instruments are the English Person-centred Climate

For peer review only - http://bmjopen.bmj.com/site/about/guidelines.xhtml

Questionnaire – Staff version (PCQ-S),[11] the Person-centred Care Assessment Tool (P-CAT),[12]the Staff Assessment Person-Directed Care tool (PDC),[13] the Individualized Care tool (IC)[14] and the Staff Person-Centred Practices in Assisted Living tool (Staff PC-PAL).[15] Of these, the tool most commonly used internationally, with established credibility, is the P-CAT.[7,9] The P-CAT questionnaire was designed by Edvardsson and colleagues[12] and involves a caregiver-based self-report assessment scale used to measure the extent to which the staff agree that a number of pre-defined characteristics/processes of person-centred care are in place within their team, unit or nursing home. The P-CAT is easy to use, with only 13 items. It has a process-focus, as evidenced in items connected with work routines, residents' life stories, assessment of personal needs, and discussion concerning best care for the residents. It has been validated and used in English,[12] Swedish,[16-17] Korean,[18] Spanish,[19] Norwegian,[20] and Chinese.[21]

A recently published literature review [22] indicates that the studies of PCC were predominantly on populations in Western Europe and the USA. However, the increasing proportion of older people implies an urgent need for the implementation of PCC for the purpose of improving the quality of care. The study of Zhong and colleagues[21] found that the P-CAT was a good tool to adapt for use in the Chinese context. A 24-item Chinese version of the original English 13-item P-CAT was developed and later reduced to 15 items as a result of psychometric validation on staff. This modified version of P-CAT was changed to such an extent as to make it impossible to compare the results with results obtained by use of the original English version. Two items were removed and four items were added, generating three scales.[21] This Chinese version was validated on staff working in residential care for patients with dementia because P-CAT was originally intended for use as a research instrument to measure the presence of person-centred care in this context.[12,23]

Palliative care is provided in a person-centred environment, where the older person and

For peer review only - http://bmjopen.bmj.com/site/about/guidelines.xhtml

the family feel welcome, feel that they are seen, feel that they are involved by the staff. This type of supportive care has been developed in hospice settings by specialists in the care of patients dying from incurable cancer, and older persons dying from other diseases do not have the same access to palliative care.[24-25] The ageing people with multiple morbidities have complex care needs and multiple symptoms in their last period of life, which requires both geriatric and palliative expertise. However, staff need education about palliative care to be able to provide person-centered care integrated with a focus on symptom management and quality of life for the older people and their family.[24,26] This study is part of an intervention project entitled "Palliative care for elderly people with chronic illnesses: a comparative study between Sweden and China". The purpose of the project is to improve palliative care through work-place education in geriatric hospital care.[27] In order to promote the improvement of the quality of care through the implementation of person-centred palliative care in a Chinese healthcare context, further testing and cross-validation of the original P-CAT was performed on staff in Chinese geriatric palliative hospital settings. A short instrument as P-CAT was preferable in this study due to the need for combinations with additional measurements. It is necessary to ensure that this outcome measure is applicable in the evaluation of educational intervention for staff working in a geriatric hospital. Also, a psychometric evaluation of the original 13 items of the English version means that it should be possible to compare results from Sweden in forthcoming studies within the current intervention project involving Sweden[28] and China.

In China, PCC remains a poorly understood approach that is not generally applied in geriatric services, and one of the possible barriers is the absence of a dependable and valid instrument to measure person-centred care. Therefore, the aim of this study was to accomplish a cultural adaptation of the original English version of the P-CAT for Chinese nurses as well as evaluate the psychometric properties of the newly translated Chinese version applied in geriatric palliative care.

For peer review only - http://bmjopen.bmj.com/site/about/guidelines.xhtml

Methods

This study tested and evaluated the Chinese version of the P-CAT in Mandarin, the most common language in China, on nurses in a hospital context.

The original English instrument

P-CAT measures the extent to which staff engaged in long-term care of the elderly consider their settings to be person-centred. The English P-CAT questionnaire has been validated in an Australian sample of nursing home staff and was shown to have satisfactory psychometric properties.[12] The self-reported questionnaire exists in both a staff version and a version for older persons. In this study, the version for staff was used. It comprises 13 items covering three sub-scales (the Extent of personalizing care, the Amount of organizational support, and the Degree of environmental accessibility). The Extent of personalizing care was measured through items 1-7, Amount of organizational support was measured through items 8-11, and Degree of environmental accessibility was measured through items 12-13. A 5-point Likert-type scale ranging from 1 (strongly disagree) to 5 (strongly agree) was used for scoring. Items 8–12 were negatively worded and the responses were reverse-scored before analysing the data and calculating a total score. Aggregated scores were calculated by means of simple sum scores at the sub-scale and total scale levels. The total score ranged from 13 to 65 and a higher score indicated a higher degree of person-centredness.

Translation and cross-cultural adaptation of the P-CAT

The process of translation and cross-cultural adaptation followed International test commission guidelines.[29,30] Three native Chinese speakers produced independent forward translations from English to Chinese; two had a Public Health degree and the third was a senior nurse who

For peer review only - http://bmjopen.bmj.com/site/about/guidelines.xhtml

had worked in a department of palliative care for over ten years. First, the translators agreed upon a consensus version after discussing the different versions. Then this consensus version was back-translated into English by two new translators who were bilingual and had no knowledge of the procedures of the forward translation. After that, any discrepancies were resolved by an expert committee composed of all the translators, two geriatric physicians, one senior nurse and two university professors. The back-translated version was modified by comparing it with the original version. The expert committee culturally adapted a few phrases to fit the Chinese setting, resulting in a pre-final version. The final version of the Chinese P-CAT was generated after pre-testing for face-validity, involving ten female nurses from a municipal hospital in Kunming, with a mean age of 31.2 years (SD ±8.3). The pre-testing resulted in no changes. The ten staff members who participated in the face-validity testing of the pre-final version did not participate in the later study.

Sample, participants and data collection

The sample was selected to cover hospital nurses working both in urban and suburban areas of Kunming, the capital of the Yunnan province in southwest China. Two municipal hospitals in Kunming were chosen through a convenience sampling method: one hospital from the Wu Hua district (an urban area of Kunming) and one from the Guan Du district (a suburban area of Kunming).

Patients who were admitted to the sampled hospitals had multiple, complex, and overlapping chronic diseases, and most patients were aged 60 years or older. The following criterion for inclusion was used: municipal level hospital with a department of palliative care. All nurses (n=165) on duty (all shifts on the day of the data collection) at the palliative care departments of the two hospitals were deemed to be eligible for participation and were asked whether they would be willing to participate in the study. The sample size of the study was

For peer review only - http://bmjopen.bmj.com/site/about/guidelines.xhtml

determined by the criteria suggested by Reise et al.,[31] where the intra-class correlation (ICC) >0.8 and five to ten persons for each item of the scale and sample sizes of 100 are often adequate.

The questionnaires were distributed by two graduate students to all participants, and the completed questionnaires were then anonymously collected on site. In addition to the P-CAT scale, demographic characteristics of the participants were also collected by means of a questionnaire that assessed age, gender, level of education, ethnicity and position. Finally, 152 nurses gave their informed consent and completed the Chinese version of P-CAT questionnaire. This gives an overall response rate of 92.1 %. The 152 participants were asked to take part in both the test and retest assessments. The retest was conducted between one and two weeks after the initial test.

Psychometric evaluation

Only one item in one questionnaire had a missing value and it was replaced with the mean value of the item for the entire group.[32] An exploratory factor analysis (EFA) with extraction of principal components by both varimax and oblique orthogonal rotation was used to evaluate construct validity. After either direct varimax orthogonal rotating or oblique orthogonal rotating, components in the structural matrix comprised the same numbers of items and loadings, indicating that no difference was found between these two methods in the analysis, and thus only the results of the analysis with varimax orthogonal rotation are presented. Bartlett's test of sphericity and the Keiser-Meyer-Olkin (KMO) measure were applied in order to check the appropriateness of the factor analysis. In order to assess whether the correlation between items was adequate based on a criterion of $p< 0.0001$, Bartlett'stest of sphericity was used, and a criterion of KMO $\geqslant$0.70 was used to indicate sample adequacy. When the Kaiser criterion of eigenvalues was $\geqslant 1$, factors were extracted. A cut-off of 0.50 was applied to

For peer review only - http://bmjopen.bmj.com/site/about/guidelines.xhtml

determine item loadings on each individual component.[33]

The internal consistency and test-retest reliability were evaluated. The internal consistency for the total and sub-scale scores was estimated using Cronbach's alpha coefficient, and Cronbach's alpha ≥0.50 was used as acceptable internal consistency reliability.[34] The item-total correlations were also computed, and the cut-off score for acceptable item-total correlations was set to between 0.3 and 0.8 to ensure moderate correlation and avoid item redundancy.[32] Test-retest reliability was examined with the intra-class correlation (ICC) and Pearson's correlation coefficient (r), where an ICC >0.80 was taken to indicate satisfactory reliability.[35] The paired t-test (2-tailed) was used to verify whether the mean scores from the test and retest differed.

The results of the principal component analysis (PCA) indicate that three factors with eigenvalues above 1 produced a three-component rotated solution that explained 60.2% of the total variance in the data. After direct varimax orthogonal rotating, the structure matrix showed that the first component comprised seven items, the second comprised four items,and the third comprised two items. With regard to the internal consistency reliability, the Cronbach's alpha coefficient of the 13-item Chinese P-CAT was 0.65 for the total scale, 0.67 for the first sub-scale, 0.85 for the second sub-scale and 0.42 for the third sub-scale. Since sub-scale three failed to meet the reliability cut-off of 0.5, the analysis was re-run by forcing the EFA into 2 factors.

The two-factor model was also evaluated by means of a Confirmatory Factor Analysis (CFA) within the framework of Structural Equation Modelling. The goodness of fit was evaluated using indices of the Normed Fit Index (NFI), the Comparative Fit Index (CFI) and the Root Mean Square Error of Approximation (RMSEA). The results indicated that the goodness of fit of the questionnaire was 0.91 for the NFI, 0.91 for the CFI and 0.73 for the RMSEA. Thus, the CFA supported the exploratory findings. Statistical significance was determined based on two-tailed P values of <0.05. All data analyses were performed using SPSS 17.0 software.

For peer review only - http://bmjopen.bmj.com/site/about/guidelines.xhtml

Patient and Public Involvement

Patients, relatives, and staff from palliative care were involved in the development of work-place education for staff and informed by patients' priorities, experience, and preferences.[27] It is planned to disseminate the results to staff at meetings about palliative care.

Results

Demographic characteristics of the study group

As described in Table 1, the sample consisted of only female nurses, with a mean age of 30.7 years (SD ±8.7, range 19-53 years). In total, 25.7% of the nurses had a Bachelor's degree or higher. More than one-fourth of the participants reported a minority ethnicity.

Table 1 Demographic characteristics of the study sample (n=152)

Characteristics	n (%)
Gender	
Female	152 (100.0)
Male	0 (0.0)
Age (years)	
18-30	96 (63.2)
31-39	22 (14.5)
⩾40	34 (22.4)
Level of education	
High school	14 (9.2)
Secondary school	39 (25.7)
Junior college	60 (39.5)
Bachelor	39 (25.7)
Ethnicity	
Han	111 (73.0)

For peer review only - http://bmjopen.bmj.com/site/about/guidelines.xhtml

Minorities	41 (27.0)

Construct validity Bartlett's test of sphericity was significant (754.99, p< 0.0001) and the KMO measure were satisfactory (0.78), which indicates that correlations between items were sufficient to conduct a principal component analysis (PCA). As shown in Table 2, the results of the PCA indicate that two factors with eigenvalues above 1 produced a two-component rotated solution that explained 50.4% of the total variance in the data. After direct varimax orthogonal rotating, the structural matrix showed that the first component comprised nine items (loadings between 0.16 and 0.79) and the second comprised four items (loadings between 0.71 and 0.89). The two sub-scales were labelled as (1) Extent of personalizing care and (2) Amount of organizational and environmental support.

Table 2 Rotated component matrix for PCA of the 2-factor Chinese P-CAT

**Item number**	**Item content**	**Factor loadings**	
		**Sub-scale 1: Extent of personalizing care**	**Sub-scale 2: Amount of organizational and environmental support**
1	We often discuss how to provide person-centred care.	**0.73**	0.008
2	We have formal team meetings to discuss residents' care.	**0.79**	0.045
3	The life history of the residents is formally used in the care plans we use.	**0.76**	0.028

For peer review only - http://bmjopen.bmj.com/site/about/guidelines.xhtml

4	The quality of the interaction between staff and residents is more important than getting the tasks done.	**0.72**	-0.087
5	We are free to alter work routines based on residents' preferences.	**0.67**	0.117
6	Residents are offered the opportunity to be involved in individualized everyday activities.	**0.54**	-0.109
7	Assessment of residents' needs is undertaken on a daily basis.	**0.17**	0.26
8	I simply do not have the time to provide person-centred care.	-0.208	**0.78**
9	The environment feels chaotic.	-0.096	**0.89**
10	We have to get the work done before we can worry about a homelike environment.	**0.64**	-0.364
11	The organization prevents me from providing person-centred care.	-0.094	**0.89**
12	It is hard for residents in the facility to find their way around.	-0.036	**0.71**
13	Residents are able to access outside space as they wish.	**0.35**	-0.087
Total variance explained (%)	50.4 (total 2 sub-scales)	30.18	20.24
Cronbach's alpha	0.92 (total 13 items)	0.94	0.91

Note: Bold number means included in the scale

For peer review only - http://bmjopen.bmj.com/site/about/guidelines.xhtml

Reliability

With regard to the internal consistency reliability, the Cronbach's alpha coefficient of the 13-item Chinese P-CAT was 0.92 for the total scale, 0.94 for the sub-scale Extent of personalized care, and 0.91 for the sub-scale Amount of organizational and environmental support sub-scale(Table 2), indicating that the overall internal consistency reliability was strong. The corrected item-total correlations for all items ranged from 0.14 to 0.44 (Table 3), with six items (items 6, 7, 9, 11, 12 and 13) failing to meet the commonly suggested cut-off level for item-total correlation (>0.3).

Table 3 Item performance and reliability test of the Chinese P-CAT

**Item number**	**Item content**	**Mean±SD**	**Corrected item- total correction**	**Cronbach's alpha if item deleted**
1	We often discuss how to provide person-centred care.	4.19±0.70	0.36	0.62
2	We have formal team meetings to discuss residents' care.	4.32±0.66	0.44	0.61
3	The life history of the residents is formally used in the care plans we use.	4.24±0.73	0.41	0.62
4	The quality of the interaction between staff and residents is more important than getting the tasks done.	4.50±0.65	0.32	0.63
5	We are free to alter work routines based on residents' preferences.	4.20±0.77	0.42	0.61
6	Residents are offered the opportunity to be involved in individualized everyday activities.	3.86±0.82	0.23	0.64

For peer review only

7	Assessment of residents' needs is undertaken on a daily basis.	2.30±1.10	0.19	0.65
8	I simply do not have the time to provide person-centred care.	3.89±0.82	0.35	0.62
9	The environment feels chaotic.	4.11±1.00	0.15	0.65
10	We have to get the work done before we can worry about a homelike environment.	1.74±0.62	0.37	0.62
11	The organization prevents me from providing person-centred care.	4.14±0.92	0.17	0.65
12	It is hard for residents in the facility to find their way around.	2.22±0.88	0.26	0.64
13	Residents are able to access outside space as they wish.	3.89±0.82	0.14	0.65

Table 4 shows the results from the test-retest reliability assessment of the Chinese P-CAT. Theresults of Pearson's correlation coefficient analysis indicated that the Chinese P-CAT had a high correlation between the test and retest on all scale levels: on the sub-scales Extent of personalizing care ($r=0.90$, $P<0.01$) and Amount of organizational and environmental support ($r=0.88$, $P<0.01$) and on the overall scale ($r=0.88$, $P<0.01$). A paired t-test also confirmed that there was no significant difference between the mean scores of the P-CAT on the test and retest ($P>0.05$). The ICC of the total score between the test and retest was 0.92, providing further support for stating that the scale had satisfactory test-retest reliability.

For peer review only - http://bmjopen.bmj.com/site/about/guidelines.xhtml

Table 4 Test-retest reliability of the Chinese P-CAT

Scale dimension	1st test (Mean±SD)	2nd test (Mean±SD)	p	Pearson correlation coefficient (r)	95% CI	ICC (95 % CI)
Extent of personalizing care	33.35±3.59	33.13±3.33	0.09	0.90	(0.83, 0.94)	0.94 (0.92, 0.96)
Amount of organizational and environmental support	14.41±1.72	14.34±1.74	0.34	0.88	(0.76, 0.89)	0.91 (0.87, 0.93)
Overall scale	47.76±3.70	47.46±3.65	0.07	0.88	(0.78, 0.92)	0.92 (0.90, 0.95)

Note: p= two-tailed p-values of paired t-test; ICC: intra-class correlation

Discussion

In this study, the English P-CAT was culturally adapted into a Chinese version. During the translation of the English P-CAT into Chinese, a few minor cultural discrepancies were encountered, and two items of the P-CAT have been modified accordingly. In order to use words closer to the Chinese cultural context and more applicable to a hospital environment, the term 'resident' was replaced by 'patient' and the term 'free' was replaced by 'flexible'. After cross-cultural adaptation, the pre-testing of the Chinese version of the P-CATrevealed that the items were clearly understood.

This Chinese version of the P-CAT showed a different sub-scale structure as compared to the original English scale, i.e. a structure with three sub-scales (Extent of personalizing care, Amount of organizational support and Degree of environmental accessibility) consisting of 13 items. On the other hand it showed the same sub-scale structure as the Norwegian scale, i.e. a structure which had two sub-scales (Extent of personalizing care and Amount of organizational and environmental support). The 13 items were mostly in concordance with the Norwegian

For peer review only - http://bmjopen.bmj.com/site/about/guidelines.xhtml

version whereas only item 10 was connected to the first sub-scale.[20] The 2-factor structure has also been confirmed in the Swedish version of the P-CAT based on a large cross-sectional sample of 1,465 staff members from 195 residential elderly care units[17] and in a more recent study based on 142 staff working in residential elderly care units and 182 staff working at healthcare centres or home care centres and in social services.[16] Moreover, in this study the Chinese P-CAT showed strong internal consistency reliability with a Cronbach's alpha of 0.92 for the total scale, indicating that the Chinese P-CAT it matched well with the characteristics and structure of the Norwegian version. However, the different sub-scale structures of this Chinese version of the P-CAT as compared to the original English scale may reflect a difference in sample characteristics of staff and the cultural context. The test-retest reliability assessed by ICC was excellent for overall Chinese P-CAT score (0.92) and both sub-scales (0.94 and 0.91), and the excellent test-retest reliability of the Chinese P-CAT was further evidenced through a Pearson's correlation coefficient (r) of 0.90 for the total scale, 0.88 for the Extent of personalizing care sub-scale and 0.88 for the Amount of organizational and environmental support sub-scale. The results of Pearson's correlation coefficient (r) for the total scale in the Chinese version were higher than in the English (0.66),[12] Swedish (0.75),[16-17] Norwegian (0.83)[20] and previous Chinese (0.68)[21] versions, which indicates that this Chinese P-CAT may have a stronger test-retest reliability than other-language versions of the P-CAT.

In this Mandarin version of the Chinese P-CAT, six items (6, 7, 9, 11, 12 and 13) did not meet the commonly suggested cut-off level for item-total correlation (>0.3). In the previous Chinese version of the P-CAT,[21] items 7 and 9 were deleted due to the low value of factor loading (<0.3), and four newly developed items were added to the scale.[21] Item 7 in this study also had a lower value of factor loading than is recommended.However, we decided to include this item in the sub-scale 1, in agreement with the original English version, although this item had a slightly higher value of factor loading in the case of factor 2. Because these two Chinese

For peer review only - http://bmjopen.bmj.com/site/about/guidelines.xhtml

versions of the P-CAT were evaluated in different healthcare settings (hospital environments and residential care facilities), there is no firm consensus regarding the psychometric properties of the Chinese P-CAT, and the findings suggest that the Chinese version requires further investigation and analysis with other samples and in other settings.

In contrast to the low response rate of 21% in the development study of the original English version P-CAT,[12] our study showed a high response rate of 92.1%. However, some limitations should be noted. First, the P-CAT is primarily intended for use as a research tool to measure the presence of person-centred care in residential aged care facilities but the present study was conducted only in a hospital context, which may limit the ability to generalize the results to staff working in other healthcare contexts. Further psychometric evaluation of this Chinese P-CAT is needed, with samples from residential aged care facilities. Second, criterion-related validity was not assessed as a gold standard for the measurement of person-centred care in hospitals; however, no such criterion exists because of the lack of previous studies in hospital contexts. Third, the content validity of the P-CAT was only considered along with the procedures of cross-cultural adaptation. Fourth, only female participants were included in this study, for which reason this study may be subject to gender bias.

Palliative care in mainland China has developed slowly since the late 1980s but in recent years profesionals, patients and their families, as well as government, have become increasingly aware of its importance.[36] But even though the provision of palliative care is becoming more and more common in China, the hospice and palliative care services are not meeting the growing demand from the ageing population, and the number of patients with cancer and other life-threatening diseases is increasing.[37] The use of a reliable and valid instrument for the assessment of person-centred care in research and practice can serve not only to enhance the quality of elderly care but also to strengthen the ongoing development of palliative care in China. Palliative care implies a person-centred approach characterized by a

For peer review only - http://bmjopen.bmj.com/site/about/guidelines.xhtml

holistic view of the person and a belief that the person should be supported in living a life with dignity. Palliative care strives to make the whole person visible and prioritizes the patient's spiritual, existential, social, and psychological needs to the same extent as physical needs.[38,39] The patient version of P-CAT is used previously by the research group as a complementary assessment in the evaluation of palliative care interventions, going together with instruments for pain management and symptoms relief for the patients.[40] Another application is in the evaluation of work-place education about palliative care for staff in the nursing home context.[28] In this current project,[27] for instance, it is being used in order to investigate whether the staff's attitude to person-centredness has changed as a result of the education.

Conclusion

The results indicated that the 13-item Chinese P-CAT which is a cross-culturally adapted version of the English P-CAT shows strong internal consistency and test-retest reliability and appears to be a promising measure for evaluating staff perceptions of person-centredness in a Chinese palliative hospital setting. The results of published psychometric evaluations indicate the use of two scales in the P-CAT as was determined in this study. However, we suggest that the measurement properties of the Chinese version should be further evaluated in other samples and in different healthcare settings. An additional issue for future studies is to evaluate this instrument as a summative measurement with cut-off values for different levels of person-centred care. This can benefit healthcare staff in assessing their quality of care. The results show that P-CAT can be a useful tool for improving the quality of healthcare in terms of person-centred care in the Chinese context.

Acknowledgements

We would like to thank Ms Huang Jingjing and Ms Wu Chao, two postgraduates of the

For peer review only - http://bmjopen.bmj.com/site/about/guidelines.xhtml

School of Public Health, Kunming Medical University, who helped us with the data collection, and Dr. Magnus Persson, Lund University for proofreading the manuscript.

Contributors

LC was responsible for the study design, data analysis and drafting of the paper. GA , PT and KM contributed to the study design and provided comments on the paper during the writing process. DE and LB provided comments on the paper during the writing process. HF, JZ and JY were responsible for the data collection. All authors have read and approved the final version of the manuscript.

Competing interests

The authors declare that there are no competing interests.

Funding

The study was supported by grants from the National Natural Science Fund of China (Grant number: 81611130077) and the Swedish Research Council (Grant number: 2015-06243).

Ethical approval and consent to participate

This study has been approved by the Ethics Committee of Kunming Medical University with the ID number KMU01. Oral and written informed consent was obtained from all persons participating in the study.

Data sharing statement

The datasets used and/or analysed during the current study are available from the corresponding author on reasonable request.

For peer review only - http://bmjopen.bmj.com/site/about/guidelines.xhtml

For peer review only

For peer review only - http://bmjopen.bmj.com/site/about/guidelines.xhtml

References

1. Pefoyo AJK, Bronskill SE, Gruneir A, et al. The increasing burden and complexity of multimorbidity. *BMC Public Health* 2015;15:415. doi:10.1186/s12889-015-1733-2
2. World population prospects: the 2012 revision. In: Department of Economic and Social Affairs (DoEaSA) PD, ed. New York: United Nations, 2013.
3. Xu J, Chen T, Liu Y, et al. Easily manageable prognostic factors in 152 Chinese elderly acute myeloid leukemia patients: A single-center retrospective study. *J Biomed Res* 2014;28(5):396-405. doi:10.7555/JBR.28.20130164
4. Liao CC, Li CR, Lee SH, et al. Social support and mortality among the aged people with major diseases or ADL disabilities in Taiwan: A national study. *Arch Gerontol Geriatr* 2015;60(2):317-21. doi: 10.1016/j.archger.2014.11.007
5. Nolan MR, Davies S, Brown J, et al. Beyond 'person-centred' care: a new vision for gerontological nursing. *J Clin Nurs* 2004;13(3a):45-53. doi: DOI 10.1111/j.1365-2702.2004.00926.x
6. Edvardsson D, Watt E, Pearce F. Patient experiences of caring and person-centredness are associated with perceived nursing care quality. *J Adv Nurs* 2017;73(1),217–227.doi:10.1111/jan.13105
7. Edvardsson D, Innes A. Measuring Person-centered Care: A Critical Comparative Review of Published Tools. *Gerontologist* 2010;50(6),834-846.doi:10.1093/geront/gnq047
8. McCormack B. A conceptual framework for person-centred practice with older people. *Int J Nurs Pract* 2003;9:202–209. DOI:10.1046/j.1440-172x.2003.00423.x
9. Martinez T, Suárez-Alvarez J, Yanguas J. Instruments for assessing Person Centered Care in Gerontology. *Psicothema* 2016;28(2):114-121.doi:10.7334/psicothema2015.263
10. Brownie S, Nancarrow S. Effects of person-centered care on residents and staff in aged-care facilities: a systematic review. *Clin Interv Aging* 2013;8:1–10.doi:10.2147/CIA.S38589
11. Edvardsson D, Sandman PO, Rasmussen B. Construction and psychometric evaluation of the Swedish language Person-centred Climate Questionnaire - staff version. *J Nurs Manag* 2009;17(7):790-5.doi: 10.1111/j.1365-2834.2009.01005.x
12. Edvardsson D, Fetherstonhaugh D, Nay R, et al. Development and initial testing of the Person-centered Care Assessment Tool (P-CAT). *Int Psychogeriatr* 2010;22(1):101-08. doi:10.1017/S1041610209990688
13. White DL, Newton-Curtis L, Lyons KS. Development and initial testing of a measure of person-directed care. *Gerontologist* 2008;48.DOI:10.1093/geront/48.supplement_1.114-123
14. Chappell NL, Colin Reid R, Gish JA. Staff-based measures of individualized care for persons with dementia in long-term care facilities. *Dementia* 2007;6(4):527-47. doi:10.1177/1471301207084372
15. Zimmerman S, Allen J, Cohen LW, et al. A Measure of Person-Centered Practices in Assisted Living: The PC-PAL. *J Am Med Dir Assoc* 2015;16(2):132-137. doi:10.1016/j.jamda.2014.07.016
16. Selan D, Jakobsson U, Condelius A. The Swedish P-CAT: modification and exploration of psychometric properties of two different versions. *Scand J Caring Sci* 2017;31(3):527-35.doi: 10.1111/scs.12366
17. Sjogren K, Lindkvist M, Sandman PO, et al. Psychometric evaluation of the Swedish version of the Person-Centered Care Assessment Tool (P-CAT). *Int Psychogeriatr* 2012;24(3):406-415.doi: 10.1017/S104161021100202x
18. Tak YR, Woo HY, You SY, et al. [Validity and Reliability of the Person-centered Care Assessment Tool in Long-term Care Facilities in Korea]. *J Korean Acad Nurs* 2015;45(3):412-419.doi: 10.4040/jkan.2015.45.3.412
19. Martinez T, Suarez-Alvarez J, Yanguas J, et al. Spanish validation of the Person-centered Care Assessment Tool (P-CAT). *Aging Ment Health* 2016;20(5):550-58.doi: 10.1080/13607863.2015.1023768
20. Rokstad AMM, Engedal K, Edvardsson D, et al. Psychometric evaluation of the Norwegian version of the Person-centred Care Assessment Tool. *Int J Nurs Pract* 2012;18(1):99-105.doi: 10.1111/j.1440-172X.2011.01998.x
21. Zhong XB, Lou VW. Person-centered care in Chinese residential care facilities: a preliminary

For peer review only - http://bmjopen.bmj.com/site/about/guidelines.xhtml

measure. *Aging Ment Health* 2013;17(8):952-58.doi:10.1080/13607863.2013.790925

22. Wang J, Wu B, Bowers BJ, et al. Person-centered dementia care in China: A bilingual literature review. *Gerontol Geriatr Med* 2019;5:1-11.doi:10.1177/2333721419844349.
23. Mao P, Xiao D, Zhang M, et al. Person-centered dementia care in eldercare institutions in Hunan province. *Journal of Nursing Science* 2016;31(21):1-4.
24. Hall S, Petkova H, Tsouros A, et al. Palliative care for older people: better practices. Copenhagen: WHO Regional Office for Europe; 2011. (Accessed October 26, 2019). http://www.euro.who.int/__data/assets/pdf_file/0017/143153/e95052.pdf
25. Davies E, Higginson IJ. Better palliative care for older people. WHO Regional Office for Europe; 2004. (Accessed October 26, 2019). http://www.euro.who.int/__data/assets/pdf_file/0009/98235/E82933.pdf
26. Price DM, Strodtman LK, Montagnini M, et al. Health professionals perceived concerns and challenges in providing palliative and end-of-life care: A qualitative analysis. *Am J Hosp Palliat Care* 2019 Apr;36(4):308-315.doi:10.1177/1049909118812193. Epub 2018 Nov 21.
27. Ahlström G, Benzein E. Themed meetings about palliative care within health care (Additional file 1, The Booklet). In: Bökberg C, Behm L, Wallerstedt B & Ahlström G, editors. Evaluation of person-centeredness in nursing homes after a palliative care intervention: pre and post-test experimental design. *BMC Palliat Care* 2019;18(1):44. doi:10.1186/s12904-019-0431-8.
28. Bökberg C, Behm L, Wallerstedt B, et al. Evaluation of person-centeredness in nursing homes after a palliative care intervention: pre- and post-test experimental design. *BMC Palliat Care* 2019; 18:44. https://doi.org/10.1186/s12904-019-0431-8.
29. Guillemin F. Cross-Cultural Adaptation and Validation of Health-Status Measures. *Scand J Rheumatol* 1995;24(2):61-63. doi: Doi 10.3109/03009749509099285
30. Beaton DE, Bombardier C, Guillemin F, et al. Guidelines for the process of cross-cultural adaptation of self-report measures. *Spine* 2000;25(24):3186-91. doi: Doi 10.1097/00007632-200012150-00014
31. Reise SP, Waller NG, Comrey AL. Factor analysis and scale revision. *Psychol Assessment* 2000;12(3):287-97. doi: 10.1037//1040-3590.12.3.287
32. Streiner DL, Norman GR, Cairney J. Health Measurement Scales: A Practical Guide to Their Development and Use. Fifth edition ed. 2014, Oxford: Oxford University Press.
33. Pett MA, Lackey NR, Sullivan JJ. Making sense of factor analysis : the use of factor analysis for instrument development in health care research: London : SAGE, cop. 2003.
34. Bland JM, Altman DG. Cronbach's alpha. *Brit Med J* 1997;314(7080):572-72. doi: DOI 10.1136/bmj.314.7080.572
35. Landis JR, Koch GG. The measurement of observer agreement for categorical data. *Biometrics* 1977;33(1):159-74. [published Online First: 1977/03/01]
36. Ning XH. Hospice and palliative care in mainland China: History, Current status and challenges. *Chin Med Sci J* 2018; 33(4): 199-203. doi: 10.24920/003524.
37. Lu Y, Gu Y, Yu W. Hospice and palliative care in China: Development and challenges. *Asia Pac J Oncol Nurs* 2018;5:26-32.
38. McCormack B, McCance TV. Development of a framework for personcentred nursing. *J Adv Nurs* 2006. https://doi.org/10.1111/j.1365-2648.2006.04042.x.
39. McCormack B, Dewing J, Breslin L, et al. Developing person-centred practice: nursing outcomes arising from changes to the care environment in residential settings for older people. *Int J Older people Nurs* 2010;5(2):93-107.doi:10.1111/j.1748-3743.2010.00216.x [published Online First: 2010/10/12]
40. Bökberg C, Behm L, Wallerstedt B, et al. Evaluating person-centredness for frail older persons in nursing homes before and after implementing a palliative care intervention. *Nursing Open* 2019;00:1–10. DOI: 10.1002/nop2.408

For peer review only - http://bmjopen.bmj.com/site/about/guidelines.xhtml

BMJ Open

Psychometric evaluation of the Chinese version of the Person-centred Care Assessment Tool

Journal:	*BMJ Open*
Manuscript ID	bmjopen-2019-031580.R2
Article Type:	Original research
Date Submitted by the Author:	23-Jan-2020
Complete List of Authors:	Le, Cai; Kunming Medical University in Kunming,<br>Ma, Ke; The third people's hospital of Kunming, Palliative Care<br>Tang, Pingfen; Kunming Medical University<br>Edvardsson, David; La Trobe University School of Nursing and Midwifery<br>Behm, Lina; Kristianstad University College, Faculty of Health Sciences<br>Zhang, Jie; the third people's hospital of Kunming, palliative care<br>Yang, Jiqun; the third people's hospital of Kunming<br>Fu, Haiyan; the third people's hospital of Kunming<br>Ahlström, Gerd; Lunds Universitet, Health Sciences
**Primary Subject Heading**:	Geriatric medicine
Secondary Subject Heading:	Palliative care, Nursing, Evidence based practice
Keywords:	Person-centered care, older people, PALLIATIVE CARE, instrument evaluation, factor analysis

SCHOLARONE™
Manuscripts

I, the Submitting Author has the right to grant and does grant on behalf of all authors of the Work (as defined in the below author licence), an exclusive licence and/or a non-exclusive licence for contributions from authors who are: i) UK Crown employees; ii) where BMJ has agreed a CC-BY licence shall apply, and/or iii) in accordance with the terms applicable for US Federal Government officers or employees acting as part of their official duties; on a worldwide, perpetual, irrevocable, royalty-free basis to BMJ Publishing Group Ltd ("BMJ") its licensees and where the relevant Journal is co-owned by BMJ to the co-owners of the Journal, to publish the Work in this journal and any other BMJ products and to exploit all rights, as set out in our licence.

The Submitting Author accepts and understands that any supply made under these terms is made by BMJ to the Submitting Author unless you are acting as an employee on behalf of your employer or a postgraduate student of an affiliated institution which is paying any applicable article publishing charge ("APC") for Open Access articles. Where the Submitting Author wishes to make the Work available on an Open Access basis (and intends to pay the relevant APC), the terms of reuse of such Open Access shall be governed by a Creative Commons licence – details of these licences and which Creative Commons licence will apply to this Work are set out in our licence referred to above.

Other than as permitted in any relevant BMJ Author's Self Archiving Policies, I confirm this Work has not been accepted for publication elsewhere, is not being considered for publication elsewhere and does not duplicate material already published. I confirm all authors consent to publication of this Work and authorise the granting of this licence.

For peer review only

Psychometric evaluation of the Chinese version of the Person-centred Care Assessment Tool

Running title: Evaluation of the Chinese version of P-CAT

Le Cai, PhD[1], Ke Ma, MD[2], Pingfen Tang, RN[1], David Edvardsson, PhD[3], Lina Behm, PhD[4], Jie Zhang, MD[5], Jiqun Yang, MD[6], Haiyan Fu, MD[5] and Gerd Ahlström, PhD[7]*

* Corresponding author: Professor Gerd Ahlström, Department of Health Sciences, Faculty of Medicine, Lund University, Box 157, SE-221 00 Sweden. Email: gerd.ahlstrom@med.lu.se
Phone: +46 46 702 747767

[1] PhD, Professor, School of Public Health, Kunming Medical University, Cheng Gong New City, Kunming, China

[2] Affiliated physician, Division of palliative care, the Third People's Hospital of Kunming, China

[3] Professor, Austin Health/Northern Health Clinical Schools of Nursing College of Science, Health and Engineering, La Trobe University, Australia

[4] PhD, Senior Lecturer, Department of nursing educations and integrated health science, Kristianstad University, Sweden

[5] Physician, Division of palliative care, the Third People's Hospital of Kunming, China

[6] Vice-Director, Division of palliative care, the Third People's Hospital of Kunming, China

[7] PhD, Professor, Department of Health Sciences, Faculty of Medicine, Lund University, Sweden. ORCID no 0000-0001-6230-7583

Wordcount: 4234 words (without abstract and tables)

For peer review only - http://bmjopen.bmj.com/site/about/guidelines.xhtml

Abstract

Objective: This study aims to test a Chinese cross-cultural adaptation of the English version of the P-CAT and evaluate its psychometric properties.

Design: P-CAT was translated/back-translated using established procedures. Psychometric evaluation of the Chinese version of the Person-centred Care Assessment Tool.

Setting: Two hospitals covering urban and suburban areas of Kunming, in the Yunnan province of China.

Participants: 152 female hospital staff completed the survey

Main outcome measure(s): Construct validity and reliability, including internal consistency and test-retest reliability, were assessed among a sample of hospital staff.

Results: The factor analysis resulted in a two-component solution that consisted of two sub-scales. The corrected item-total correlations for all of the items ranged from 0.14 to 0.44, with six items that did not meet the cut-off level for item-total correlation (>0.3). The Chinese P-CAT demonstrated strong reliability, with a Cronbach's alpha of 0.91-0.94 for the scales, and a test-retest reliability coefficient of 0.88 for the overall scale scores. The ICC was 0.92 (95 % CI: 0.90- 0.95).

Conclusion: P-CAT appears to be a promising measure for evaluating staff perceptions of the person-centredness in Chinese hospital environments. The results show that P-CAT can be a useful tool for improving the quality of healthcare in terms of person-centred care in the Chinese context.

Keywords: Person-centered care, older people, palliative care, instrument evaluation, factor analysis

For peer review only - http://bmjopen.bmj.com/site/about/guidelines.xhtml

Article summary

Strengths and limitations of this study

- A previous study found the need for revision of the original P-CAT questionnaire. This study validates a translated and culturally adapted but otherwise unmodified English version of P-CAT in a Chinese context.
- High response rate (92.1%).
- Convenience sampling method may limit the ability to generalize the results.
- The Chinese P-CAT has been tested only in this geriatric hospital setting.

For peer review only - http://bmjopen.bmj.com/site/about/guidelines.xhtml

Introduction

Population ageing is occurring globally, and multi-morbidity is highly prevalent, particularly in older people, who are generally frequent healthcare users.[1] The Chinese population is one of the fastest-ageing populations in the world, and the number of people aged 60 years and older is expected to rise from 193 million (13.9%) in 2013 to 454 million (32.8%) in 2050.[2] This rapid increase in the number of older people in China is reflected in the increasing number of older people admitted to hospitals. The National Health Services survey of China shows that the hospitalization rate of people aged 65 years and older increased significantly from 6.1% to 19.9% during the period 2003–2013,[3] and chronic diseases occur more frequently in older people and are more difficult to cure when multiple diseases coexist.[4] Thus, the healthcare system in China is facing great challenges due to the aging population who require high-quality care from a wide range of multiple care professionals.

Person-centred care (PCC) is now regarded as a valuable approach to use when it comes to improving long-term care for older people,[5] and the quality of care is often described in terms of its being person-centred.[6-8] The growing interest in applying PCC is accompanied by an interest in developing instruments, commonly questionnaires answered by staff as a proxy for the ill person.[7,9] The core values of PCC are the social relationship between the staff and the ill person and the close family, characterized by respect for the person's will, mutuality, openness, honesty, and sympathetic presence.[8] Even though there is a range of terms in the literature for describing PCC, all imply a similar philosophy of looking at the person's needs instead of a disease.[7] A review shows that person-centred interventions are multifactorial and that the complexity of the interventions can explain the range of existing outcome variables.[10]

To date, a number of instruments have been developed to assess the PCC in residential care facilities for older people, and these instruments are mainly based on the opinions of staff working in such facilities. The instruments are the English Person-centred Climate

For peer review only - http://bmjopen.bmj.com/site/about/guidelines.xhtml

Questionnaire – Staff version (PCQ-S),[11] the Person-centred Care Assessment Tool (P-CAT),[12] the Staff Assessment Person-Directed Care tool (PDC),[13] the Individualized Care tool (IC)[14] and the Staff Person-Centred Practices in Assisted Living tool (Staff PC-PAL).[15] Of these, the tool most commonly used internationally, with established credibility, is the P-CAT.[7,9] The P-CAT questionnaire was designed by Edvardsson and colleagues[12] and involves a caregiver-based self-report assessment scale used to measure the extent to which the staff agree that a number of pre-defined characteristics/processes of PCC are in place within their team, unit or nursing home. The P-CAT is easy to use, with only 13 items. It has a process-focus, as evidenced in items connected with work routines, residents' life stories, assessment of personal needs, and discussion concerning best care for the residents. It has been validated and used in English,[12] Swedish,[16-17] Korean,[18] Spanish,[19] Norwegian,[20] and Chinese.[21]

A recently published literature review [22] indicates that the studies of PCC were predominantly on populations in Western Europe and the USA. However, the increasing proportion of older people implies an urgent need for the implementation of PCC for the purpose of improving the quality of care. The study of Zhong and colleagues[21] found that the P-CAT was a good tool to adapt for use in the Chinese context. A 24-item Chinese version of the original English 13-item P-CAT was developed and later reduced to 15 items as a result of psychometric validation on staff. This modified version of P-CAT was changed to such an extent as to make it impossible to compare the results with results obtained by use of the original English version. Two items were removed and four items were added, generating three scales.[21] This Chinese version was validated on staff working in residential care for patients with dementia because P-CAT was originally intended for use as a research instrument to measure the presence of PCC in this context.[12,23]

Palliative care is provided in a person-centred environment, where the older person and the family feel welcome, feel that they are seen, feel that they are involved by the staff. This

For peer review only - http://bmjopen.bmj.com/site/about/guidelines.xhtml

type of supportive care has been developed in hospice settings by specialists in the care of patients dying from incurable cancer, and older persons dying from other diseases do not have the same access to palliative care.[24-25] The ageing people with multiple morbidities have complex care needs and multiple symptoms in their last period of life, which requires both geriatric and palliative expertise. However, staff need education about palliative care to be able to provide person-centered care integrated with a focus on symptom management and quality of life for the older people and their family.[24,26] This study is part of an intervention project entitled "Palliative care for elderly people with chronic illnesses: a comparative study between Sweden and China". The purpose of the project is to improve palliative care through work-place education in geriatric hospital care.[27] In order to promote the improvement of the quality of care through the implementation of person-centred palliative care in a Chinese healthcare context, further testing and cross-validation of the original P-CAT was performed on staff in Chinese geriatric palliative hospital settings. A short instrument as P-CAT was preferable in this study due to the need for combinations with additional measurements. It is necessary to ensure that this outcome measure is applicable in the evaluation of educational intervention for staff working in a geriatric hospital. Also, a psychometric evaluation of the original 13 items of the English version means that it should be possible to compare results from Sweden in forthcoming studies within the current intervention project involving Sweden[28] and China.

In China, PCC remains a poorly understood approach that is not generally applied in geriatric services, and one of the possible barriers is the absence of a dependable and valid instrument to measure person-centred care. Therefore, the aim of this study was to accomplish a cultural adaptation of the original English version of the P-CAT for Chinese nurses as well as evaluate the psychometric properties of the newly translated Chinese version applied in geriatric palliative care.

For peer review only - http://bmjopen.bmj.com/site/about/guidelines.xhtml

Methods

This study tested and evaluated the Chinese version of the P-CAT in Mandarin, the most common language in China, on nurses in a hospital context.

The original English instrument

P-CAT measures the extent to which staff engaged in long-term care of the elderly consider their settings to be person-centred. The English P-CAT questionnaire has been validated in an Australian sample of nursing home staff and was shown to have satisfactory psychometric properties.[12] The self-reported questionnaire exists in both a staff version and a version for older persons. In this study, the version for staff was used. It comprises 13 items covering three sub-scales (the Extent of personalizing care, the Amount of organizational support, and the Degree of environmental accessibility). The Extent of personalizing care was measured through items 1-7, Amount of organizational support was measured through items 8-11, and Degree of environmental accessibility was measured through items 12-13. A 5-point Likert-type scale ranging from 1 (strongly disagree) to 5 (strongly agree) was used for scoring. Items 8–12 were negatively worded and the responses were reverse-scored before analysing the data and calculating a total score. Aggregated scores were calculated by means of simple sum scores at the sub-scale and total scale levels. The total score ranged from 13 to 65 and a higher score indicated a higher degree of person-centredness.

Translation and cross-cultural adaptation of the P-CAT

The process of translation and cross-cultural adaptation followed International test commission guidelines.[29,30] Three native Chinese speakers produced independent forward translations from English to Chinese; two had a Public Health degree and the third was a senior nurse who had worked in a department of palliative care for over ten years. First, the translators agreed

For peer review only - http://bmjopen.bmj.com/site/about/guidelines.xhtml

upon a consensus version after discussing the different versions. Then this consensus version was back-translated into English by two new translators who were bilingual and had no knowledge of the procedures of the forward translation. After that, any discrepancies were resolved by an expert committee composed of all the translators, two geriatric physicians, one senior nurse and two university professors. The back-translated version was modified by comparing it with the original version. The expert committee culturally adapted a few phrases to fit the Chinese setting, resulting in a pre-final version. The final version of the Chinese P-CAT was generated after pre-testing for face-validity, involving ten female nurses from a municipal hospital in Kunming, with a mean age of 31.2 years (SD ±8.3). The pre-testing resulted in no changes. The ten staff members who participated in the face-validity testing of the pre-final version did not participate in the later study.

Sample, participants and data collection

The sample was selected to cover hospital nurses working both in urban and suburban areas of Kunming, the capital of the Yunnan province in southwest China. Two municipal hospitals in Kunming were chosen through a convenience sampling method: one hospital from the Wu Hua district (an urban area of Kunming) and one from the Guan Du district (a suburban area of Kunming).

Patients who were admitted to the sampled hospitals had multiple, complex, and overlapping chronic diseases, and most patients were aged 60 years or older. The following criterion for inclusion was used: municipal level hospital with a department of palliative care.

All nurses (n=165) on duty (all shifts on the day of the data collection) at the palliative care departments of the two hospitals were deemed to be eligible for participation and were asked whether they would be willing to participate in the study. The sample size of the study was determined by the criteria suggested by Reise et al.,[31] where the intra-class correlation

For peer review only - http://bmjopen.bmj.com/site/about/guidelines.xhtml

(ICC) >0.8 and five to ten persons for each item of the scale and sample sizes of 100 are often adequate.

Finally, 152 nurses gave their informed consent and completed the Chinese version of the P-CAT questionnaire. The participants were asked to take part in both the test and retest assessments, and all 152 participants completed the first and the follow-up test. This gives an overall response rate of 92.1%. The retest was conducted between one and two weeks after the initial test.

The questionnaires were distributed by two graduate students to all participants, and the completed questionnaires were then anonymously collected on site. In addition to the P-CAT scale, demographic characteristics of the participants were also collected by means of a questionnaire that assessed age, gender, level of education, ethnicity and position.

Psychometric evaluation

Only one item in one questionnaire had a missing value and it was replaced with the mean value of the item for the entire group.[32] An exploratory factor analysis (EFA) with extraction of principal components by both varimax and oblique orthogonal rotation was used to evaluate construct validity. After either direct varimax orthogonal rotating or oblique orthogonal rotating, components in the structural matrix comprised the same numbers of items and loadings, indicating that no difference was found between these two methods in the analysis, and thus only the results of the analysis with varimax orthogonal rotation are presented. Bartlett's test of sphericity and the Keiser-Meyer-Olkin (KMO) measure were applied in order to check the appropriateness of the factor analysis. In order to assess whether the correlation between items was adequate based on a criterion of $p< 0.0001$, Bartlett'stest of sphericity was used, and a criterion of KMO $\geqslant$0.70 was used to indicate sample adequacy. When the Kaiser criterion of eigenvalues was $\geqslant 1$, factors were extracted. A cut-off of 0.50 was applied to

For peer review only - http://bmjopen.bmj.com/site/about/guidelines.xhtml

determine item loadings on each individual component.[33]

The internal consistency and test-retest reliability were evaluated. The internal consistency for the total and sub-scale scores was estimated using Cronbach's alpha coefficient, and Cronbach's alpha ≥0.50 was used as acceptable internal consistency reliability.[34] The item-total correlations were also computed, and the cut-off score for acceptable item-total correlations was set to between 0.3 and 0.8 to ensure moderate correlation and avoid item redundancy.[32] Test-retest reliability was examined with the intra-class correlation (ICC) and Pearson's correlation coefficient (r), where an ICC >0.80 was taken to indicate satisfactory reliability.[35] The paired t-test (2-tailed) was used to verify whether the mean scores from the test and retest differed.

The results of the principal component analysis (PCA) indicate that three factors with eigenvalues above 1 produced a three-component rotated solution that explained 60.2% of the total variance in the data. After direct varimax orthogonal rotating, the structure matrix showed that the first component comprised seven items, the second comprised four items,and the third comprised two items. With regard to the internal consistency reliability, the Cronbach's alpha coefficient of the 13-item Chinese P-CAT was 0.65 for the total scale, 0.67 for the first sub-scale, 0.85 for the second sub-scale and 0.42 for the third sub-scale. Since sub-scale three failed to meet the reliability cut-off of 0.5, the analysis was re-run by forcing the EFA into 2 factors.

The two-factor model was also evaluated by means of a Confirmatory Factor Analysis (CFA) within the framework of Structural Equation Modelling (SEM).[36] The goodness of fit was evaluated using indices of the Normed Fit Index (NFI), the Comparative Fit Index (CFI) and the Root Mean Square Error of Approximation (RMSEA). The results indicated that the goodness of fit of the questionnaire was 0.91 for the NFI, 0.91 for the CFI and 0.73 for the RMSEA. Thus, the CFA supported the exploratory findings. Statistical significance was determined based on two-tailed P values of <0.05. All data analyses were performed using

For peer review only - http://bmjopen.bmj.com/site/about/guidelines.xhtml

SPSS 17.0 software.

Patient and Public Involvement

Patients, relatives, and staff from palliative care were involved in the development of work-place education for staff and informed by patients' priorities, experience, and preferences.[27] It is planned to disseminate the results to staff at meetings about palliative care.

Results

Demographic characteristics of the study group

As described in Table 1, the sample consisted of only female nurses, with a mean age of 30.7 years (SD ±8.7, range 19-53 years). In total, 25.7% of the nurses had a Bachelor's degree or higher. More than one-fourth of the participants reported a minority ethnicity.

Table 1 Demographic characteristics of the study sample (n=152)

Characteristics	n (%)
Gender	
Female	152 (100.0)
Male	0 (0.0)
Age (years)	
18-30	96 (63.2)
31-39	22 (14.5)
⩾40	34 (22.4)
Level of education	
High school	14 (9.2)
Secondary school	39 (25.7)
Junior college	60 (39.5)
Bachelor	39 (25.7)

For peer review only - http://bmjopen.bmj.com/site/about/guidelines.xhtml

Ethnicity	
Han	111 (73.0)
Minorities	41 (27.0)

Construct validity Bartlett's test of sphericity was significant (754.99, $p < 0.0001$) and the KMO measure were satisfactory (0.78), which indicates that correlations between items were sufficient to conduct a principal component analysis (PCA). As shown in Table 2, the results of the PCA indicate that two factors with eigenvalues above 1 produced a two-component rotated solution that explained 50.4% of the total variance in the data. After direct varimax orthogonal rotating, the structural matrix showed that the first component comprised nine items (loadings between 0.16 and 0.79) and the second comprised four items (loadings between 0.71 and 0.89). The two sub-scales were labelled as (1) Extent of personalizing care and (2) Amount of organizational and environmental support.

Table 2 Rotated component matrix for PCA of the 2-factor Chinese P-CAT

**Item number**	**Item content**	**Factor loadings**	
		**Sub-scale 1: Extent of personalizing care**	**Sub-scale 2: Amount of organizational and environmental support**
1	We often discuss how to provide person-centred care.	**0.73**	0.008
2	We have formal team meetings to discuss residents' care.	**0.79**	0.045
3	The life history of the residents is	**0.76**	0.028

For peer review only

For peer review only - http://bmjopen.bmj.com/site/about/guidelines.xhtml

	formally used in the care plans we use.		
4	The quality of the interaction between staff and residents is more important than getting the tasks done.	**0.72**	-0.087
5	We are free to alter work routines based on residents' preferences.	**0.67**	0.117
6	Residents are offered the opportunity to be involved in individualized everyday activities.	**0.54**	-0.109
7	Assessment of residents' needs is undertaken on a daily basis.	**0.17**	0.26
8	I simply do not have the time to provide person-centred care.	-0.208	**0.78**
9	The environment feels chaotic.	-0.096	**0.89**
10	We have to get the work done before we can worry about a homelike environment.	**0.64**	-0.364
11	The organization prevents me from providing person-centred care.	-0.094	**0.89**
12	It is hard for residents in the facility to find their way around.	-0.036	**0.71**
13	Residents are able to access outside space as they wish.	**0.35**	-0.087
Total variance explained (%)	50.4 (total 2 sub-scales)	30.18	20.24
Cronbach's alpha	0.92 (total 13 items)	0.94	0.91

1 Note: Bold number means included in the scale

For peer review only - http://bmjopen.bmj.com/site/about/guidelines.xhtml

Reliability

With regard to the internal consistency reliability, the Cronbach's alpha coefficient of the 13-item Chinese P-CAT was 0.92 for the total scale, 0.94 for the sub-scale Extent of personalized care, and 0.91 for the sub-scale Amount of organizational and environmental support sub-scale (Table 2), indicating that the overall internal consistency reliability was strong. The corrected item-total correlations for all items ranged from 0.14 to 0.44 (Table 3), with six items (items 6, 7, 9, 11, 12 and 13) failing to meet the commonly suggested cut-off level for item-total correlation (>0.3).

Table 3 Item performance and reliability test of the Chinese P-CAT

Item number	Item content	Mean±SD	Corrected item- total correction	Cronbach's alpha if item deleted
1	We often discuss how to provide person-centred care.	4.19±0.70	0.36	0.62
2	We have formal team meetings to discuss residents' care.	4.32±0.66	0.44	0.61
3	The life history of the residents is formally used in the care plans we use.	4.24±0.73	0.41	0.62
4	The quality of the interaction between staff and residents is more important than getting the tasks done.	4.50±0.65	0.32	0.63
5	We are free to alter work routines based on residents' preferences.	4.20±0.77	0.42	0.61

For peer review only - http://bmjopen.bmj.com/site/about/guidelines.xhtml

6	Residents are offered the opportunity to be involved in individualized everyday activities.	3.86±0.82	0.23	0.64
7	Assessment of residents' needs is undertaken on a daily basis.	2.30±1.10	0.19	0.65
8	I simply do not have the time to provide person-centred care.	3.89±0.82	0.35	0.62
9	The environment feels chaotic.	4.11±1.00	0.15	0.65
10	We have to get the work done before we can worry about a homelike environment.	1.74±0.62	0.37	0.62
11	The organization prevents me from providing person-centred care.	4.14±0.92	0.17	0.65
12	It is hard for residents in the facility to find their way around.	2.22±0.88	0.26	0.64
13	Residents are able to access outside space as they wish.	3.89±0.82	0.14	0.65

Table 4 shows the results from the test-retest reliability assessment of the Chinese P-CAT. The results of Pearson's correlation coefficient analysis indicated that the Chinese P-CAT had a high correlation between the test and retest on all scale levels: on the sub-scales Extent of personalizing care ($r=0.90$, $P<0.01$) and Amount of organizational and environmental support ($r=0.88$, $P<0.01$) and on the overall scale ($r=0.88$, $P<0.01$). A paired t-test also confirmed that there was no significant difference between the mean scores of the P-CAT on the test and retest ($P>0.05$). The ICC of the total score between the test and retest was 0.92, providing further support for stating that the scale had satisfactory test-retest reliability.

Table 4 Test-retest reliability of the Chinese P-CAT

For peer review only

Scale dimension	1st test (Mean±SD)	2nd test (Mean±SD)	p	Pearson correlation coefficient (r)	95% CI	ICC (95 % CI)
Extent of personalizing care	33.35±3.59	33.13±3.33	0.09	0.90	(0.83, 0.94)	0.94 (0.92, 0.96)
Amount of organizational and environmental support	14.41±1.72	14.34±1.74	0.34	0.88	(0.76, 0.89)	0.91 (0.87, 0.93)
Overall scale	47.76±3.70	47.46±3.65	0.07	0.88	(0.78, 0.92)	0.92 (0.90, 0.95)

Note: p= two-tailed p-values of paired t-test; ICC: intra-class correlation

Discussion

In this study, the English P-CAT was culturally adapted into a Chinese version. During the translation of the English P-CAT into Chinese, a few minor cultural discrepancies were encountered, and two items of the P-CAT have been modified accordingly. In order to use words closer to the Chinese cultural context and more applicable to a hospital environment, the term ‘resident’ was replaced by ‘patient’ and the term ‘free’ was replaced by ‘flexible’. After cross-cultural adaptation, the pre-testing of the Chinese version of the P-CAT revealed that the items were clearly understood.

This Chinese version of the P-CAT showed a different sub-scale structure as compared to the original English scale, i.e. a structure with three sub-scales (Extent of personalizing care, Amount of organizational support and Degree of environmental accessibility) consisting of 13 items. On the other hand it showed the same sub-scale structure as the Norwegian scale, i.e. a structure which had two sub-scales (Extent of personalizing care and Amount of organizational and environmental support). The 13 items were mostly in concordance with the Norwegian version whereas only item 10 was connected to the first sub-scale.[20] The 2-factor structure has

For peer review only - http://bmjopen.bmj.com/site/about/guidelines.xhtml

also been confirmed in the Swedish version of the P-CAT based on a large cross-sectional sample of 1,465 staff members from 195 residential elderly care units[17] and in a more recent study based on 142 staff working in residential elderly care units and 182 staff working at healthcare centres or home care centres and in social services.[16] Moreover, in this study the Chinese P-CAT showed strong internal consistency reliability with a Cronbach's alpha of 0.92 for the total scale, indicating that the Chinese P-CAT it matched well with the characteristics and structure of the Norwegian version. However, the different sub-scale structures of this Chinese version of the P-CAT as compared to the original English scale may reflect a difference in sample characteristics of staff and the cultural context. The test-retest reliability assessed by ICC was excellent for overall Chinese P-CAT score (0.92) and both sub-scales (0.94 and 0.91), and the excellent test-retest reliability of the Chinese P-CAT was further evidenced through a Pearson's correlation coefficient (r) of 0.90 for the total scale, 0.88 for the Extent of personalizing care sub-scale and 0.88 for the Amount of organizational and environmental support sub-scale. The results of Pearson's correlation coefficient (r) for the total scale in the Chinese version were higher than in the English (0.66),[12] Swedish (0.75),[16-17] Norwegian (0.83)[20] and previous Chinese (0.68)[21] versions, which indicates that this Chinese P-CAT may have a stronger test-retest reliability than other-language versions of the P-CAT.

In this Mandarin version of the Chinese P-CAT, six items (6, 7, 9, 11, 12 and 13) did not meet the commonly suggested cut-off level for item-total correlation (>0.3). In the previous Chinese version of the P-CAT,[21] items 7 and 9 were deleted due to the low value of factor loading (<0.3), and four newly developed items were added to the scale.[21] Item 7 in this study also had a lower value of factor loading than is recommended. However, we decided to include this item in the sub-scale 1, in agreement with the original English version, although this item had a slightly higher value of factor loading in the case of factor 2. Because these two Chinese versions of the P-CAT were evaluated in different healthcare settings (hospital environments

For peer review only - http://bmjopen.bmj.com/site/about/guidelines.xhtml

and residential care facilities), there is no firm consensus regarding the psychometric properties of the Chinese P-CAT, and the findings suggest that the Chinese version requires further investigation and analysis with other samples and in other settings.

In contrast to the low response rate of 21% in the development study of the original English version P-CAT,[12] our study showed a high response rate of 92.1%. Another strength is that the original instrument used was developed from both a clinical and a theoretical perspective on PCC that is seen as the model for good caring from an international perspective.[7,8,12] The model supports an application of the core principles of PC in the specific socio-cultural context of China. The literature suggests a need to see the framework based more on empirical studies from China.[22]

However, some limitations should be noted in this study. First, the P-CAT is primarily intended for use as a research tool to measure the presence of PCC in residential aged care facilities but the present study was conducted only in a hospital context, which may limit the ability to generalize the results to staff working in other healthcare contexts. Further psychometric evaluation of this Chinese P-CAT is needed within different settings to verify the results of this study. Second, from a psychometric evaluation the criterion-related validity was not assessed as a gold standard for the measurement of PCC in hospitals; however, no such criterion exists because of the lack of previous studies in hospital contexts. In addition, the discriminant validity of the Chinese version of P-CAT was not evaluated in our study. Future research should therefore investigate whether the questionnaire has the ability to demonstrate significant differences across hospitals and whether it is useful in discriminating between hospitals in terms of their caring culture. Third, the content validity of the P-CAT was only considered along with the procedures of cross-cultural adaptation. Fourth, only female participants were included in this study, for which reason this study may be subject to gender bias. In interpreting the results, it should be taken into account that this result does not apply to

For peer review only - http://bmjopen.bmj.com/site/about/guidelines.xhtml

the 2.1% male nurses in China (3.5 million nurses).[37-38] Fifth, this study used a cross-sectional design, so it would be useful to conduct a longitudinal study in the future to evaluate change over time.

Palliative care in mainland China has developed slowly since the late 1980s but in recent years profesionals, patients and their families, as well as government, have become increasingly aware of its importance.[39] But even though the provision of palliative care is becoming more and more common in China, the hospice and palliative care services are not meeting the growing demand from the ageing population, and the number of patients with cancer and other life-threatening diseases is increasing.[40] The use of a reliable and valid instrument for the assessment of PCC in research and practice can serve not only to enhance the quality of elderly care but also to strengthen the ongoing development of palliative care in China. Palliative care implies a person-centred approach characterized by a holistic view of the person and a belief that the person should be supported in living a life with dignity. Palliative care strives to make the whole person visible and prioritizes the patient's spiritual, existential, social, and psychological needs to the same extent as physical needs.[41-42] The patient version of P-CAT is used previously by the research group as a complementary assessment in the evaluation of palliative care interventions, going together with instruments for pain management and symptoms relief for the patients.[43] Another application is in the evaluation of work-place education about palliative care for staff in the nursing home context.[28] In this current project,[27] for instance, it is being used in order to investigate whether the staff's attitude to person-centredness has changed as a result of the education.

Conclusion

The results indicated that the 13-item Chinese P-CAT which is a cross-culturally adapted version of the English P-CAT shows strong internal consistency and test-retest reliability and

For peer review only - http://bmjopen.bmj.com/site/about/guidelines.xhtml

appears to be a promising measure for evaluating staff perceptions of person-centredness in a Chinese palliative hospital setting. The results of published psychometric evaluations indicate the use of two scales in the P-CAT as was determined in this study. However, we suggest that the measurement properties of the Chinese version should be further evaluated in other samples and in different healthcare settings. An additional issue for future studies is to evaluate this instrument as a summative measurement with cut-off values for different levels of person-centred care. This can benefit healthcare staff in assessing their quality of care. The results show that P-CAT can be a useful tool for improving the quality of healthcare in terms of PCC in the Chinese context.

Acknowledgements

We would like to thank Ms Huang Jingjing and Ms Wu Chao, two postgraduates of the School of Public Health, Kunming Medical University, who helped us with the data collection, and Dr. Magnus Persson, Lund University for proofreading the manuscript.

Contributors

LC was responsible for the study design, data analysis and drafting of the paper. GA , PT and KM contributed to the study design and provided comments on the paper during the writing process. DE and LB provided comments on the paper during the writing process. HF, JZ and JY were responsible for the data collection. All authors have read and approved the final version of the manuscript.

Competing interests

The authors declare that there are no competing interests.

Funding

For peer review only - http://bmjopen.bmj.com/site/about/guidelines.xhtml

The study was supported by grants from the National Natural Science Fund of China (Grant number: 81611130077) and the Swedish Research Council (Grant number: 2015-06243).

Ethical approval and consent to participate

This study has been approved by the Ethics Committee of Kunming Medical University with the ID number KMU01. Oral and written informed consent was obtained from all persons participating in the study.

Data sharing statement

The datasets used and/or analysed during the current study are available from the corresponding author on reasonable request.

For peer review only

References

1. Pefoyo AJK, Bronskill SE, Gruneir A, et al. The increasing burden and complexity of multimorbidity. *BMC Public Health* 2015;15:415. doi:10.1186/s12889-015-1733-2
2. World population prospects: the 2012 revision. In: Department of Economic and Social Affairs (DoEaSA) PD, ed. New York: United Nations, 2013.
3. Xu J, Chen T, Liu Y, et al. Easily manageable prognostic factors in 152 Chinese elderly acute myeloid leukemia patients: A single-center retrospective study. *J Biomed Res* 2014;28(5):396-405. doi:10.7555/JBR.28.20130164
4. Liao CC, Li CR, Lee SH, et al. Social support and mortality among the aged people with major diseases or ADL disabilities in Taiwan: A national study. *Arch Gerontol Geriatr* 2015;60(2):317-21. doi: 10.1016/j.archger.2014.11.007
5. Nolan MR, Davies S, Brown J, et al. Beyond 'person-centred' care: a new vision for gerontological nursing. *J Clin Nurs* 2004;13(3a):45-53. doi: DOI 10.1111/j.1365-2702.2004.00926.x
6. Edvardsson D, Watt E, Pearce F. Patient experiences of caring and person-centredness are associated with perceived nursing care quality. *J Adv Nurs* 2017;73(1),217-227.doi:10.1111/jan.13105
7. Edvardsson D, Innes A. Measuring Person-centered Care: A Critical Comparative Review of Published Tools. *Gerontologist* 2010;50(6),834-846.doi:10.1093/geront/gnq047
8. McCormack B. A conceptual framework for person-centred practice with older people. *Int J Nurs Pract* 2003;9:202–209. DOI:10.1046/j.1440-172x.2003.00423.x
9. Martinez T, Suárez-Alvarez J, Yanguas J. Instruments for assessing Person Centered Care in Gerontology. *Psicothema* 2016;28(2):114-121.doi:10.7334/psicothema2015.263
10. Brownie S, Nancarrow S. Effects of person-centered care on residents and staff in aged-care facilities: a systematic review. *Clin Interv Aging* 2013;8:1–10.doi:10.2147/CIA.S38589
11. Edvardsson D, Sandman PO, Rasmussen B. Construction and psychometric evaluation of the Swedish language Person-centred Climate Questionnaire - staff version. *J Nurs Manag* 2009;17(7):790-5.doi: 10.1111/j.1365-2834.2009.01005.x
12. Edvardsson D, Fetherstonhaugh D, Nay R, et al. Development and initial testing of the Person-centered Care Assessment Tool (P-CAT). *Int Psychogeriatr* 2010;22(1):101-08. doi:10.1017/S1041610209990688
13. White DL, Newton-Curtis L, Lyons KS. Development and initial testing of a measure of person-directed care. *Gerontologist* 2008;48.DOI:10.1093/geront/48.supplement_1.114-123
14. Chappell NL, Colin Reid R, Gish JA. Staff-based measures of individualized care for persons with dementia in long-term care facilities. *Dementia* 2007;6(4):527-47. doi:10.1177/1471301207084372
15. Zimmerman S, Allen J, Cohen LW, et al. A Measure of Person-Centered Practices in Assisted Living: The PC-PAL. *J Am Med Dir Assoc* 2015;16(2):132-137. doi:10.1016/j.jamda.2014.07.016
16. Selan D, Jakobsson U, Condelius A. The Swedish P-CAT: modification and exploration of psychometric properties of two different versions. *Scand J Caring Sci* 2017;31(3):527-35.doi: 10.1111/scs.12366
17. Sjogren K, Lindkvist M, Sandman PO, et al. Psychometric evaluation of the Swedish version of the Person-Centered Care Assessment Tool (P-CAT). *Int Psychogeriatr* 2012;24(3):406-415.doi: 10.1017/S104161021100202x
18. Tak YR, Woo HY, You SY, et al. [Validity and Reliability of the Person-centered Care Assessment Tool in Long-term Care Facilities in Korea]. *J Korean Acad Nurs* 2015;45(3):412-419.doi: 10.4040/jkan.2015.45.3.412
19. Martinez T, Suarez-Alvarez J, Yanguas J, et al. Spanish validation of the Person-centered Care Assessment Tool (P-CAT). *Aging Ment Health* 2016;20(5):550-58.doi: 10.1080/13607863.2015.1023768
20. Rokstad AMM, Engedal K, Edvardsson D, et al. Psychometric evaluation of the Norwegian version of the Person-centred Care Assessment Tool. *Int J Nurs Pract* 2012;18(1):99-105.doi: 10.1111/j.1440-172X.2011.01998.x
21. Zhong XB, Lou VW. Person-centered care in Chinese residential care facilities: a preliminary

For peer review only - http://bmjopen.bmj.com/site/about/guidelines.xhtml

measure. *Aging Ment Health* 2013;17(8):952-58.doi:10.1080/13607863.2013.790925

22. Wang J, Wu B, Bowers BJ, et al. Person-centered dementia care in China: A bilingual literature review. *Gerontol Geriatr Med* 2019;5:1-11.doi:10.1177/2333721419844349.
23. Mao P, Xiao D, Zhang M, et al. Person-centered dementia care in eldercare institutions in Hunan province. *Journal of Nursing Science* 2016;31(21):1-4.
24. Hall S, Petkova H, Tsouros A, et al. Palliative care for older people: better practices. Copenhagen: WHO Regional Office for Europe; 2011. (Accessed October 26, 2019). http://www.euro.who.int/__data/assets/pdf_file/0017/143153/e95052.pdf
25. Davies E, Higginson IJ. Better palliative care for older people. WHO Regional Office for Europe; 2004. (Accessed October 26, 2019). http://www.euro.who.int/__data/assets/pdf_file/0009/98235/E82933.pdf
26. Price DM, Strodtman LK, Montagnini M, et al. Health professionals perceived concerns and challenges in providing palliative and end-of-life care: A qualitative analysis. *Am J Hosp Palliat Care* 2019 Apr;36(4):308-315.doi:10.1177/1049909118812193. Epub 2018 Nov 21.
27. Ahlström G, Benzein E. Themed meetings about palliative care within health care (Additional file 1, The Booklet). In: Bökberg C, Behm L, Wallerstedt B & Ahlström G, editors. Evaluation of person-centeredness in nursing homes after a palliative care intervention: pre and post-test experimental design. *BMC Palliat Care* 2019;18(1):44. doi:10.1186/s12904-019-0431-8.
28. Bökberg C, Behm L, Wallerstedt B, et al. Evaluation of person-centeredness in nursing homes after a palliative care intervention: pre- and post-test experimental design. *BMC Palliat Care* 2019; 18:44. https://doi.org/10.1186/s12904-019-0431-8.
29. Guillemin F. Cross-Cultural Adaptation and Validation of Health-Status Measures. *Scand J Rheumatol* 1995;24(2):61-63. doi: Doi 10.3109/03009749509099285
30. Beaton DE, Bombardier C, Guillemin F, et al. Guidelines for the process of cross-cultural adaptation of self-report measures. *Spine* 2000;25(24):3186-91. doi: Doi 10.1097/00007632-200012150-00014
31. Reise SP, Waller NG, Comrey AL. Factor analysis and scale revision. *Psychol Assessment* 2000;12(3):287-97. doi: 10.1037//1040-3590.12.3.287
32. Streiner DL, Norman GR, Cairney J. Health Measurement Scales: A Practical Guide to Their Development and Use. Fifth edition ed. 2014, Oxford: Oxford University Press.
33. Pett MA, Lackey NR, Sullivan JJ. Making sense of factor analysis : the use of factor analysis for instrument development in health care research: London : SAGE, cop. 2003.
34. Bland JM, Altman DG. Cronbach's alpha. *Brit Med J* 1997;314(7080):572-72. doi: DOI 10.1136/bmj.314.7080.572
35. Landis JR, Koch GG. The measurement of observer agreement for categorical data. *Biometrics* 1977;33(1):159-74. [published Online First: 1977/03/01]
36. Kline RB. Principles and Practice of Structural Equation Modeling. Fourth Edition. New York: Guilford Press 2015.
37. National Health Commission of the People's Republic of China. 2017 yearbook of health and family planning statistics in China. Beijing: China Union Medical University Press, 2017.
38. Yang J-S, Hao D-J. Dilemmas for nurses in China. Correspondence. *Lancet* 2018;392(10141):30.
39. Ning XH. Hospice and palliative care in mainland China: History, Current status and challenges. Chin Med Sci J 2018; 33(4): 199-203. doi: 10.24920/003524.
40. Lu Y, Gu Y, Yu W. Hospice and palliative care in China: Development and challenges. *Asia Pac J Oncol Nurs* 2018;5:26-32.
41. McCormack B, McCance TV. Development of a framework for personcentred nursing. *J Adv Nurs* 2006. https://doi.org/10.1111/j.1365-2648.2006.04042.x.
42. McCormack B, Dewing J, Breslin L, et al. Developing person-centred practice: nursing outcomes arising from changes to the care environment in residential settings for older people. *Int J Older people Nurs* 2010;5(2):93-107.doi:10.1111/j.1748-3743.2010.00216.x [published Online First: 2010/10/12]
43. Bökberg C, Behm L, Wallerstedt B, et al. Evaluating person-centredness for frail older persons in nursing homes before and after implementing a palliative care intervention. *Nursing Open* 2019;00:1–10. DOI: 10.1002/nop2.408

For peer review only - http://bmjopen.bmj.com/site/about/guidelines.xhtml

BMJ Open

Psychometric evaluation of the Chinese version of the Person-centred Care Assessment Tool

Journal:	*BMJ Open*
Manuscript ID	bmjopen-2019-031580.R3
Article Type:	Original research
Date Submitted by the Author:	02-Mar-2020

Complete List of Authors:	Le, Cai; Kunming Medical University in Kunming,<br>Ma, Ke; The third people's hospital of Kunming, Palliative Care<br>Tang, Pingfen; Kunming Medical University<br>Edvardsson, David; La Trobe University School of Nursing and Midwifery<br>Behm, Lina; Kristianstad University College, Faculty of Health Sciences<br>Zhang, Jie; the third people's hospital of Kunming, palliative care<br>Yang, Jiqun; the third people's hospital of Kunming<br>Fu, Haiyan; the third people's hospital of Kunming<br>Ahlström, Gerd; Lunds Universitet, Health Sciences

**Primary Subject Heading**:	Geriatric medicine
Secondary Subject Heading:	Palliative care, Nursing, Evidence based practice
Keywords:	Person-centered care, older people, PALLIATIVE CARE, instrument evaluation, factor analysis

SCHOLARONE™
Manuscripts

I, the Submitting Author has the right to grant and does grant on behalf of all authors of the Work (as defined in the below author licence), an exclusive licence and/or a non-exclusive licence for contributions from authors who are: i) UK Crown employees; ii) where BMJ has agreed a CC-BY licence shall apply, and/or iii) in accordance with the terms applicable for US Federal Government officers or employees acting as part of their official duties; on a worldwide, perpetual, irrevocable, royalty-free basis to BMJ Publishing Group Ltd ("BMJ") its licensees and where the relevant Journal is co-owned by BMJ to the co-owners of the Journal, to publish the Work in this journal and any other BMJ products and to exploit all rights, as set out in our licence.

The Submitting Author accepts and understands that any supply made under these terms is made by BMJ to the Submitting Author unless you are acting as an employee on behalf of your employer or a postgraduate student of an affiliated institution which is paying any applicable article publishing charge ("APC") for Open Access articles. Where the Submitting Author wishes to make the Work available on an Open Access basis (and intends to pay the relevant APC), the terms of reuse of such Open Access shall be governed by a Creative Commons licence – details of these licences and which Creative Commons licence will apply to this Work are set out in our licence referred to above.

Other than as permitted in any relevant BMJ Author's Self Archiving Policies, I confirm this Work has not been accepted for publication elsewhere, is not being considered for publication elsewhere and does not duplicate material already published. I confirm all authors consent to publication of this Work and authorise the granting of this licence.

For peer review only

Psychometric evaluation of the Chinese version of the Person-centred Care Assessment Tool

Running title: Evaluation of the Chinese version of P-CAT

Le Cai, PhD[1], Ke Ma, MD[2], Pingfen Tang, RN[1], David Edvardsson, PhD[3], Lina Behm, PhD[4], Jie Zhang, MD[5], Jiqun Yang, MD[6], Haiyan Fu, MD[5] and Gerd Ahlström, PhD[7]*

* Corresponding author: Professor Gerd Ahlström, Department of Health Sciences, Faculty of Medicine, Lund University, Box 157, SE-221 00 Sweden. Email: gerd.ahlstrom@med.lu.se Phone: +46 46 702 747767

[1] PhD, Professor, School of Public Health, Kunming Medical University, Cheng Gong New City, Kunming, China

[2] Affiliated physician, Division of palliative care, the Third People's Hospital of Kunming, China

[3] Professor, Austin Health/Northern Health Clinical Schools of Nursing College of Science, Health and Engineering, La Trobe University, Australia

[4] PhD, Senior Lecturer, Department of nursing educations and integrated health science, Kristianstad University, Sweden

[5] Physician, Division of palliative care, the Third People's Hospital of Kunming, China

[6] Vice-Director, Division of palliative care, the Third People's Hospital of Kunming, China

[7] PhD, Professor, Department of Health Sciences, Faculty of Medicine, Lund University, Sweden. ORCID no 0000-0001-6230-7583

Word count: 4340 words (without abstract, tables and reference list)

For peer review only

Abstract

Objective: This study aims to test a Chinese cross-cultural adaptation of the English version of the P-CAT and evaluate its psychometric properties.

Design: P-CAT was translated/back-translated using established procedures. Psychometric evaluation of the Chinese version of the Person-centred Care Assessment Tool.

Setting: Two hospitals covering urban and suburban areas of Kunming, in the Yunnan province of China.

Participants: 152 female hospital staff completed the survey

Main outcome measure(s): Construct validity and reliability, including internal consistency and test-retest reliability, were assessed among a sample of hospital staff.

Results: The factor analysis resulted in a two-component solution that consisted of two sub-scales. The corrected item-total correlations for all of the items ranged from 0.14 to 0.44, with six items that did not meet the cut-off level for item-total correlation (>0.3). The Chinese P-CAT demonstrated strong reliability, with a Cronbach's alpha of 0.91-0.94 for the scales, and a test-retest reliability coefficient of 0.88 for the overall scale scores. The ICC was 0.92 (95 % CI: 0.90- 0.95).

Conclusion: P-CAT appears to be a promising measure for evaluating staff perceptions of the person-centredness in Chinese hospital environments. The results show that P-CAT can be a useful tool for improving the quality of healthcare in terms of person-centred care in the Chinese context.

Keywords: Person-centered care, older people, palliative care, instrument evaluation, factor analysis

For peer review only

Article summary

Strengths and limitations of this study

- A previous study found the need for revision of the original P-CAT questionnaire. This study validates a translated and culturally adapted but otherwise unmodified English version of P-CAT in a Chinese context.
- High response rate (92.1%).
- Convenience sampling method may limit the ability to generalize the results.
- The Chinese P-CAT has been tested only in this geriatric hospital setting.

For peer review only

Introduction

Population ageing is occurring globally, and multi-morbidity is highly prevalent, particularly in older people, who are generally frequent healthcare users.[1] The Chinese population is one of the fastest-ageing populations in the world, and the number of people aged 60 years and older is expected to rise from 193 million (13.9%) in 2013 to 454 million (32.8%) in 2050.[2] This rapid increase in the number of older people in China is reflected in the increasing number of older people admitted to hospitals. The National Health Services survey of China shows that the hospitalization rate of people aged 65 years and older increased significantly from 6.1% to 19.9% during the period 2003–2013,[3] and chronic diseases occur more frequently in older people and are more difficult to cure when multiple diseases coexist.[4] Thus, the healthcare system in China is facing great challenges due to the aging population who require high-quality care from a wide range of multiple care professionals.

Person-centred care (PCC) is now regarded as a valuable approach to use when it comes to improving long-term care for older people,[5] and the quality of care is often described in terms of its being person-centred.[6-8] The growing interest in applying PCC is accompanied by an interest in developing instruments, commonly questionnaires answered by staff as a proxy for the ill person.[7,9] The core values of PCC are the social relationship between the staff and the ill person and the close family, characterized by respect for the person's will, mutuality, openness, honesty, and sympathetic presence.[8] Even though there is a range of terms in the literature for describing PCC, all imply a similar philosophy of looking at the person's needs instead of a disease.[7] A review shows that person-centred interventions are multifactorial and that the complexity of the interventions can explain the range of existing outcome variables.[10]

To date, a number of instruments have been developed to assess the PCC in residential care facilities for older people, and these instruments are mainly based on the opinions of staff working in such facilities. The instruments are the English Person-centred Climate

For peer review only - http://bmjopen.bmj.com/site/about/guidelines.xhtml

Questionnaire – Staff version (PCQ-S),[11] the Person-centred Care Assessment Tool (P-CAT),[12] the Staff Assessment Person-Directed Care tool (PDC),[13] the Individualized Care tool (IC)[14] and the Staff Person-Centred Practices in Assisted Living tool (Staff PC-PAL).[15] Of these, the tool most commonly used internationally, with established credibility, is the P-CAT.[7,9] The P-CAT questionnaire was designed by Edvardsson and colleagues[12] and involves a caregiver-based self-report assessment scale used to measure the extent to which the staff agree that a number of pre-defined characteristics/processes of PCC are in place within their team, unit or nursing home. The P-CAT is easy to use, with only 13 items. It has a process-focus, as evidenced in items connected with work routines, residents' life stories, assessment of personal needs, and discussion concerning best care for the residents. It has been validated and used in English,[12] Swedish,[16-17] Korean,[18] Spanish,[19] Norwegian,[20] and Chinese.[21]

A recently published literature review [22] indicates that the studies of PCC were predominantly on populations in Western Europe and the USA. However, the increasing proportion of older people implies an urgent need for the implementation of PCC for the purpose of improving the quality of care. The study of Zhong and colleagues[21] found that the P-CAT was a good tool to adapt for use in the Chinese context. A 24-item Chinese version of the original English 13-item P-CAT was developed and later reduced to 15 items as a result of psychometric validation on staff. This modified version of P-CAT was changed to such an extent as to make it impossible to compare the results with results obtained by use of the original English version. Two items were removed and four items were added, generating three scales.[21] This Chinese version was validated on staff working in residential care for patients with dementia because P-CAT was originally intended for use as a research instrument to measure the presence of PCC in this context.[12,23]

Palliative care is provided in a person-centred environment, where the older person and the family feel welcome, feel that they are seen, feel that they are involved by the staff.

For peer review only - http://bmjopen.bmj.com/site/about/guidelines.xhtml

This type of supportive care has been developed in hospice settings by specialists in the care of patients dying from incurable cancer, and older persons dying from other diseases do not have the same access to palliative care.[24-25] The ageing people with multiple morbidities have complex care needs and multiple symptoms in their last period of life, which requires both geriatric and palliative expertise. However, staff need education about palliative care to be able to provide person-centered care integrated with a focus on symptom management and quality of life for the older people and their family.[24,26]

To date, palliative care is widely provided in Western countries, but access to such service is extremely limited in mainland China,[27] especially outside oncology departments. Since palliative care is still in its initial stage and not enough in respect of the aging population and the number of patients with life-threatening diseases that are increasing in China.[27-28] Therefore, the intervention project entitled "Palliative care for elderly people with chronic illnesses: a comparative study between Sweden and China" was initiated. The purpose of the project is to improve palliative care through work-place education in geriatric hospital care.[29] In order to promote the improvement of the quality of care through the implementation of person-centred palliative care in a Chinese healthcare context, further testing and cross-validation of the original P-CAT was performed on staff in Chinese geriatric palliative hospital settings. A short instrument as P-CAT was preferable in this study due to the need for combinations with additional measurements. It is necessary to ensure that this outcome measure is applicable in the evaluation of educational intervention for staff working in a geriatric hospital. Also, a psychometric evaluation of the original 13 items of the English version means that it should be possible to compare results from Sweden in forthcoming studies within the current intervention project involving Sweden[30] and China.

In China, PCC remains a poorly understood approach that is not generally applied in geriatric services, and one of the possible barriers is the absence of a dependable and valid

For peer review only - http://bmjopen.bmj.com/site/about/guidelines.xhtml

instrument to measure person-centred care. Therefore, the aim of this study was to accomplish a cultural adaptation of the original English version of the P-CAT for Chinese nurses as well as evaluate the psychometric properties of the newly translated Chinese version applied in geriatric palliative care.

Methods

This study tested and evaluated the Chinese version of the P-CAT in Mandarin, the most common language in China, on nurses in a hospital-based palliative care setting. This psychometric study is part of the intervention project "Palliative care for elderly people with chronic illnesses: a comparative study between Sweden and China".

The original English instrument

P-CAT measures the extent to which staff engaged in long-term care of the elderly consider their settings to be person-centred. The English P-CAT questionnaire has been validated in an Australian sample of nursing home staff and was shown to have satisfactory psychometric properties.[12] The self-reported questionnaire exists in both a staff version and a version for older persons. In this study, the version for staff was used. It comprises 13 items covering three sub-scales (the Extent of personalizing care, the Amount of organizational support, and the Degree of environmental accessibility). The Extent of personalizing care was measured through items 1-7, Amount of organizational support was measured through items 8-11, and Degree of environmental accessibility was measured through items 12-13. A 5-point Likert-type scale ranging from 1 (strongly disagree) to 5 (strongly agree) was used for scoring. Items 8–12 were negatively worded and the responses were reverse-scored before analysing the data and calculating a total score. Aggregated scores were calculated by means of simple sum scores at the sub-scale and total scale levels. The total score ranged from 13 to 65 and a higher score indicated a higher degree of person-centredness.

For peer review only - http://bmjopen.bmj.com/site/about/guidelines.xhtml

Translation and cross-cultural adaptation of the P-CAT

The process of translation and cross-cultural adaptation followed International test commission guidelines.[31,32] Three native Chinese speakers produced independent forward translations from English to Chinese; two had a Public Health degree and the third was a senior nurse who had worked in a department of palliative care for over ten years. First, the translators agreed upon a consensus version after discussing the different versions. Then this consensus version was back-translated into English by two new translators who were bilingual and had no knowledge of the procedures of the forward translation. After that, any discrepancies were resolved by an expert committee composed of all the translators, two geriatric physicians, one senior nurse and two university professors. The back-translated version was modified by comparing it with the original version. The expert committee culturally adapted a few phrases to fit the Chinese setting, resulting in a pre-final version. The final version of the Chinese P-CAT was generated after pre-testing for face-validity, involving ten female nurses from a municipal hospital in Kunming, with a mean age of 31.2 years (SD ±8.3). The pre-testing resulted in no changes. The ten staff members who participated in the face-validity testing of the pre-final version did not participate in the later study.

Sample, participants and data collection

The sample was selected to cover hospital nurses working with palliative care both in urban and suburban areas of Kunming, the capital of the Yunnan province in southwest China. Two municipal hospitals in Kunming were chosen through a convenience sampling method: one hospital from the Wu Hua district (an urban area of Kunming) and one from the Guan Du district (a suburban area of Kunming).

Patients who were admitted to the sampled hospitals had multiple, complex, and

For peer review only - http://bmjopen.bmj.com/site/about/guidelines.xhtml

overlapping chronic diseases, and most patients were aged 60 years or older. The following criterion for inclusion was used: municipal level hospital with a department of palliative care.

All nurses (n=165) on duty (all shifts on the day of the data collection) at the palliative care departments of the two hospitals were deemed to be eligible for participation and were asked whether they would be willing to participate in the study. The sample size of the study was determined by the criteria suggested by Reise et al.,[33] where the intra-class correlation (ICC) >0.8 and five to ten persons for each item of the scale and sample sizes of 100 are often adequate.

Finally, 152 nurses gave their informed consent and completed the Chinese version of the P-CAT questionnaire. The participants were asked to take part in both the test and retest assessments, and all 152 participants completed the first and the follow-up test. This gives an overall response rate of 92.1%. The retest was conducted between one and two weeks after the initial test.

The questionnaires were distributed by two graduate students to all participants, and the completed questionnaires were then anonymously collected on site. In addition to the P-CAT scale, demographic characteristics of the participants were also collected by means of a questionnaire that assessed age, gender, level of education, ethnicity and position.

Psychometric evaluation

Only one item in one questionnaire had a missing value and it was replaced with the mean value of the item for the entire group.[34] An exploratory factor analysis (EFA) with extraction of principal components by both varimax and oblique rotation was used to evaluate construct validity. After either direct varimax rotating or oblique rotating, components in the structural matrix comprised the same numbers of items and loadings, indicating that no difference was found between these two methods in the analysis, and thus only the results of the analysis with

For peer review only - http://bmjopen.bmj.com/site/about/guidelines.xhtml

varimax rotation are presented. Bartlett's test of sphericity and the Keiser-Meyer-Olkin (KMO) measure were applied in order to check the appropriateness of the factor analysis. In order to assess whether the correlation between items was adequate based on a criterion of $p < 0.0001$, Bartlett'stest of sphericity was used, and a criterion of KMO $\geqslant 0.70$ was used to indicate sample adequacy. When the Kaiser criterion of eigenvalues was $\geqslant 1$, factors were extracted. A cut-off of 0.50 was applied to determine item loadings on each individual component.[35]

The internal consistency and test-retest reliability were evaluated. The internal consistency for the total and sub-scale scores was estimated using Cronbach's alpha coefficient, and Cronbach's alpha ≥ 0.50 was used as acceptable internal consistency reliability.[36] The item-total correlations were also computed, and the cut-off score for acceptable item-total correlations was set to between 0.3 and 0.8 to ensure moderate correlation and avoid item redundancy.[34] Test-retest reliability was examined with the intra-class correlation (ICC) and Pearson's correlation coefficient (r), where an ICC >0.80 was taken to indicate satisfactory reliability.[37] The paired t-test (2-tailed) was used to verify whether the mean scores from the test and retest differed.

The results of the principal component analysis (PCA) indicate that three factors with eigenvalues above 1 produced a three-component rotated solution that explained 60.2% of the total variance in the data. After direct varimax orthogonal rotating, the structure matrix showed that the first component comprised seven items, the second comprised four items, and the third comprised two items. With regard to the internal consistency reliability, the Cronbach's alpha coefficient of the 13-item Chinese P-CAT was 0.65 for the total scale, 0.67 for the first sub-scale, 0.85 for the second sub-scale and 0.42 for the third sub-scale. Since sub-scale three failed to meet the reliability cut-off of 0.5, the analysis was re-run by forcing the EFA into 2 factors.

The two-factor model was also evaluated by means of a Confirmatory Factor Analysis (CFA) within the framework of Structural Equation Modelling (SEM).[38] The goodness of fit was

For peer review only - http://bmjopen.bmj.com/site/about/guidelines.xhtml

evaluated using indices of the Normed Fit Index (NFI), the Comparative Fit Index (CFI) and the Root Mean Square Error of Approximation (RMSEA). The results indicated that the goodness of fit of the questionnaire was 0.91 for the NFI, 0.91 for the CFI and 0.73 for the RMSEA. Thus, the CFA supported the exploratory findings. Statistical significance was determined based on two-tailed P values of <0.05. All data analyses were performed using SPSS 22.0 software, and the two-factor model was evaluated by means of a CFA within the framework of SEM with SPSS 22.0 under the Package ‘Analysis of Moment Structures’ (AMOS).

Patient and Public Involvement

Patients, relatives, and staff from palliative care were involved in the development of work-place education for staff and informed by patients’ priorities, experience, and preferences.[29] It is planned to disseminate the results to staff at meetings about palliative care.

Results

Demographic characteristics of the study group

As described in Table 1, the sample consisted of only female nurses, with a mean age of 30.7 years (SD ±8.7, range 19-53 years). In total, 25.7% of the nurses had a Bachelor’s degree or higher. More than one-fourth of the participants reported a minority ethnicity.

For peer review only - http://bmjopen.bmj.com/site/about/guidelines.xhtml

Table 1 Demographic characteristics of the study sample (n=152)

**Characteristics**	**n (%)**
Gender	
Female	152 (100.0)
Male	0 (0.0)
Age (years)	
18-30	96 (63.2)
31-39	22 (14.5)
⩾40	34 (22.4)
Level of education	
High school	14 (9.2)
Secondary school	39 (25.7)
Junior college	60 (39.5)
Bachelor	39 (25.7)
Ethnicity	
Han	111 (73.0)
Minorities	41 (27.0)

Construct validity Bartlett's test of sphericity was significant (754.99, p< 0.0001) and the KMO measure were satisfactory (0.78), which indicates that correlations between items were sufficient to conduct a principal component analysis (PCA). As shown in Table 2, the results of the PCA indicate that two factors with eigenvalues above 1 produced a two-component rotated solution that explained 50.4% of the total variance in the data. After direct varimax orthogonal rotating, the structural matrix showed that the first component comprised nine items (loadings between 0.16 and 0.79) and the second comprised four items (loadings between 0.71 and 0.89). The two sub-scales were labelled as (1) Extent of personalizing care and (2) Amount of organizational and environmental support.

For peer review only - http://bmjopen.bmj.com/site/about/guidelines.xhtml

For peer review only

1 **Table 2** Rotated component matrix for PCA of the 2-factor Chinese P-CAT

Item number	Item content	Factor loadings	
		**Sub-scale 1: Extent of personalizing care**	**Sub-scale 2: Amount of organizational and environmental support**
1	We often discuss how to provide person-centred care.	**0.73**	0.008
2	We have formal team meetings to discuss residents' care.	**0.79**	0.045
3	The life history of the residents is formally used in the care plans we use.	**0.76**	0.028
4	The quality of the interaction between staff and residents is more important than getting the tasks done.	**0.72**	-0.087
5	We are free to alter work routines based on residents' preferences.	**0.67**	0.117
6	Residents are offered the opportunity to be involved in individualized everyday activities.	**0.54**	-0.109
7	Assessment of residents' needs is undertaken on a daily basis.	**0.17**	0.26
8	I simply do not have the time to provide person-centred care.	-0.208	**0.78**
9	The environment feels chaotic.	-0.096	**0.89**

For peer review only - http://bmjopen.bmj.com/site/about/guidelines.xhtml

10	We have to get the work done before we can worry about a homelike environment.	**0.64**	-0.364
11	The organization prevents me from providing person-centred care.	-0.094	**0.89**
12	It is hard for residents in the facility to find their way around.	-0.036	**0.71**
13	Residents are able to access outside space as they wish.	**0.35**	-0.087
Total variance explained (%)	50.4 (total 2 sub-scales)	30.18	20.24
Cronbach's alpha	0.92 (total 13 items)	0.94	0.91

1 Note: Bold number means included in the scale

2

3 **Reliability**

4 With regard to the internal consistency reliability, the Cronbach's alpha coefficient of the 13-
5 item Chinese P-CAT was 0.92 for the total scale, 0.94 for the sub-scale Extent of personalized
6 care, and 0.91 for the sub-scale Amount of organizational and environmental support sub-scale
7 (Table 2), indicating that the overall internal consistency reliability was strong. The corrected
8 item-total correlations for all items ranged from 0.14 to 0.44 (Table 3), with six items (items 6,
9 7, 9, 11, 12 and 13) failing to meet the commonly suggested cut-off level for item-total
10 correlation (>0.3).

11

12

For peer review only - http://bmjopen.bmj.com/site/about/guidelines.xhtml

1 **Table 3** Item performance and reliability test of the Chinese P-CAT

Item number	Item content	Mean±SD	Corrected item- total correction	Cronbach's alpha if item deleted
1	We often discuss how to provide person-centred care.	4.19±0.70	0.36	0.62
2	We have formal team meetings to discuss residents' care.	4.32±0.66	0.44	0.61
3	The life history of the residents is formally used in the care plans we use.	4.24±0.73	0.41	0.62
4	The quality of the interaction between staff and residents is more important than getting the tasks done.	4.50±0.65	0.32	0.63
5	We are free to alter work routines based on residents' preferences.	4.20±0.77	0.42	0.61
6	Residents are offered the opportunity to be involved in individualized everyday activities.	3.86±0.82	0.23	0.64
7	Assessment of residents' needs is undertaken on a daily basis.	2.30±1.10	0.19	0.65
8	I simply do not have the time to provide person-centred care.	3.89±0.82	0.35	0.62
9	The environment feels chaotic.	4.11±1.00	0.15	0.65
10	We have to get the work done before we can worry about a homelike environment.	1.74±0.62	0.37	0.62
11	The organization prevents me from providing person-centred care.	4.14±0.92	0.17	0.65
12	It is hard for residents in the facility to find	2.22±0.88	0.26	0.64

For peer review only - http://bmjopen.bmj.com/site/about/guidelines.xhtml

	their way around.			
13	Residents are able to access outside space as they wish.	3.89±0.82	0.14	0.65

Table 4 shows the results from the test-retest reliability assessment of the Chinese P-CAT. The results of Pearson's correlation coefficient analysis indicated that the Chinese P-CAT had a high correlation between the test and retest on all scale levels: on the sub-scales Extent of personalizing care (r=0.90, P<0.01) and Amount of organizational and environmental support (r=0.88, P<0.01) and on the overall scale (r=0.88, P<0.01). A paired t-test also confirmed that there was no significant difference between the mean scores of the P-CAT on the test and retest (P>0.05). The ICC of the total score between the test and retest was 0.92, providing further support for stating that the scale had satisfactory test-retest reliability.

For peer review only

Table 4 Test-retest reliability of the Chinese P-CAT

**Scale dimension**	**1st test (Mean±SD)**	**2nd test (Mean±SD)**	**p**	**Pearson correlation coefficient (r)**	**95% CI**	**ICC (95 % CI)**
Extent of personalizing care	33.35±3.59	33.13±3.33	0.09	0.90	(0.83, 0.94)	0.94 (0.92, 0.96)
Amount of organizational and environmental support	14.41±1.72	14.34±1.74	0.34	0.88	(0.76, 0.89)	0.91 (0.87, 0.93)
Overall scale	47.76±3.70	47.46±3.65	0.07	0.88	(0.78, 0.92)	0.92 (0.90, 0.95)

Note: p= two-tailed p-values of paired t-test; ICC: intra-class correlation

For peer review only - http://bmjopen.bmj.com/site/about/guidelines.xhtml

Discussion

In this study, the English P-CAT was culturally adapted into a Chinese version. During the translation of the English P-CAT into Chinese, a few minor cultural discrepancies were encountered, and two items of the P-CAT have been modified accordingly. In order to use words closer to the Chinese cultural context and more applicable to a hospital environment, the term 'resident' was replaced by 'patient' and the term 'free' was replaced by 'flexible'. After cross-cultural adaptation, the pre-testing of the Chinese version of the P-CAT revealed that the items were clearly understood.

This Chinese version of the P-CAT showed a different sub-scale structure as compared to the original English scale, i.e. a structure with three sub-scales (Extent of personalizing care, Amount of organizational support and Degree of environmental accessibility) consisting of 13 items. On the other hand it showed the same sub-scale structure as the Norwegian scale, i.e. a structure which had two sub-scales (Extent of personalizing care and Amount of organizational and environmental support). The 13 items were mostly in concordance with the Norwegian version whereas only item 10 was connected to the first sub-scale.[20] The 2-factor structure has also been confirmed in the Swedish version of the P-CAT based on a large cross-sectional sample of 1,465 staff members from 195 residential elderly care units[17] and in a more recent study based on 142 staff working in residential elderly care units and 182 staff working at healthcare centres or home care centres and in social services.[16] Moreover, in this study the Chinese P-CAT showed strong internal consistency reliability with a Cronbach's alpha of 0.92 for the total scale, indicating that the Chinese P-CAT it matched well with the characteristics and structure of the Norwegian version. However, the different sub-scale structures of this Chinese version of the P-CAT as compared to the original English scale may reflect a difference in sample characteristics of staff and the cultural context. The test-retest reliability assessed by ICC was excellent for overall Chinese P-CAT score (0.92) and both sub-scales

For peer review only

(0.94 and 0.91), and the excellent test-retest reliability of the Chinese P-CAT was further evidenced through a Pearson's correlation coefficient (r) of 0.90 for the total scale, 0.88 for the Extent of personalizing care sub-scale and 0.88 for the Amount of organizational and environmental support sub-scale. The results of Pearson's correlation coefficient (r) for the total scale in the Chinese version were higher than in the English (0.66),[12] Swedish (0.75),[16-17] Norwegian (0.83)[20] and previous Chinese (0.68)[21] versions, which indicates that this Chinese P-CAT may have a stronger test-retest reliability than other-language versions of the P-CAT.

In this Mandarin version of the Chinese P-CAT, six items (6, 7, 9, 11, 12 and 13) did not meet the commonly suggested cut-off level for item-total correlation (>0.3). In the previous Chinese version of the P-CAT,[21] items 7 and 9 were deleted due to the low value of factor loading (<0.3), and four newly developed items were added to the scale.[21] Item 7 in this study also had a lower value of factor loading than is recommended. However, we decided to include this item in the sub-scale 1, in agreement with the original English version, although this item had a slightly higher value of factor loading in the case of factor 2. Because these two Chinese versions of the P-CAT were evaluated in different healthcare settings (hospital environments and residential care facilities), there is no firm consensus regarding the psychometric properties of the Chinese P-CAT, and the findings suggest that the Chinese version requires further investigation and analysis with other samples and in other settings.

In contrast to the low response rate of 21% in the development study of the original English version P-CAT,[12] our study showed a high response rate of 92.1%. Another strength is that the original instrument used was developed from both a clinical and a theoretical perspective on PCC that is seen as the model for good caring from an international perspective.[7,8,12] The model supports an application of the core principles of PC in the specific socio-cultural context of China. The literature suggests a need to see the framework based more on empirical studies from China.[22]

For peer review only - http://bmjopen.bmj.com/site/about/guidelines.xhtml

However, some limitations should be noted in this study. First, the P-CAT is primarily intended for use as a research tool to measure the presence of PCC in residential aged care facilities but the present study was conducted only in a hospital context, which may limit the ability to generalize the results to staff working in other healthcare contexts. Further psychometric evaluation of this Chinese P-CAT is needed within different settings to verify the results of this study. Second, from a psychometric evaluation the criterion-related validity was not assessed as a gold standard for the measurement of PCC in hospitals; however, no such criterion exists because of the lack of previous studies in hospital contexts. In addition, the discriminant validity of the Chinese version of P-CAT was not evaluated in our study. Future research should therefore investigate whether the questionnaire has the ability to demonstrate significant differences across hospitals and whether it is useful in discriminating between hospitals in terms of their caring culture. Third, the content validity of the P-CAT was only considered along with the procedures of cross-cultural adaptation. Fourth, only female participants were included in this study, for which reason this study may be subject to gender bias. In interpreting the results, it should be taken into account that this result does not apply to the 2.1% male nurses in China (3.5 million nurses).[39-40] Fifth, this study used a cross-sectional design, so it would be useful to conduct a longitudinal study in the future to evaluate change over time.

Palliative care in mainland China has developed slowly since the late 1980s but in recent years profesionals, patients and their families, as well as government, have become increasingly aware of its importance.[27-28,41] But even though the provision of palliative care is becoming more and more common in China, the hospice and palliative care services are not meeting the growing demand from the ageing population, and the number of patients with cancer and other life-threatening diseases is increasing.[28] The use of a reliable and valid instrument for the assessment of PCC in research and practice can serve not only to enhance

For peer review only - http://bmjopen.bmj.com/site/about/guidelines.xhtml

the quality of elderly care but also to strengthen the ongoing development of palliative care in China. Palliative care implies a person-centred approach characterized by a holistic view of the person and a belief that the person should be supported in living a life with dignity. Palliative care strives to make the whole person visible and prioritizes the patient's spiritual, existential, social, and psychological needs to the same extent as physical needs.[28,42-43] The patient version of P-CAT is used previously by the research group as a complementary assessment in the evaluation of palliative care interventions, going together with instruments for pain management and symptoms relief for the patients.[44] Another application is in the evaluation of work-place education about palliative care for staff in the nursing home context.[30] In this current project,[29] for instance, it is being used in order to investigate whether the staff's attitude to person-centredness has changed as a result of the education.

Conclusion

The results indicated that the 13-item Chinese P-CAT which is a cross-culturally adapted version of the English P-CAT shows strong internal consistency and test-retest reliability and appears to be a promising measure for evaluating staff perceptions of person-centredness in a Chinese palliative hospital setting. The results of published psychometric evaluations indicate the use of two scales in the P-CAT as was determined in this study. However, we suggest that the measurement properties of the Chinese version should be further evaluated in other samples and in different healthcare settings. An additional issue for future studies is to evaluate this instrument as a summative measurement with cut-off values for different levels of person-centred care. This can benefit healthcare staff in assessing their quality of care. The results show that P-CAT can be a useful tool for improving the quality of healthcare in terms of PCC in the Chinese context.

For peer review only - http://bmjopen.bmj.com/site/about/guidelines.xhtml

Acknowledgements

We would like to thank Ms Huang Jingjing and Ms Wu Chao, two postgraduates of the School of Public Health, Kunming Medical University, who helped us with the data collection, and Dr. Magnus Persson, Lund University for proofreading the manuscript.

Contributors

LC was responsible for the study design, data analysis and drafting of the paper. GA , PT and KM contributed to the study design and provided comments on the paper during the writing process. DE and LB provided comments on the paper during the writing process. HF, JZ and JY were responsible for the data collection. All authors have read and approved the final version of the manuscript.

Competing interests

The authors declare that there are no competing interests.

Funding

The study was supported by grants from the National Natural Science Fund of China (Grant number: 81611130077) and the Swedish Research Council (Grant number: 2015-06243).

Ethical approval and consent to participate

This study has been approved by the Ethics Committee of Kunming Medical University with the ID number KMU01. Oral and written informed consent was obtained from all persons participating in the study.

Data sharing statement

The datasets used and/or analysed during the current study are available from the corresponding author on reasonable request.

For peer review only - http://bmjopen.bmj.com/site/about/guidelines.xhtml

References

1. Pefoyo AJK, Bronskill SE, Gruneir A, et al. The increasing burden and complexity of multimorbidity. *BMC Public Health* 2015;15:415. doi:10.1186/s12889-015-1733-2
2. World population prospects: the 2012 revision. In: Department of Economic and Social Affairs (DoEaSA) PD, ed. New York: United Nations, 2013.
3. Xu J, Chen T, Liu Y, et al. Easily manageable prognostic factors in 152 Chinese elderly acute myeloid leukemia patients: A single-center retrospective study. *J Biomed Res* 2014;28(5):396-405. doi:10.7555/JBR.28.20130164
4. Liao CC, Li CR, Lee SH, et al. Social support and mortality among the aged people with major diseases or ADL disabilities in Taiwan: A national study. *Arch Gerontol Geriatr* 2015;60(2):317-21. doi: 10.1016/j.archger.2014.11.007
5. Nolan MR, Davies S, Brown J, et al. Beyond 'person-centred' care: a new vision for gerontological nursing. *J Clin Nurs* 2004;13(3a):45-53. doi: DOI 10.1111/j.1365-2702.2004.00926.x
6. Edvardsson D, Watt E, Pearce F. Patient experiences of caring and person-centredness are associated with perceived nursing care quality. *J Adv Nurs* 2017;73(1),217-227.doi:10.1111/jan.13105
7. Edvardsson D, Innes A. Measuring Person-centered Care: A Critical Comparative Review of Published Tools. *Gerontologist* 2010;50(6),834-846.doi:10.1093/geront/gnq047
8. McCormack B. A conceptual framework for person-centred practice with older people. *Int J Nurs Pract* 2003;9:202–209. DOI:10.1046/j.1440-172x.2003.00423.x
9. Martinez T, Suárez-Alvarez J, Yanguas J. Instruments for assessing Person Centered Care in Gerontology. *Psicothema* 2016;28(2):114-121.doi:10.7334/psicothema2015.263
10. Brownie S, Nancarrow S. Effects of person-centered care on residents and staff in aged-care facilities: a systematic review. *Clin Interv Aging* 2013;8:1–10.doi:10.2147/CIA.S38589
11. Edvardsson D, Sandman PO, Rasmussen B. Construction and psychometric evaluation of the Swedish language Person-centred Climate Questionnaire - staff version. *J Nurs Manag* 2009;17(7):790-5.doi: 10.1111/j.1365-2834.2009.01005.x
12. Edvardsson D, Fetherstonhaugh D, Nay R, et al. Development and initial testing of the Person-centered Care Assessment Tool (P-CAT). *Int Psychogeriatr* 2010;22(1):101-08. doi:10.1017/S1041610209990688
13. White DL, Newton-Curtis L, Lyons KS. Development and initial testing of a measure of person-directed care. *Gerontologist* 2008;48.DOI:10.1093/geront/48.supplement_1.114-123
14. Chappell NL, Colin Reid R, Gish JA. Staff-based measures of individualized care for persons with dementia in long-term care facilities. *Dementia* 2007;6(4):527-47. doi:10.1177/1471301207084372
15. Zimmerman S, Allen J, Cohen LW, et al. A Measure of Person-Centered Practices in Assisted Living: The PC-PAL. *J Am Med Dir Assoc* 2015;16(2):132-137. doi:10.1016/j.jamda.2014.07.016
16. Selan D, Jakobsson U, Condelius A. The Swedish P-CAT: modification and exploration of psychometric properties of two different versions. *Scand J Caring Sci* 2017;31(3):527-35.doi: 10.1111/scs.12366
17. Sjogren K, Lindkvist M, Sandman PO, et al. Psychometric evaluation of the Swedish version of the Person-Centered Care Assessment Tool (P-CAT). *Int Psychogeriatr* 2012;24(3):406-415.doi: 10.1017/S104161021100202x
18. Tak YR, Woo HY, You SY, et al. [Validity and Reliability of the Person-centered Care Assessment Tool in Long-term Care Facilities in Korea]. *J Korean Acad Nurs* 2015;45(3):412-419.doi: 10.4040/jkan.2015.45.3.412
19. Martinez T, Suarez-Alvarez J, Yanguas J, et al. Spanish validation of the Person-centered Care Assessment Tool (P-CAT). *Aging Ment Health* 2016;20(5):550-58.doi: 10.1080/13607863.2015.1023768
20. Rokstad AMM, Engedal K, Edvardsson D, et al. Psychometric evaluation of the Norwegian version of the Person-centred Care Assessment Tool. *Int J Nurs Pract* 2012;18(1):99-105.doi: 10.1111/j.1440-172X.2011.01998.x
21. Zhong XB, Lou VW. Person-centered care in Chinese residential care facilities: a preliminary

For peer review only - http://bmjopen.bmj.com/site/about/guidelines.xhtml

measure. *Aging Ment Health* 2013;17(8):952-58.doi:10.1080/13607863.2013.790925
22. Wang J, Wu B, Bowers BJ, et al. Person-centered dementia care in China: A bilingual literature review. *Gerontol Geriatr Med* 2019;5:1-11.doi:10.1177/2333721419844349.
23. Mao P, Xiao D, Zhang M, et al. Person-centered dementia care in eldercare institutions in Hunan province. *Journal of Nursing Science* 2016;31(21):1-4.
24. Hall S, Petkova H, Tsouros A, et al. Palliative care for older people: better practices. Copenhagen: WHO Regional Office for Europe; 2011. (Accessed October 26, 2019). http://www.euro.who.int/__data/assets/pdf_file/0017/143153/e95052.pdf
25. Davies E, Higginson IJ. Better palliative care for older people. WHO Regional Office for Europe; 2004. (Accessed October 26, 2019). http://www.euro.who.int/__data/assets/pdf_file/0009/98235/E82933.pdf
26. Price DM, Strodtman LK, Montagnini M, et al. Health professionals perceived concerns and challenges in providing palliative and end-of-life care: A qualitative analysis. *Am J Hosp Palliat Care* 2019 Apr;36(4):308-315.doi:10.1177/1049909118812193. Epub 2018 Nov 21.
27. Yin Z, Li J, Ma K, Ning X, Chen H, Fu H, Zhang H, Wang C, Bruera E, Huia D. Development of Palliative Care in China: A Tale of Three Cities. *The Oncologist* 2017;22:1362–1367.
28. Lu Y, Gu Y; Yu W. Hospice and palliative care in China: Development and challenges. *Asia-Pacific Journal of Oncology Nursing* 2018; Vol 5(1):26-32.
29. Ahlström G, Benzein E. Themed meetings about palliative care within health care (Additional file 1, The Booklet). In: Bökberg C, Behm L, Wallerstedt B & Ahlström G, editors. Evaluation of person-centeredness in nursing homes after a palliative care intervention: pre and post-test experimental design. *BMC Palliat Care* 2019;18(1):44. doi:10.1186/s12904-019-0431-8.
30. Bökberg C, Behm L, Wallerstedt B, et al. Evaluation of person-centeredness in nursing homes after a palliative care intervention: pre- and post-test experimental design. *BMC Palliat Care* 2019; 18:44. https://doi.org/10.1186/s12904-019-0431-8.
31. Guillemin F. Cross-Cultural Adaptation and Validation of Health-Status Measures. *Scand J Rheumatol* 1995;24(2):61-63. doi: Doi 10.3109/03009749509099285
32. Beaton DE, Bombardier C, Guillemin F, et al. Guidelines for the process of cross-cultural adaptation of self-report measures. *Spine* 2000;25(24):3186-91.doi: 10.1097/00007632-200012150-00014
33. Reise SP, Waller NG, Comrey AL. Factor analysis and scale revision. *Psychol Assessment* 2000;12(3):287-97. doi: 10.1037//1040-3590.12.3.287
34. Streiner DL, Norman GR, Cairney J. Health Measurement Scales: A Practical Guide to Their Development and Use. Fifth edition ed. 2014, Oxford: Oxford University Press.
35. Pett MA, Lackey NR, Sullivan JJ. Making sense of factor analysis : the use of factor analysis for instrument development in health care research: London : SAGE, cop. 2003.
36. Bland JM, Altman DG. Cronbach's alpha. *Brit Med J* 1997;314(7080):572-72. doi: DOI 10.1136/bmj.314.7080.572
37. Landis JR, Koch GG. The measurement of observer agreement for categorical data. *Biometrics* 1977;33(1):159-74. [published Online First: 1977/03/01]
38. Kline RB. Principles and Practice of Structural Equation Modeling. Fourth Edition. New York: Guilford Press 2015.
39. National Health Commission of the People's Republic of China. 2017 yearbook of health and family planning statistics in China. Beijing: China Union Medical University Press, 2017.
40. Yang J-S, Hao D-J. Dilemmas for nurses in China. Correspondence. *Lancet* 2018;392(10141):30.
41. Ning XH. Hospice and palliative care in mainland China: History, Current status and challenges. Chin Med Sci J 2018; 33(4): 199-203. doi: 10.24920/003524.
42. McCormack B, McCance TV. Development of a framework for personcentred nursing. *J Adv Nurs* 2006. https://doi.org/10.1111/j.1365-2648.2006.04042.x.
43. McCormack B, Dewing J, Breslin L, et al. Developing person-centred practice: nursing outcomes arising from changes to the care environment in residential settings for older people. *Int J Older people Nurs* 2010;5(2):93-107.doi:10.1111/j.1748-3743.2010.00216.x
44. Bökberg C, Behm L, Wallerstedt B, et al. Evaluating person-centredness for frail older persons in nursing homes before and after implementing a palliative care intervention. *Nursing Open* 2019;00:1–10. DOI: 10.1002/nop2.408

For peer review only - http://bmjopen.bmj.com/site/about/guidelines.xhtml

BMJ Open

Psychometric evaluation of the Chinese version of the Person-centred Care Assessment Tool

Journal:	*BMJ Open*
Manuscript ID	bmjopen-2019-031580.R4
Article Type:	Original research
Date Submitted by the Author:	29-Apr-2020

Complete List of Authors:	Le, Cai; Kunming Medical University in Kunming,<br>Ma, Ke; The third people's hospital of Kunming, Palliative Care<br>Tang, Pingfen; Kunming Medical University<br>Edvardsson, David; La Trobe University School of Nursing and Midwifery<br>Behm, Lina; Kristianstad University College, Faculty of Health Sciences<br>Zhang, Jie; the third people's hospital of Kunming, palliative care<br>Yang, Jiqun; the third people's hospital of Kunming<br>Fu, Haiyan; the third people's hospital of Kunming<br>Ahlström, Gerd; Lunds Universitet, Health Sciences

**Primary Subject Heading**:	Geriatric medicine
Secondary Subject Heading:	Palliative care, Nursing, Evidence based practice
Keywords:	Person-centered care, older people, PALLIATIVE CARE, instrument evaluation, factor analysis

SCHOLARONE™
Manuscripts

I, the Submitting Author has the right to grant and does grant on behalf of all authors of the Work (as defined in the below author licence), an exclusive licence and/or a non-exclusive licence for contributions from authors who are: i) UK Crown employees; ii) where BMJ has agreed a CC-BY licence shall apply, and/or iii) in accordance with the terms applicable for US Federal Government officers or employees acting as part of their official duties; on a worldwide, perpetual, irrevocable, royalty-free basis to BMJ Publishing Group Ltd ("BMJ") its licensees and where the relevant Journal is co-owned by BMJ to the co-owners of the Journal, to publish the Work in this journal and any other BMJ products and to exploit all rights, as set out in our licence.

The Submitting Author accepts and understands that any supply made under these terms is made by BMJ to the Submitting Author unless you are acting as an employee on behalf of your employer or a postgraduate student of an affiliated institution which is paying any applicable article publishing charge ("APC") for Open Access articles. Where the Submitting Author wishes to make the Work available on an Open Access basis (and intends to pay the relevant APC), the terms of reuse of such Open Access shall be governed by a Creative Commons licence – details of these licences and which Creative Commons licence will apply to this Work are set out in our licence referred to above.

Other than as permitted in any relevant BMJ Author's Self Archiving Policies, I confirm this Work has not been accepted for publication elsewhere, is not being considered for publication elsewhere and does not duplicate material already published. I confirm all authors consent to publication of this Work and authorise the granting of this licence.

For peer review only

Psychometric evaluation of the Chinese version of the Person-centred Care Assessment Tool

Running title: Evaluation of the Chinese version of P-CAT

Le Cai, PhD[1], Ke Ma, MD[2], Pingfen Tang, RN[1], David Edvardsson, PhD[3], Lina Behm, PhD[4], Jie Zhang, MD[5], Jiqun Yang, MD[6], Haiyan Fu, MD[5] and Gerd Ahlström, PhD[7]*

* Corresponding author: Professor Gerd Ahlström, Department of Health Sciences, Faculty of Medicine, Lund University, Box 157, SE-221 00 Sweden. Email: gerd.ahlstrom@med.lu.se Phone: +46 46 702 747767

[1] PhD, Professor, School of Public Health, Kunming Medical University, Cheng Gong New City, Kunming, China

[2] Affiliated physician, Division of palliative care, the Third People's Hospital of Kunming, China

[3] Professor, Austin Health/Northern Health Clinical Schools of Nursing College of Science, Health and Engineering, La Trobe University, Australia

[4] PhD, Senior Lecturer, Department of nursing educations and integrated health science, Kristianstad University, Sweden

[5] Physician, Division of palliative care, the Third People's Hospital of Kunming, China

[6] Vice-Director, Division of palliative care, the Third People's Hospital of Kunming, China

[7] PhD, Professor, Department of Health Sciences, Faculty of Medicine, Lund University, Sweden. ORCID no 0000-0001-6230-7583

Word count: 4384 words (without abstract, tables and reference list)

For peer review only - http://bmjopen.bmj.com/site/about/guidelines.xhtml

Abstract

Objective: This study aims to test a Chinese cross-cultural adaptation of the English version of the P-CAT and evaluate its psychometric properties.

Design: P-CAT was translated/back-translated using established procedures. Psychometric evaluation of the Chinese version of the Person-centred Care Assessment Tool.

Setting: Two hospitals covering urban and suburban areas of Kunming, in the Yunnan province of China.

Participants: 152 female hospital staff completed the survey

Main outcome measure(s): Construct validity and reliability, including internal consistency and test-retest reliability, were assessed among a sample of hospital staff.

Results: The factor analysis resulted in a two-component solution that consisted of two sub-scales. The corrected item-total correlations for all of the items ranged from 0.14 to 0.44, with six items that did not meet the cut-off level for item-total correlation (>0.3). The Chinese P-CAT demonstrated strong reliability, with a Cronbach's alpha of 0.91-0.94 for the scales, and a test-retest reliability coefficient of 0.88 for the overall scale scores. The ICC was 0.92 (95 % CI: 0.90- 0.95).

Conclusion: P-CAT appears to be a promising measure for evaluating staff perceptions of the person-centredness in Chinese hospital environments. The results show that P-CAT can be a useful tool for improving the quality of healthcare in terms of person-centred care in the Chinese context.

Keywords: Person-centered care, older people, palliative care, instrument evaluation, factor analysis

For peer review only

Article summary

Strengths and limitations of this study

- A previous study found the need for revision of the original P-CAT questionnaire. This study validates a translated and culturally adapted but otherwise unmodified English version of P-CAT in a Chinese context.
- High response rate (92.1%).
- Convenience sampling method may limit the ability to generalize the results.
- The Chinese P-CAT has been tested only in this geriatric hospital setting.

For peer review only

For peer review only - http://bmjopen.bmj.com/site/about/guidelines.xhtml

Introduction

Population ageing is occurring globally, and multi-morbidity is highly prevalent, particularly in older people, who are generally frequent healthcare users.[1] The Chinese population is one of the fastest-ageing populations in the world, and the number of people aged 60 years and older is expected to rise from 193 million (13.9%) in 2013 to 454 million (32.8%) in 2050.[2] This rapid increase in the number of older people in China is reflected in the increasing number of older people admitted to hospitals. The National Health Services survey of China shows that the hospitalization rate of people aged 65 years and older increased significantly from 6.1% to 19.9% during the period 2003–2013,[3] and chronic diseases occur more frequently in older people and are more difficult to cure when multiple diseases coexist.[4] Thus, the healthcare system in China is facing great challenges due to the aging population who require high-quality care from a wide range of multiple care professionals.

Person-centred care (PCC) is now regarded as a valuable approach to use when it comes to improving long-term care for older people,[5] and the quality of care is often described in terms of its being person-centred.[6-8] The growing interest in applying PCC is accompanied by an interest in developing instruments, commonly questionnaires answered by staff as a proxy for the ill person.[7,9] The core values of PCC are the social relationship between the staff and the ill person and the close family, characterized by respect for the person's will, mutuality, openness, honesty, and sympathetic presence.[8] Even though there is a range of terms in the literature for describing PCC, all imply a similar philosophy of looking at the person's needs instead of a disease.[7] A review shows that person-centred interventions are multifactorial and that the complexity of the interventions can explain the range of existing outcome variables.[10]

To date, a number of instruments have been developed to assess the PCC in residential care facilities for older people, and these instruments are mainly based on the opinions of staff working in such facilities. The instruments are the English Person-centred Climate

For peer review only

Questionnaire – Staff version (PCQ-S),[11] the Person-centred Care Assessment Tool (P-CAT),[12] the Staff Assessment Person-Directed Care tool (PDC),[13] the Individualized Care tool (IC)[14] and the Staff Person-Centred Practices in Assisted Living tool (Staff PC-PAL).[15] Of these, the tool most commonly used internationally, with established credibility, is the P-CAT.[7,9] The P-CAT questionnaire was designed by Edvardsson and colleagues[12] and involves a caregiver-based self-report assessment scale used to measure the extent to which the staff agree that a number of pre-defined characteristics/processes of PCC are in place within their team, unit or nursing home. The P-CAT is easy to use, with only 13 items. It has a process-focus, as evidenced in items connected with work routines, residents' life stories, assessment of personal needs, and discussion concerning best care for the residents. It has been validated and used in English,[12] Swedish,[16-17] Korean,[18] Spanish,[19] Norwegian,[20] and Chinese.[21]

A recently published literature review [22] indicates that the studies of PCC were predominantly on populations in Western Europe and the USA. However, the increasing proportion of older people implies an urgent need for the implementation of PCC for the purpose of improving the quality of care. The study of Zhong and colleagues[21] found that the P-CAT was a good tool to adapt for use in the Chinese context. A 24-item Chinese version of the original English 13-item P-CAT was developed and later reduced to 15 items as a result of psychometric validation on staff. This modified version of P-CAT was changed to such an extent as to make it impossible to compare the results with results obtained by use of the original English version. Two items were removed and four items were added, generating three scales.[21] This Chinese version was validated on staff working in residential care for patients with dementia because P-CAT was originally intended for use as a research instrument to measure the presence of PCC in this context.[12,23]

Palliative care is provided in a person-centred environment, where the older person and the family feel welcome, feel that they are seen, feel that they are involved by the staff.

For peer review only - http://bmjopen.bmj.com/site/about/guidelines.xhtml

This type of supportive care has been developed in hospice settings by specialists in the care of patients dying from incurable cancer, and older persons dying from other diseases do not have the same access to palliative care.[24-25] The ageing people with multiple morbidities have complex care needs and multiple symptoms in their last period of life, which requires both geriatric and palliative expertise. However, staff need education about palliative care to be able to provide person-centered care integrated with a focus on symptom management and quality of life for the older people and their family.[24,26]

To date, palliative care is widely provided in Western countries, but access to such service is extremely limited in mainland China,[27] especially outside oncology departments. Since palliative care is still in its initial stage and not enough in respect of the aging population and the number of patients with life-threatening diseases that are increasing in China.[27-28] Therefore, the intervention project entitled "Palliative care for elderly people with chronic illnesses: a comparative study between Sweden and China" was initiated. The purpose of the project is to improve palliative care through work-place education in geriatric hospital care.[29] In order to promote the improvement of the quality of care through the implementation of person-centred palliative care in a Chinese healthcare context, further testing and cross-validation of the original P-CAT was performed on staff in Chinese geriatric palliative hospital settings. A short instrument as P-CAT was preferable in this study due to the need for combinations with additional measurements. It is necessary to ensure that this outcome measure is applicable in the evaluation of educational intervention for staff working in a geriatric hospital. Also, a psychometric evaluation of the original 13 items of the English version means that it should be possible to compare results from Sweden in forthcoming studies within the current intervention project involving Sweden[30] and China.

In China, PCC remains a poorly understood approach that is not generally applied in geriatric services, and one of the possible barriers is the absence of a dependable and valid

For peer review only

For peer review only - http://bmjopen.bmj.com/site/about/guidelines.xhtml

instrument to measure person-centred care. Therefore, the aim of this study was to accomplish a cultural adaptation of the original English version of the P-CAT for Chinese nurses as well as evaluate the psychometric properties of the newly translated Chinese version applied in geriatric palliative care.

Methods

This study tested and evaluated the Chinese version of the P-CAT in Mandarin, the most common language in China, on nurses in a hospital-based palliative care setting. This psychometric study is part of the intervention project "Palliative care for elderly people with chronic illnesses: a comparative study between Sweden and China".

The original English instrument

P-CAT measures the extent to which staff engaged in long-term care of the elderly consider their settings to be person-centred. The English P-CAT questionnaire has been validated in an Australian sample of nursing home staff and was shown to have satisfactory psychometric properties.[12] The self-reported questionnaire exists in both a staff version and a version for older persons. In this study, the version for staff was used. It comprises 13 items covering three sub-scales (the Extent of personalizing care, the Amount of organizational support, and the Degree of environmental accessibility). The Extent of personalizing care was measured through items 1-7, Amount of organizational support was measured through items 8-11, and Degree of environmental accessibility was measured through items 12-13. A 5-point Likert-type scale ranging from 1 (strongly disagree) to 5 (strongly agree) was used for scoring. Items 8–12 were negatively worded and the responses were reverse-scored before analysing the data and calculating a total score. Aggregated scores were calculated by means of simple sum scores at the sub-scale and total scale levels. The total score ranged from 13 to 65 and a higher score indicated a higher degree of person-centredness.

For peer review only - http://bmjopen.bmj.com/site/about/guidelines.xhtml

Translation and cross-cultural adaptation of the P-CAT

The process of translation and cross-cultural adaptation followed International test commission guidelines.[31,32] Three native Chinese speakers produced independent forward translations from English to Chinese; two had a Public Health degree and the third was a senior nurse who had worked in a department of palliative care for over ten years. First, the translators agreed upon a consensus version after discussing the different versions. Then this consensus version was back-translated into English by two new translators who were bilingual and had no knowledge of the procedures of the forward translation. After that, any discrepancies were resolved by an expert committee composed of all the translators, two geriatric physicians, one senior nurse and two university professors. The back-translated version was modified by comparing it with the original version. The expert committee culturally adapted a few phrases to fit the Chinese setting, resulting in a pre-final version. The final version of the Chinese P-CAT was generated after pre-testing for face-validity, involving ten female nurses from a municipal hospital in Kunming, with a mean age of 31.2 years (SD ±8.3). The pre-testing resulted in no changes, the final Chinese version of the P-CAT is shown in the Appendix. The ten staff members who participated in the face-validity testing of the pre-final version did not participate in the later study.

Sample, participants and data collection

The sample was selected to cover hospital nurses working with palliative care both in urban and suburban areas of Kunming, the capital of the Yunnan province in southwest China. Two municipal hospitals in Kunming were chosen through a convenience sampling method: one hospital from the Wu Hua district (an urban area of Kunming) and one from the Guan Du district (a suburban area of Kunming).

For peer review only - http://bmjopen.bmj.com/site/about/guidelines.xhtml

Patients who were admitted to the sampled hospitals had multiple, complex, and overlapping chronic diseases, and most patients were aged 60 years or older. The following criterion for inclusion was used: municipal level hospital with a department of palliative care.

All nurses (n=165) on duty (all shifts on the day of the data collection) at the palliative care departments of the two hospitals were deemed to be eligible for participation and were asked whether they would be willing to participate in the study. The sample size was determined by the number of available nurses working at the palliative care departments.

Finally, 152 nurses gave their informed consent and completed the Chinese version of the P-CAT questionnaire. The participants were asked to take part in both the test and retest assessments, and all 152 participants completed the first and the follow-up test. This gives an overall response rate of 92.1%. The retest was conducted between one and two weeks after the initial test.

The questionnaires were distributed by two graduate students to all participants, and the completed questionnaires were then anonymously collected on site. In addition to the P-CAT scale, demographic characteristics of the participants were also collected by means of a questionnaire that assessed age, gender, level of education, ethnicity and position.

Psychometric evaluation

Only one item in one questionnaire had a missing value and it was replaced with the mean value of the item for the entire group.[33] An exploratory factor analysis (EFA) with extraction of principal components by both varimax and oblique rotation was used to evaluate construct validity. After either direct varimax rotating or oblique rotating, components in the structural matrix comprised the same numbers of items and loadings, indicating that no difference was found between these two methods in the analysis, and thus only the results of the analysis with varimax rotation are presented. Bartlett's test of sphericity and the Keiser-Meyer-Olkin (KMO)

For peer review only

measure were applied in order to check the appropriateness of the factor analysis. In order to assess whether the correlation between items was adequate based on a criterion of $p< 0.0001$, Bartlett'stest of sphericity was used, and a criterion of KMO $\geqslant 0.70$ was used to indicate sample adequacy. When the Kaiser criterion of eigenvalues was $\geqslant 1$, factors were extracted. A cut-off of 0.50 was applied to determine item loadings on each individual component.[34]

The internal consistency and test-retest reliability were evaluated. The internal consistency for the total and sub-scale scores was estimated using Cronbach's alpha coefficient, and Cronbach's alpha ≥ 0.50 was used as acceptable internal consistency reliability.[35] The item-total correlations were also computed, and the cut-off score for acceptable item-total correlations was set to between 0.3 and 0.8 to ensure moderate correlation and avoid item redundancy.[33] Test-retest reliability was examined with the intra-class correlation (ICC) and Pearson's correlation coefficient (r), where an ICC >0.80 was taken to indicate satisfactory reliability.[36] The paired t-test (2-tailed) was used to verify whether the mean scores from the test and retest differed.

The results of the principal component analysis (PCA) indicate that three factors with eigenvalues above 1 produced a three-component rotated solution that explained 60.2% of the total variance in the data. After direct varimax orthogonal rotating, the structure matrix showed that the first component comprised seven items, the second comprised four items, and the third comprised two items. With regard to the internal consistency reliability, the Cronbach's alpha coefficient of the 13-item Chinese P-CAT was 0.65 for the total scale, 0.67 for the first sub-scale, 0.85 for the second sub-scale and 0.42 for the third sub-scale. Since sub-scale three failed to meet the reliability cut-off of 0.5, the analysis was re-run by forcing the EFA into 2 factors.

The two-factor model was also evaluated by means of a Confirmatory Factor Analysis (CFA) within the framework of Structural Equation Modelling (SEM).[37] The goodness of fit was evaluated using indices of the Normed Fit Index (NFI), the Comparative Fit Index (CFI) and

For peer review only - http://bmjopen.bmj.com/site/about/guidelines.xhtml

the Root Mean Square Error of Approximation (RMSEA). The results indicated that the goodness of fit of the questionnaire was 0.91 for the NFI, 0.91 for the CFI and 0.73 for the RMSEA. Thus, the CFA supported the exploratory findings. Statistical significance was determined based on two-tailed P values of <0.05. All data analyses were performed using SPSS 22.0 software, and the two-factor model was evaluated by means of a CFA within the framework of SEM with SPSS 22.0 under the Package 'Analysis of Moment Structures' (AMOS).

Patient and Public Involvement

Patients, relatives, and staff from palliative care were involved in the development of work-place education for staff and informed by patients' priorities, experience, and preferences.[29] It is planned to disseminate the results to staff at meetings about palliative care.

Results

Demographic characteristics of the study group

As described in Table 1, the sample consisted of only female nurses, with a mean age of 30.7 years (SD ±8.7, range 19-53 years). In total, 25.7% of the nurses had a Bachelor's degree or higher. More than one-fourth of the participants reported a minority ethnicity.

For peer review only - http://bmjopen.bmj.com/site/about/guidelines.xhtml

Table 1 Demographic characteristics of the study sample (n=152)

**Characteristics**	**n (%)**
Gender	
Female	152 (100.0)
Male	0 (0.0)
Age (years)	
18-30	96 (63.2)
31-39	22 (14.5)
⩾40	34 (22.4)
Level of education	
High school	14 (9.2)
Secondary school	39 (25.7)
Junior college	60 (39.5)
Bachelor	39 (25.7)
Ethnicity	
Han	111 (73.0)
Minorities	41 (27.0)

For peer review only

Construct validity Bartlett's test of sphericity was significant (754.99, $p< 0.0001$) and the KMO measure were satisfactory (0.78), which indicates that correlations between items were sufficient to conduct a principal component analysis (PCA). As shown in Table 2, the results of the PCA indicate that two factors with eigenvalues above 1 produced a two-component rotated solution that explained 50.4% of the total variance in the data. After direct varimax orthogonal rotating, the structural matrix showed that the first component comprised nine items (loadings between 0.16 and 0.79) and the second comprised four items (loadings between 0.71 and 0.89). The two sub-scales were labelled as (1) Extent of personalizing care and (2) Amount of organizational and environmental support.

For peer review only - http://bmjopen.bmj.com/site/about/guidelines.xhtml

Table 2 Rotated component matrix for PCA of the 2-factor Chinese P-CAT

Item number	Item content	Factor loadings	
		**Sub-scale 1: Extent of personalizing care**	**Sub-scale 2: Amount of organizational and environmental support**
1	We often discuss how to provide person-centred care.	**0.73**	0.008
2	We have formal team meetings to discuss residents' care.	**0.79**	0.045
3	The life history of the residents is formally used in the care plans we use.	**0.76**	0.028
4	The quality of the interaction between staff and residents is more important than getting the tasks done.	**0.72**	-0.087
5	We are free to alter work routines based on residents' preferences.	**0.67**	0.117
6	Residents are offered the opportunity to be involved in individualized everyday activities.	**0.54**	-0.109
7	Assessment of residents' needs is undertaken on a daily basis.	**0.17**	0.26
8	I simply do not have the time to provide person-centred care.	-0.208	**0.78**
9	The environment feels chaotic.	-0.096	**0.89**

For peer review only - http://bmjopen.bmj.com/site/about/guidelines.xhtml

10	We have to get the work done before we can worry about a homelike environment.	**0.64**	-0.364
11	The organization prevents me from providing person-centred care.	-0.094	**0.89**
12	It is hard for residents in the facility to find their way around.	-0.036	**0.71**
13	Residents are able to access outside space as they wish.	**0.35**	-0.087
Total variance explained (%)	50.4 (total 2 sub-scales)	30.18	20.24
Cronbach's alpha	0.92 (total 13 items)	0.94	0.91

Note: Bold number means included in the scale

Reliability

With regard to the internal consistency reliability, the Cronbach's alpha coefficient of the 13-item Chinese P-CAT was 0.92 for the total scale, 0.94 for the sub-scale Extent of personalized care, and 0.91 for the sub-scale Amount of organizational and environmental support sub-scale (Table 2), indicating that the overall internal consistency reliability was strong. The corrected item-total correlations for all items ranged from 0.14 to 0.44 (Table 3), with six items (items 6, 7, 9, 11, 12 and 13) failing to meet the commonly suggested cut-off level for item-total correlation (>0.3).

For peer review only

For peer review only - http://bmjopen.bmj.com/site/about/guidelines.xhtml

Table 3 Item performance and reliability test of the Chinese P-CAT

Item number	Item content	Mean±SD	Corrected item- total correction	Cronbach's alpha if item deleted
1	We often discuss how to provide person-centred care.	4.19±0.70	0.36	0.62
2	We have formal team meetings to discuss residents' care.	4.32±0.66	0.44	0.61
3	The life history of the residents is formally used in the care plans we use.	4.24±0.73	0.41	0.62
4	The quality of the interaction between staff and residents is more important than getting the tasks done.	4.50±0.65	0.32	0.63
5	We are free to alter work routines based on residents' preferences.	4.20±0.77	0.42	0.61
6	Residents are offered the opportunity to be involved in individualized everyday activities.	3.86±0.82	0.23	0.64
7	Assessment of residents' needs is undertaken on a daily basis.	2.30±1.10	0.19	0.65
8	I simply do not have the time to provide person-centred care.	3.89±0.82	0.35	0.62
9	The environment feels chaotic.	4.11±1.00	0.15	0.65
10	We have to get the work done before we can worry about a homelike environment.	1.74±0.62	0.37	0.62
11	The organization prevents me from providing person-centred care.	4.14±0.92	0.17	0.65
12	It is hard for residents in the facility to find their way around.	2.22±0.88	0.26	0.64

For peer review only

13	Residents are able to access outside space as they wish.	3.89±0.82	0.14	0.65

Table 4 shows the results from the test-retest reliability assessment of the Chinese P-CAT. The results of Pearson's correlation coefficient analysis indicated that the Chinese P-CAT had a high correlation between the test and retest on all scale levels: on the sub-scales Extent of personalizing care (r=0.90, P<0.01) and Amount of organizational and environmental support (r=0.88, P<0.01) and on the overall scale (r=0.88, P<0.01). A paired t-test also confirmed that there was no significant difference between the mean scores of the P-CAT on the test and retest (P>0.05). The ICC of the total score between the test and retest was 0.92, providing further support for stating that the scale had satisfactory test-retest reliability.

Table 4 Test-retest reliability of the Chinese P-CAT

**Scale dimension**	**1st test (Mean±SD)**	**2nd test (Mean±SD)**	**p**	**Pearson correlation coefficient (r)**	**95% CI**	**ICC (95 % CI)**
Extent of personalizing care	33.35±3.59	33.13±3.33	0.09	0.90	(0.83, 0.94)	0.94 (0.92, 0.96)
Amount of organizational and environmental support	14.41±1.72	14.34±1.74	0.34	0.88	(0.76, 0.89)	0.91 (0.87, 0.93)
Overall scale	47.76±3.70	47.46±3.65	0.07	0.88	(0.78, 0.92)	0.92 (0.90, 0.95)

Note: p= two-tailed p-values of paired t-test; ICC: intra-class correlation

For peer review only

Discussion

In this study, the English P-CAT was culturally adapted into a Chinese version. During the translation of the English P-CAT into Chinese, a few minor cultural discrepancies were encountered, and two items of the P-CAT have been modified accordingly. In order to use words closer to the Chinese cultural context and more applicable to a hospital environment, the term ‘resident’ was replaced by ‘patient’ and the term ‘free’ was replaced by ‘flexible’. After cross-cultural adaptation, the pre-testing of the Chinese version of the P-CAT revealed that the items were clearly understood.

This Chinese version of the P-CAT showed a different sub-scale structure as compared to the original English scale, i.e. a structure with three sub-scales (Extent of personalizing care, Amount of organizational support and Degree of environmental accessibility) consisting of 13 items. On the other hand it showed the same sub-scale structure as the Norwegian scale, i.e. a structure which had two sub-scales (Extent of personalizing care and Amount of organizational and environmental support). The 13 items were mostly in concordance with the Norwegian version whereas only item 10 was connected to the first sub-scale.[20] The 2-factor structure has also been confirmed in the Swedish version of the P-CAT based on a large cross-sectional sample of 1,465 staff members from 195 residential elderly care units[17] and in a more recent study based on 142 staff working in residential elderly care units and 182 staff working at healthcare centres or home care centres and in social services.[16] Moreover, in this study the Chinese P-CAT showed strong internal consistency reliability with a Cronbach’s alpha of 0.92 for the total scale, indicating that the Chinese P-CAT it matched well with the characteristics and structure of the Norwegian version. However, the different sub-scale structures of this Chinese version of the P-CAT as compared to the original English scale may reflect a difference in sample characteristics of staff and the cultural context. The test-retest reliability assessed by ICC was excellent for overall Chinese P-CAT score (0.92) and both sub-scales

For peer review only - http://bmjopen.bmj.com/site/about/guidelines.xhtml

(0.94 and 0.91), and the excellent test-retest reliability of the Chinese P-CAT was further evidenced through a Pearson's correlation coefficient (r) of 0.90 for the total scale, 0.88 for the Extent of personalizing care sub-scale and 0.88 for the Amount of organizational and environmental support sub-scale. The results of Pearson's correlation coefficient (r) for the total scale in the Chinese version were higher than in the English (0.66),[12] Swedish (0.75),[16-17] Norwegian (0.83)[20] and previous Chinese (0.68)[21] versions, which indicates that this Chinese P-CAT may have a stronger test-retest reliability than other-language versions of the P-CAT.

In this Mandarin version of the Chinese P-CAT, six items (6, 7, 9, 11, 12 and 13) did not meet the commonly suggested cut-off level for item-total correlation (>0.3). In the previous Chinese version of the P-CAT,[21] items 7 and 9 were deleted due to the low value of factor loading (<0.3), and four newly developed items were added to the scale.[21] Item 7 in this study also had a lower value of factor loading than is recommended. However, we decided to include this item in the sub-scale 1, in agreement with the original English version, although this item had a slightly higher value of factor loading in the case of factor 2. Because these two Chinese versions of the P-CAT were evaluated in different healthcare settings (hospital environments and residential care facilities), there is no firm consensus regarding the psychometric properties of the Chinese P-CAT, and the findings suggest that the Chinese version requires further investigation and analysis with other samples and in other settings.

In contrast to the low response rate of 21% in the development study of the original English version P-CAT,[12] our study showed a high response rate of 92.1%. Another strength is that the original instrument used was developed from both a clinical and a theoretical perspective on PCC that is seen as the model for good caring from an international perspective.[7,8,12] The model supports an application of the core principles of PC in the specific socio-cultural context of China. The literature suggests a need to see the framework based more on empirical studies from China.[22]

For peer review only - http://bmjopen.bmj.com/site/about/guidelines.xhtml

However, some limitations should be noted in this study. First, the P-CAT is primarily intended for use as a research tool to measure the presence of PCC in residential aged care facilities but the present study was conducted only in a hospital context, which may limit the ability to generalize the results to staff working in other healthcare contexts. Further psychometric evaluation of this Chinese P-CAT is needed within different settings to verify the results of this study. Second, from a psychometric evaluation the criterion-related validity was not assessed as a gold standard for the measurement of PCC in hospitals; however, no such criterion exists because of the lack of previous studies in hospital contexts. In addition, the discriminant validity of the Chinese version of P-CAT was not evaluated in our study. Future research should therefore investigate whether the questionnaire has the ability to demonstrate significant differences across hospitals and whether it is useful in discriminating between hospitals in terms of their caring culture. Third, the content validity of the P-CAT was only considered along with the procedures of cross-cultural adaptation. Fourth, only female participants were included in this study, for which reason this study may be subject to gender bias. In interpreting the results, it should be taken into account that this result does not apply to the 2.1% male nurses in China (3.5 million nurses).[38-39] Fifth, this study used a cross-sectional design, so it would be useful to conduct a longitudinal study in the future to evaluate change over time Sixth, the sample size despite a high overall response rate (92.1%) was limited to 152 nurses. We accepted this sample size for the possibility to compare results from the target group (nurses in palliative care) within the ongoing intervention project involving Sweden[30] and China. The sample size recommendations for factor analysis vary in the literature[40-41], commonly 5:1[41-42], or 10:1[43] sample-to-item ratios. Others have suggested 100 as an absolute minimum sample size.[40-41]

Palliative care in mainland China has developed slowly since the late 1980s but in recent years professionals, patients and their families, as well as government, have become

For peer review only

increasingly aware of its importance.[27-28,44] But even though the provision of palliative care is becoming more and more common in China, the hospice and palliative care services are not meeting the growing demand from the ageing population, and the number of patients with cancer and other life-threatening diseases is increasing.[28] The use of a reliable and valid instrument for the assessment of PCC in research and practice can serve not only to enhance the quality of elderly care but also to strengthen the ongoing development of palliative care in China. Palliative care implies a person-centred approach characterized by a holistic view of the person and a belief that the person should be supported in living a life with dignity. Palliative care strives to make the whole person visible and prioritizes the patient's spiritual, existential, social, and psychological needs to the same extent as physical needs.[28,45-46] The patient version of P-CAT is used previously by the research group as a complementary assessment in the evaluation of palliative care interventions.[47] Another application is in the evaluation of work-place education about palliative care for staff in the nursing home context.[30] In this current project,[29-30] for instance, it is being used in order to investigate whether the staff's attitude to person-centredness has changed as a result of the education.

Conclusion

The results indicated that the 13-item Chinese P-CAT which is a cross-culturally adapted version of the English P-CAT shows strong internal consistency and test-retest reliability and appears to be a promising measure for evaluating staff perceptions of person-centredness in a Chinese palliative hospital setting. The results of published psychometric evaluations indicate the use of two scales in the P-CAT as was determined in this study. However, we suggest that the measurement properties of the Chinese version should be further evaluated in other samples and in different healthcare settings. An additional issue for future studies is to evaluate this instrument as a summative measurement with cut-off values for different levels of person-

For peer review only - http://bmjopen.bmj.com/site/about/guidelines.xhtml

centred care. This can benefit healthcare staff in assessing their quality of care. The results show that P-CAT can be a useful tool for improving the quality of healthcare in terms of PCC in the Chinese context.

Acknowledgements

We would like to thank Ms Huang Jingjing and Ms Wu Chao, two postgraduates of the School of Public Health, Kunming Medical University, who helped us with the data collection, and Dr. Magnus Persson, Lund University for proofreading the manuscript.

Contributors

LC was responsible for the study design, data analysis and drafting of the paper. GA , PT and KM contributed to the study design and provided comments on the paper during the writing process. DE and LB provided comments on the paper during the writing process. HF, JZ and JY were responsible for the data collection. All authors have read and approved the final version of the manuscript.

Competing interests

The authors declare that there are no competing interests.

Funding

The study was supported by grants from the National Natural Science Fund of China (Grant number: 81611130077) and the Swedish Research Council (Grant number: 2015-06243).

Ethical approval and consent to participate

This study has been approved by the Ethics Committee of Kunming Medical University with

For peer review only

the ID number KMU01. Oral and written informed consent was obtained from all persons participating in the study.

Data sharing statement

The datasets used and/or analysed during the current study are available from the corresponding author on reasonable request.

References

1. Pefoyo AJK, Bronskill SE, Gruneir A, et al. The increasing burden and complexity of multimorbidity. *BMC Public Health* 2015;15:415. doi:10.1186/s12889-015-1733-2
2. World population prospects: the 2012 revision. In: Department of Economic and Social Affairs (DoEaSA) PD, ed. New York: United Nations, 2013.
3. Xu J, Chen T, Liu Y, et al. Easily manageable prognostic factors in 152 Chinese elderly acute myeloid leukemia patients: A single-center retrospective study. *J Biomed Res* 2014;28(5):396-405. doi:10.7555/JBR.28.20130164
4. Liao CC, Li CR, Lee SH, et al. Social support and mortality among the aged people with major diseases or ADL disabilities in Taiwan: A national study. *Arch Gerontol Geriatr* 2015;60(2):317-21. doi: 10.1016/j.archger.2014.11.007
5. Nolan MR, Davies S, Brown J, et al. Beyond 'person-centred' care: a new vision for gerontological nursing. *J Clin Nurs* 2004;13(3a):45-53. doi: DOI 10.1111/j.1365-2702.2004.00926.x
6. Edvardsson D, Watt E, Pearce F. Patient experiences of caring and person-centredness are associated with perceived nursing care quality. *J Adv Nurs* 2017;73(1),217-227.doi:10.1111/jan.13105
7. Edvardsson D, Innes A. Measuring Person-centered Care: A Critical Comparative Review of Published Tools. *Gerontologist* 2010;50(6),834-846.doi:10.1093/geront/gnq047
8. McCormack B. A conceptual framework for person-centred practice with older people. *Int J Nurs Pract* 2003;9:202–209. DOI:10.1046/j.1440-172x.2003.00423.x
9. Martinez T, Suárez-Alvarez J, Yanguas J. Instruments for assessing Person Centered Care in Gerontology. *Psicothema* 2016;28(2):114-121.doi:10.7334/psicothema2015.263
10. Brownie S, Nancarrow S. Effects of person-centered care on residents and staff in aged-care facilities: a systematic review. *Clin Interv Aging* 2013;8:1–10.doi:10.2147/CIA.S38589
11. Edvardsson D, Sandman PO, Rasmussen B. Construction and psychometric evaluation of the Swedish language Person-centred Climate Questionnaire - staff version. *J Nurs Manag* 2009;17(7):790-5.doi: 10.1111/j.1365-2834.2009.01005.x
12. Edvardsson D, Fetherstonhaugh D, Nay R, et al. Development and initial testing of the Person-centered Care Assessment Tool (P-CAT). *Int Psychogeriatr* 2010;22(1):101-08. doi:10.1017/S1041610209990688
13. White DL, Newton-Curtis L, Lyons KS. Development and initial testing of a measure of person-directed care. *Gerontologist* 2008;48.DOI:10.1093/geront/48.supplement_1.114-123
14. Chappell NL, Colin Reid R, Gish JA. Staff-based measures of individualized care for persons with dementia in long-term care facilities. *Dementia* 2007;6(4):527-47. doi:10.1177/1471301207084372
15. Zimmerman S, Allen J, Cohen LW, et al. A Measure of Person-Centered Practices in Assisted Living: The PC-PAL. *J Am Med Dir Assoc* 2015;16(2):132-137.

For peer review only - http://bmjopen.bmj.com/site/about/guidelines.xhtml

doi:10.1016/j.jamda.2014.07.016
16. Selan D, Jakobsson U, Condelius A. The Swedish P-CAT: modification and exploration of psychometric properties of two different versions. *Scand J Caring Sci* 2017;31(3):527-35.doi: 10.1111/scs.12366
17. Sjogren K, Lindkvist M, Sandman PO, et al. Psychometric evaluation of the Swedish version of the Person-Centered Care Assessment Tool (P-CAT). *Int Psychogeriatr* 2012;24(3):406-415.doi: 10.1017/S104161021100202x
18. Tak YR, Woo HY, You SY, et al. [Validity and Reliability of the Person-centered Care Assessment Tool in Long-term Care Facilities in Korea]. *J Korean Acad Nurs* 2015;45(3):412-419.doi: 10.4040/jkan.2015.45.3.412
19. Martinez T, Suarez-Alvarez J, Yanguas J, et al. Spanish validation of the Person-centered Care Assessment Tool (P-CAT). *Aging Ment Health* 2016;20(5):550-58.doi: 10.1080/13607863.2015.1023768
20. Rokstad AMM, Engedal K, Edvardsson D, et al. Psychometric evaluation of the Norwegian version of the Person-centred Care Assessment Tool. *Int J Nurs Pract* 2012;18(1):99-105.doi: 10.1111/j.1440-172X.2011.01998.x
21. Zhong XB, Lou VW. Person-centered care in Chinese residential care facilities: a preliminary measure. *Aging Ment Health* 2013;17(8):952-58.doi:10.1080/13607863.2013.790925
22. Wang J, Wu B, Bowers BJ, et al. Person-centered dementia care in China: A bilingual literature review. *Gerontol Geriatr Med* 2019;5:1-11.doi:10.1177/2333721419844349.
23. Mao P, Xiao D, Zhang M, et al. Person-centered dementia care in eldercare institutions in Hunan province. *Journal of Nursing Science* 2016;31(21):1-4.
24. Hall S, Petkova H, Tsouros A, et al. Palliative care for older people: better practices. Copenhagen: WHO Regional Office for Europe; 2011. (Accessed October 26, 2019). http://www.euro.who.int/__data/assets/pdf_file/0017/143153/e95052.pdf
25. Davies E, Higginson IJ. Better palliative care for older people. WHO Regional Office for Europe; 2004. (Accessed October 26, 2019). http://www.euro.who.int/__data/assets/pdf_file/0009/98235/E82933.pdf
26. Price DM, Strodtman LK, Montagnini M, et al. Health professionals perceived concerns and challenges in providing palliative and end-of-life care: A qualitative analysis. *Am J Hosp Palliat Care* 2019 Apr;36(4):308-315.doi:10.1177/1049909118812193. Epub 2018 Nov 21.
27. Yin Z, Li J, Ma K, Ning X, Chen H, Fu H, Zhang H, Wang C, Bruera E, Huia D. Development of Palliative Care in China: A Tale of Three Cities. *The Oncologist* 2017;22:1362–1367.
28. Lu Y, Gu Y; Yu W. Hospice and palliative care in China: Development and challenges. *Asia-Pacific Journal of Oncology Nursing* 2018; Vol 5(1):26-32.
29. Ahlström G, Benzein E. Themed meetings about palliative care within health care (Additional file 1, The Booklet). In: Bökberg C, Behm L, Wallerstedt B & Ahlström G, editors. Evaluation of person-centeredness in nursing homes after a palliative care intervention: pre and post-test experimental design. *BMC Palliat Care* 2019;18(1):44. doi:10.1186/s12904-019-0431-8.
30. Bökberg C, Behm L, Wallerstedt B, et al. Evaluation of person-centeredness in nursing homes after a palliative care intervention: pre- and post-test experimental design. *BMC Palliat Care* 2019; 18:44. https://doi.org/10.1186/s12904-019-0431-8.
31. Guillemin F. Cross-Cultural Adaptation and Validation of Health-Status Measures. *Scand J Rheumatol* 1995;24(2):61-63. doi: Doi 10.3109/03009749509099285
32. Beaton DE, Bombardier C, Guillemin F, et al. Guidelines for the process of cross-cultural adaptation of self-report measures. *Spine* 2000;25(24):3186-91.doi: 10.1097/00007632-200012150-00014
33. Streiner DL, Norman GR, Cairney J. Health Measurement Scales: A Practical Guide to Their Development and Use. Fifth edition ed. 2014, Oxford: Oxford University Press.
34. Pett MA, Lackey NR, Sullivan JJ. Making sense of factor analysis : the use of factor analysis for instrument development in health care research: London : SAGE, cop. 2003.
35. Bland JM, Altman DG. Cronbach's alpha. *Brit Med J* 1997;314(7080):572-72. doi: DOI 10.1136/bmj.314.7080.572
36. Landis JR, Koch GG. The measurement of observer agreement for categorical data. *Biometrics* 1977;33(1):159-74. [published Online First: 1977/03/01]
37. Kline RB. Principles and Practice of Structural Equation Modeling. Fourth Edition. New York:

For peer review only - http://bmjopen.bmj.com/site/about/guidelines.xhtml

Guilford Press 2015.
38. National Health Commission of the People's Republic of China. 2017 yearbook of health and family planning statistics in China. Beijing: China Union Medical University Press, 2017.
39. Yang J-S, Hao D-J. Dilemmas for nurses in China. Correspondence. *Lancet* 2018;392(10141):30.
40. Westergren A, Melgaard D. The minimal eating observation form-II Danish version: Psychometric and metrological aspects. *J Nurs Meas* 2020;28(1):168-184.
41. MacCallum RC, Widaman K.F, Zhang S, Hong S. Sample size in factor analysis. *Psychological Methods* 1999;4(1):84-99.
42. Suhr, D. (2006). Exploratory or confirmatory factor analysis? Paper presented at the SAS Users Group International Conference, Cary, NC.44.
43. Everitt, B. S. (1975). Multivariate analysis: The need for data, and other problems. *Br J Psychiatry*, 126, 237–240. https://doi.org/10.1192/bjp.126.3.237
44. Ning XH. Hospice and palliative care in mainland China: History, Current status and challenges. Chin Med Sci J 2018; 33(4): 199-203. doi: 10.24920/003524.'
45. McCormack B, McCance TV. Development of a framework for personcentred nursing. *J Adv Nurs* 2006. https://doi.org/10.1111/j.1365-2648.2006.04042.x.
46. McCormack B, Dewing J, Breslin L, et al. Developing person-centred practice: nursing outcomes arising from changes to the care environment in residential settings for older people. *Int J Older people Nurs* 2010;5(2):93-107.doi:10.1111/j.1748-3743.2010.00216.x
47. Bökberg C, Behm L, Wallerstedt B, et al. Evaluating person-centredness for frail older persons in nursing homes before and after implementing a palliative care intervention. *Nursing Open* 2019;00:1–10. DOI: 10.1002/nop2.408

For peer review only

蔡乐 昆明医科大学

Appendix

调查表编号：______________ 医院名称：

调查员： 调查时间： 年 月 日

一. 个人基本情况：

1. 您的出生日期： _______年_______月_______日或年龄_______
2. 性别：①男 ②女
3. 文化程度：①职高 ②中专 ③大专 ④本科 ⑤硕士及以上
4. 民族：①汉族 ②_________族
5. 婚姻状况：①未婚 ②已婚或同居 ③离异 ④丧偶
6. 职务：①护士长 ②总务护士 ③临床护士 ④医生
7. 职称：①护士 ②护师 ③主管护师 ④副主任护师 ⑤主任护师
8. 编制：①合同 ②在编
9. 平均月经济收入：_________元
10. 已从事目前工作_________年，每天平均工作时间为_________小时

二. 以人为本的护理评估工具(P-CAT)

使用说明

该调查问卷主要用来评价人们在一定（环境）机构内，由工作人员提供的以人为本的护理程度。这份问卷主要包括 13 个关于护理的条目。本次调查需要根据您目前所处机构中的亲身经历，判断以下条目与您自身情况相符的程度，并在您认为描述最为贴切的方框内标记“+”号。请务必回答所有条目。如果您不确定，请选择与之最相近的答案。

该测量工具的开发受到澳大利亚政府、拉筹伯大学、J.O 和 J.R.-医疗和科研资助 (ANZ 受托人)、于默奥大学的支持。

©拉筹伯大学
版权声明。在 1968 年《版权法》和 2000 年《版权修正案（数字议程）法案》中有明确规定，未经书面许可，本出版物的任何部分不得进行复制，存储在任何检索系统或以任何方式（包括电子，机械，缩微，影印，录制或其他方式）传播。可在 s.nugent@latrobe.edu.au 或澳大利亚维多利亚州 1231 丰盛路班杜拉校区 ACEBAC 请求版权许可。

An Australian Government Initiative

LA TROBE UNIVERSITY

UMEÅ UNIVERSITET

	完全不同意<br>1	不同意<br>2	中立<br>3	同意<br>4	完全同意<br>5
1. 我们定期讨论以人为本的护理。	☐	☐	☐	☐	☐
2. 我们有专业的小组讨论护理问题。	☐	☐	☐	☐	☐
3. 我们的正规护理计划中包含了患者的生活史。	☐	☐	☐	☐	☐
4. 医患之间的合作比完成工作更重要。	☐	☐	☐	☐	☐
5. 我们根据患者的需求灵活调整工作。	☐	☐	☐	☐	☐
6. 患者有机会参与到适合个人的日常活动。	☐	☐	☐	☐	☐
7. 我没有时间提供以人为本的护理。	☐	☐	☐	☐	☐
8. 这护理氛围杂乱无章。	☐	☐	☐	☐	☐
9. 我们担心没有舒适的工作环境会对工作质量造成影响。	☐	☐	☐	☐	☐
10. 这个氛围妨碍我提供以人本的护理。	☐	☐	☐	☐	☐
11. 患者的评估居于日常活动。	☐	☐	☐	☐	☐
12. 患者的问题在这里无法解决。	☐	☐	☐	☐	☐
13. 患者可以根据自己的愿望联络外界。	☐	☐	☐	☐	☐

©拉筹伯大学
版权声明。在 1968 年《版权法》和 2000 年《版权修正案（数字议程）法案》中有明确规定，未经书面许可，本出版物的任何部分不得进行复制，存储在任何检索系统或以任何方式（包括电子，机械，缩微，影印，录制或其他方式）传播。可在 s.nugent@latrobe.edu.au 或澳大利亚维多利亚州 1231 丰盛路班杜拉校区 ACEBAC 请求版权许可。
